#  Keto-polymethines: a versatile class of dyes with outstanding spectroscopic properties for *in cellulo* and *in vivo* two-photon microscopy imaging†
†Electronic supplementary information (ESI) available: Spectroscopic measurements details, synthetic procedures and complete characterizations including NMR spectra and SEC chromatography are provided. Radial distributions functions for O_k_–H in methanol and in dichloromethane evaluated from molecular dynamics simulations. See DOI: 10.1039/c6sc02488b



**DOI:** 10.1039/c6sc02488b

**Published:** 2016-08-03

**Authors:** Simon Pascal, Sandrine Denis-Quanquin, Florence Appaix, Alain Duperray, Alexei Grichine, Boris Le Guennic, Denis Jacquemin, Jérôme Cuny, San-Hui Chi, Joseph W. Perry, Boudewijn van der Sanden, Cyrille Monnereau, Chantal Andraud, Olivier Maury

**Affiliations:** a ENS Lyon , Université de Lyon 1 , CNRS Laboratoire de chimie de l'ENS Lyon , UMR 5182 CNRS, 46 allée d'Italie , 69364 Lyon , France . Email: andraud@ens-lyon.fr ; Email: olivier.maury@ens-lyon.fr; b Univ. Grenoble Alpes , Grenoble Institut des Neurosciences , GIN, Inserm , U836 , F-38000 Grenoble , France; c Inserm , Institut Albert Bonniot , U823 , F-38000 Grenoble , France; d Université Grenoble Alpes , IAB , F-38000 Grenoble , France; e Institut des Sciences Chimiques de Rennes , UMR 6226 CNRS , Université de Rennes 1 , 263 Avenue du Général Leclerc , 35042 Rennes Cedex , France; f Laboratoire CEISAM , CNRS 6230 , Université; de Nantes , 2 Rue de la Houssiniére, BP 92208 , 44322 Nantes Cedex 3 , France; g Institut Universitaire de France , 103 Bvd Michelet , 75005 Paris Cedex 5 , France; h Laboratoire de Chimie et Physique Quantiques (LCPQ) , Université de Toulouse III [UPS] and CNRS , 118 Route de Narbonne , 31062 Toulouse , France; i School of Chemistry and Biochemistry , Center for Organic Photonics and Electronics , Georgia Institute of Technology , 901 Atlantic Drive NW , Atlanta , GA 30332-0400 , USA

## Abstract

The synthesis of keto-heptamethine derivatives has been expanded to various new symmetrical and asymmetrical structures, including an unprecedented di-anionic keto-polymethine. The spectroscopic behavior of these new dyes has been systematically and thoroughly investigated, revealing that the formation of hydrogen bond interactions with protic solvents is responsible for a dramatic enhancement of the fluorescence quantum yield in the far-red spectral region. The existence of these strong hydrogen-bond interactions was further confirmed by molecular dynamics simulations. These bis-dipolar polymethines exhibit large two-photon absorption (TPA) cross-sections (*σ* ^2^ in GM) in the near-infrared, making them ideal candidates for NIR-to-NIR two-photon microscopy imaging applications. We demonstrate that the molecular engineering of the hydrophilic/hydrophobic balance enables targeting of different cellular components, such as cytoplasm or cell membranes. Addition of appropriate substituents provides the molecule with high-water-solubility, affording efficient two-photon probes for angiography.

## Abstract 2

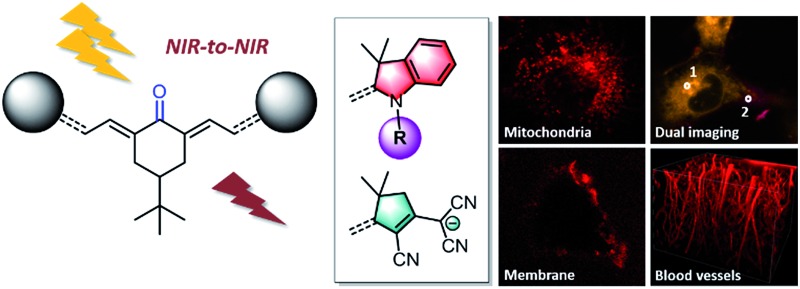
The keto-heptamethine family has been expanded to various symmetrical and asymmetrical structures.

## Introduction

Fluorescence microscopy has become a routine technique in biological research, and linear or non-linear microscopes are now standard equipment in most biological and biomedical research laboratories.^1^ In particular non-linear two-photon microscopy (TPLSM) is generally considered as a non-invasive technique, which allows *in vitro* or *in vivo* cell imaging in tissues, detection of chemical analytes, or monitoring of biological processes with excellent spatial and temporal resolution.^2^ The increasing development of microscopy techniques has invigorated the design of new luminescent probes featuring improved one- or two-photon brightness in biological media.^3^

**Chart 1:**
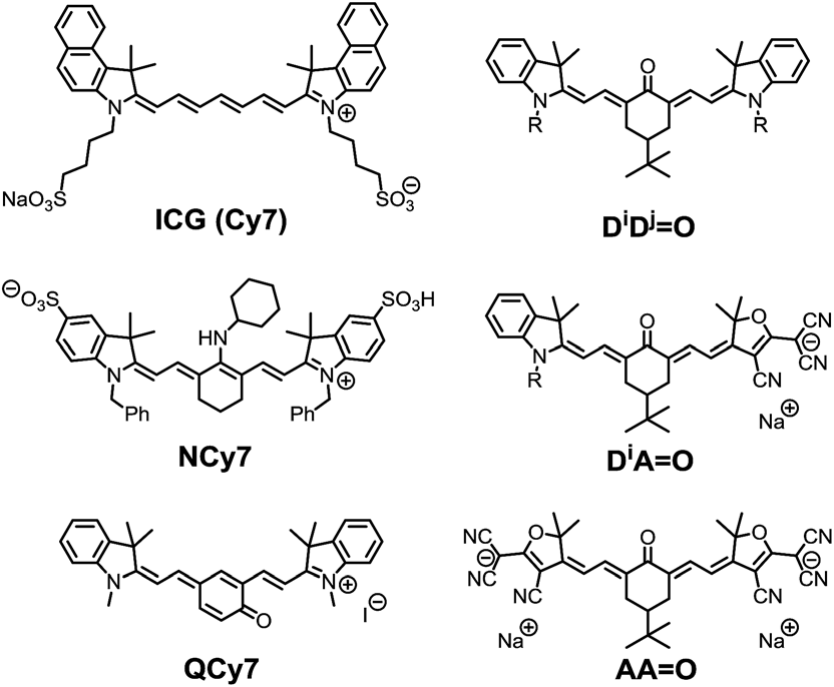
Selected examples of functionalized heptamethine dyes and structure of the keto-heptamethine involved in this study.

Among the broad category of commercially-available, synthetic fluorescent molecules, polymethine dyes such as **Cy5** (pentamethine) or **Cy7** (heptamethine) derivatives, including the popular indocyanine green (**ICG**), are certainly the most commonly used for bio-medical applications (Chart 1).^4^ The key advantages of polymethines are their large molar extinction coefficients (*ε*) and moderate-to-good fluorescence quantum yields (*Φ*), which result in excellent one-photon brightness (defined as the product of the extinction coefficient and the quantum yield, *εΦ*) in the far-red or near-infrared regions. This spectral range is referred to as the “biological transparency window” (BTW: 680–1300 nm), where tissues absorption scattering and autofluorescence are minimized, and thus where most research endeavors are concentrated.^3^ However, polymethine dyes present several inherent drawbacks that may limit their broad application in future bio-imaging protocols. In particular, the *cyanine*-type electronic transitions are associated with very small Stokes shifts (<1000 cm^–1^), which requires filtering of the incident or scattered excitation light and loss of most of the emission signal resulting in a decrease of the signal-to-noise ratio.

In 2005, Peng and co-workers reported the amino-heptamethine dye **NCy7** (Chart 1) featuring a large Stokes shift (*ca.* 3500 cm^–1^) and a high fluorescence quantum yield (*ca.* 40%) in the BTW.^5^ This dye has since been adapted for bio-conjugation, bio-imaging, or FRET experiments and used for bio-sensing of metal ions like Zn^2+^, Cu^2+^, reactive oxygen species, or thiol-containing residues like glutathione.^6^ We recently demonstrated that it was possible to fine tune the electronic structure of heptamethine derivatives by simple nucleophilic substitution reactions on the halogenated central carbon of a common precursor, and to generate a variety of dyes with absorption bands covering the whole UV-visible spectral range.^7^ These results were rationalized on the basis of an alteration of the initial *cyanine* to a *bis-dipolar* ground state electronic configuration. Among these dyes, we became particularly interested in polymethines functionalized by carbonyl moieties at the central carbon position, the so-called keto-polymethine that features excellent one-photon brightness in methanol (*εΦ* = 24 500 L mol^–1^ cm^–1^ at 636 nm, **D*^i^* D*^j^* =O** in Chart 1).^8^

Keto-polymethine dyes were initially reported and studied (both theoretically and experimentally) in the early 2000s.^9^ These chromophores were then used as pH sensors in integrated waveguide devices,^10^ and as NIR electrochemical fluorescence switches.^11^ Surprisingly, the exploration of their potential for bio-imaging applications is scarce. Recently, Shabat and co-workers reported the quinone derivatives (**QCy7**, Chart 1) that enable intravital imaging of hydrogen peroxide.^12^ In all other cases, keto-heptamethines were only reported as the reaction products of polymethine dyes involved in the detection of hydrazine, hydrogen sulfide or cysteine both *in vitro* and *in vivo*.^13^

Herein, we aim to expand the synthesis of keto-polymethine dyes featuring the classical bis-indolenine (**D**) electron-donating end-groups (**D*^i^* D*^j^* =O**) where *i*, *j* designate identical or different substituents of the indolenine moieties. In addition we reported original symmetrically (**AA=O**) or asymmetrically (**D*^i^* A=O**) substituted keto-polymethine dyes containing anionic (**A**) tricyanofuran end-groups acting here as very strong donors (Chart 1). The linear and nonlinear photophysical properties of these new molecules were thoroughly investigated and the results show that these structural modifications lead to major changes in their spectroscopic properties, which makes it possible to achieve very high one- or two-photon brightness in the BTW. In all cases, experimental and theoretical evidence indicates that hydrogen-bonding effects induce a strong increase of the fluorescence quantum yield. The hydrophilic/hydrophobic balance of these chromophores was then modulated by tuning the substituents on the indolenine fragment (alkyl chains, sulfonate moieties, hydrosoluble polymers), and the resulting water-soluble dyes were employed in bio-imaging experiments. We demonstrate that lipophilic dyes can be rapidly internalized into living cells and stain cytoplasm organelles, whereas amphiphilic derivatives are primarily localized in the plasma membrane. The most hydrophilic dyes are not internalized, but are advantageously used as two-photon probes for imaging the cerebrovascular structures of mice in a NIR-to-NIR configuration, thus expanding the scope of keto-polymethines as fluorescent biomarkers.

## Results and discussion

### Synthesis

**Scheme 1:**
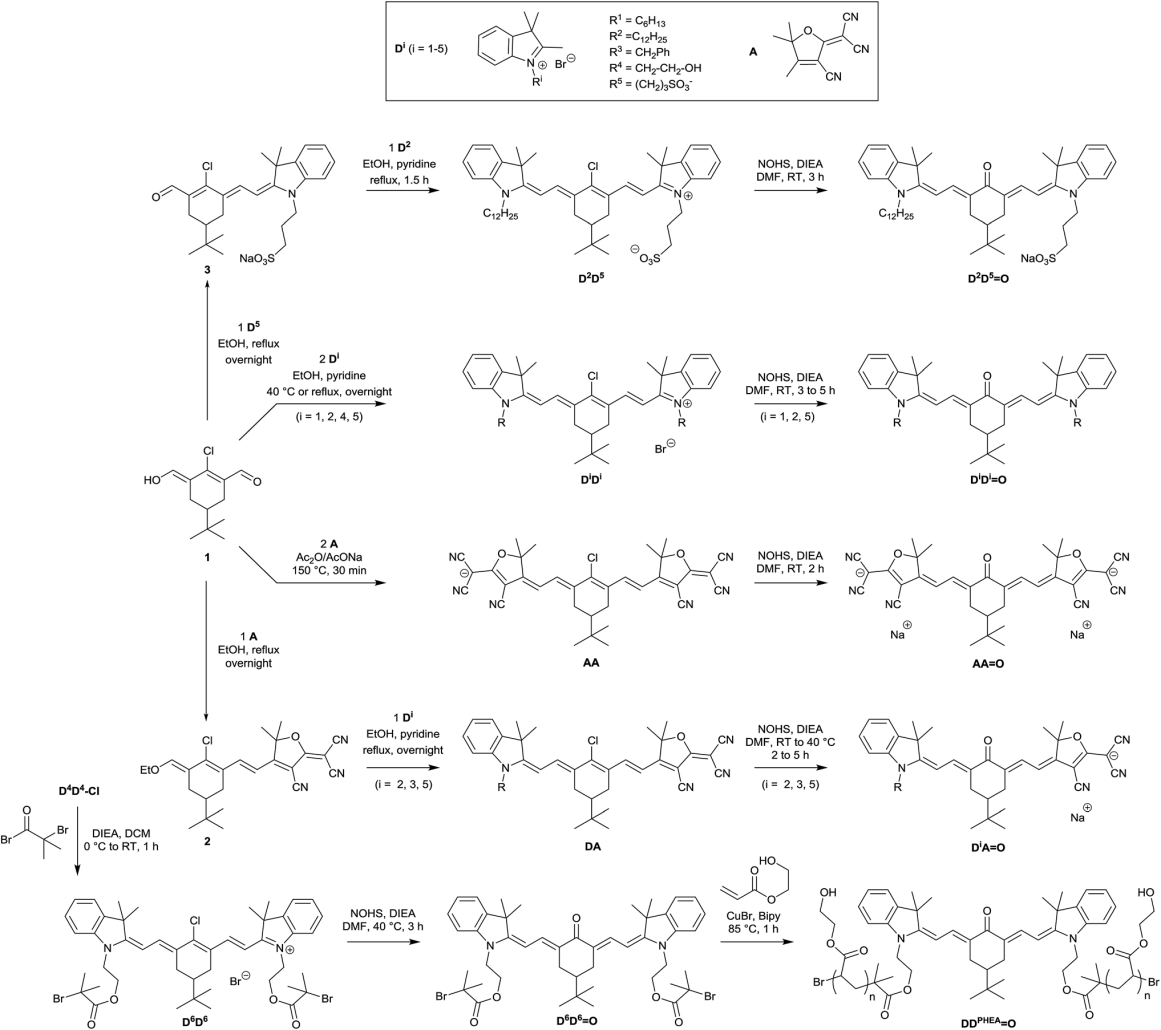
Synthetic route for the preparation of keto-polymethine derivatives.

The synthetic route towards all studied molecules is outlined in Scheme 1 and described in detail in the Experimental section. Briefly, every keto-heptamethine derivative was synthesized following the general strategy reported by Strekowski *et al.* ^9*c*^ relying on a key nucleophilic substitution step on the central position of the corresponding chlorinated heptamethine. For convenience, in what follows, chloro-heptamethines will be referred to as **D*^i^* D*^j^*** depending on the nature of the terminal substituents on both extremities of the molecule, and on the substitution of the indolenine fragment (**D*^i^*** with *i* = 1–5, Scheme 1).

The starting chlorinated cyanines were classically obtained through a Knoevenagel reaction in basic anhydrous conditions between the chlorinated bis-aldehyde **1** and various indolenium salts (**D^1^** to **D^5^**). For the latter, *N*-alkylations on the indolenium ring were used to obtain molecules with a range of different hydrophilic/lipophilic characteristics (see Scheme 1 and the Experimental section). The unsymmetrical chloro-heptamethine **D^2^D^5^** precursor featuring amphiphilic behavior (one sulfonate and one lipophilic extremity) was prepared in two successive Knoevenagel condensations: (i) the enol derivative **3** was obtained in 74% yield by reaction of bis-aldehyde **1** and the indolenium precursor **D^5^** at 80 °C in the absence of base; (ii) the second indolenium **D^2^** was further added and the second condensation was achieved at 80 °C in the presence of pyridine as a base. The water-soluble keto-heptamethine **DD^PHEA^ =O**, containing hydrophilic polymer chains was prepared following our previously reported methodology.^14^ This functionalization was achieved through the **D^4^D^4^** intermediate that features bis-hydroxyl functionalities enabling the subsequent introduction of bromoisobutyryl groups (**D^6^D^6^**). The corresponding keto-derivative, **D^6^D^6^ =O**, was prepared and the bromoisobutyryl moieties were finally used as initiators for the living Atom Transfer Radical Polymerisation (ATRP) reaction of 2-hydroxyethylacrylate monomer.^14,15^ After purification of the resulting chromophore-polymer by dialysis, the ^1^H NMR of **DD^PHEA^ =O** allowed us to calculate an average degree of polymerization of *n* = 80 at both extremities of the chromophore. 2D diffusion-ordered NMR spectroscopy (DOSY) was used to estimate a polydispersity index (PDI) for this compound, following the methodology initially developed by Delsuc and collaborators.^16*a*^ A PDI = 1.09 was calculated, highlighting the remarkable control provided by ATRP (see Fig. S1†). The estimation of PDI using gel permeation chromatography techniques (GPC) resulted in a slightly higher value (PDI = 1.39, see Fig. S2†). By analogy with previous studies,^14,16^ the observed discrepancies between the two techniques were attributed to the branched nature of the polymer chains that leads to an overestimation of polydispersity indexes by GPC.^17^ A minimal length of 80 units was found to be necessary for the target biological application, as our attempts to produce shorter chains resulted in a non-water soluble material.

The synthesis of heptamethine **AA** was readily achieved by a Knoevenagel reaction between **1** and the tricyanofuran derivative **A** in acidic conditions, following published procedures.^18^ Finally in the case of unsymmetrical heptamethines, the chlorinated precursors **D*^i^* A** were synthesized following our previously reported methodology,^19^ consisting of two successive Knoevenagel reactions on **1**, first with the tricyanofuran withdrawing group in order to form **2**, then with one of the indolenium salt derivatives (**D*^i^***). It is particularly remarkable that the formation of the ketone, which has been reported so far exclusively from cationic derivatives (**D*^i^* D*^i^***), was straightforwardly adaptable from both the neutral (**D*^i^* A**) and even anionic (**AA**) analogues, with identical reaction conditions and similar high yields. Using this approach, we were able to synthesize the entire series of chromophores depicted in Scheme 1. It is worth noting that compounds **D*^i^* D*^j^* =O** (*i*, *j* = 1–5) are neutral whereas **D*^i^* A=O** and **AA=O** become anionic and di-anionic, respectively as unambiguously demonstrated by mass spectrometry analysis. All target chromophores and new intermediates were fully characterized by ^1^H and ^13^C NMR and High-Resolution Mass Spectroscopy (HRMS). In the case of **D*^i^* A=O** heptamethines, broadening (and, in one case, splitting) of the ^1^H and ^13^C NMR peaks could be assigned to *cis*-to-*trans* isomerizations of the non-cyclic double bonds of the conjugated backbone that operates on the NMR time scale.^18^ However, ^1^H NMR experiments recorded at low (218 K) and high (368 K) temperatures did not allow observation of the coalescence of broad and split signals (see ESI†).

### Photophysical properties

**Fig. 1:**
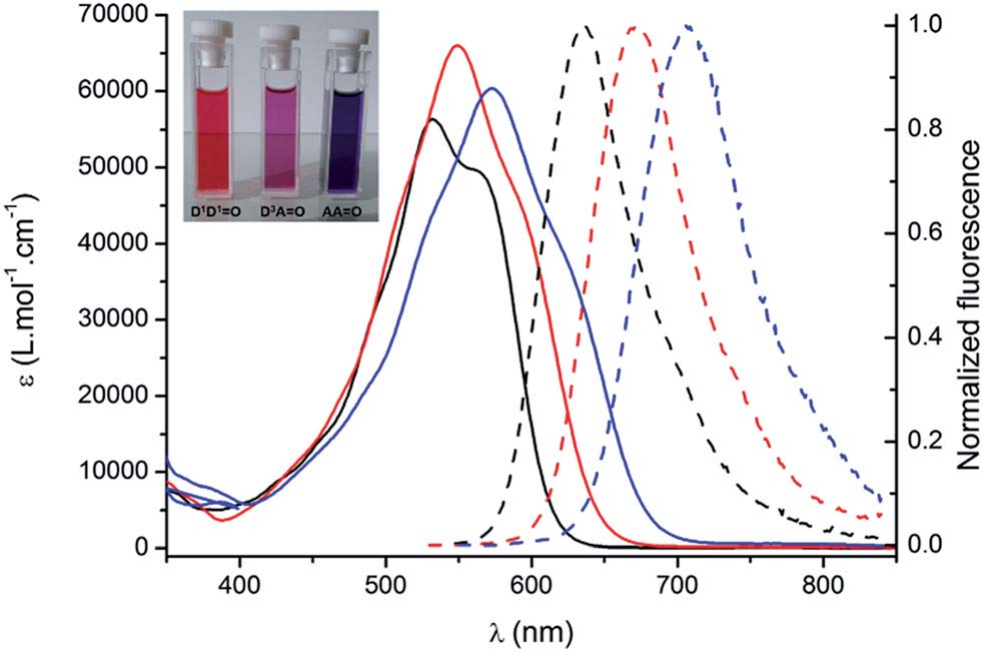
Absorption (plain lines) and emission (broken lines) spectra of **D^1^D^1^ =O** (black), **D^3^A=O** (red) and **AA=O** (blue) in methanol. Inset: typical coloration of methanol solutions.

**Fig. 2:**
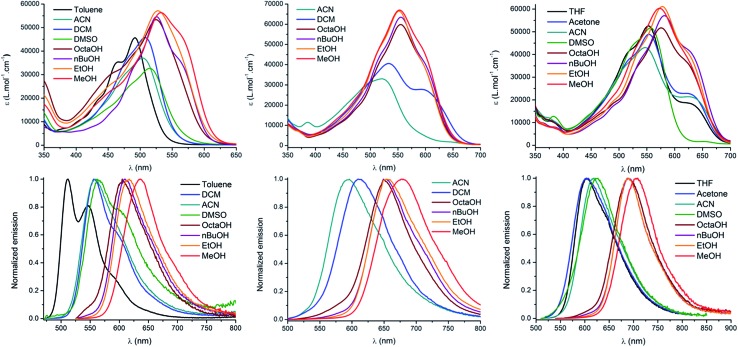
Absorption (top), emission (bottom) and solvatochromism of **D^1^D^1^ =O** (left), **D^2^A=O** (middle) and **AA=O** (right).

The spectroscopic properties of the lipophilic keto-heptamethine derivatives (**D^1^D^1^ =O**, **D^3^A=O**, **AA=O**) featuring different substituents were studied in a variety of solvents, in order to evaluate their potential as two-photon fluorescent markers for biological applications. Representative absorption and emission spectra of **D*^i^* D*^j^* =O**, **D*^i^* A=O** and **AA=O** keto-chromophores in methanol are presented in Fig. 1. The optical properties in different solvents are displayed in Fig. 2 and Table 1, while Table 2 summarizes the data in methanol (or water, when available, *vide supra*) for all the new keto-derivatives.

**Table 1:** Optical properties in acetonitrile and water

Solvent	**D^1^D^1^ =O**			**D^2^A=O**			**AA=O**		
	*λ*_abs_ (nm)	*λ*_em_ (nm)	*φ* ^*a*^ (%)/*τ* (ns)	*λ*_abs_ (nm)	*λ*_em_ (nm)	*φ* ^*a*^ (%)/*τ* (ns)	*λ*_abs_ (nm)	*λ*_em_ (nm)	*φ* ^*a*^ (%)/*τ* (ns)
PhMe	492	512	4^*b*^/—^*c*^	—	—	—	—	—	—
CH_3_CN	515	558	3^*b*^/—	521	596	1^*b*^/—	547	619	6^*b*^/—
CH_2_Cl_2_	508	556	5^*b*^/—	533	610	3^*b*^/—	—	—	—
(CH_3_)_2_SO	515	562	3^*b*^/—	—	—	—	557	627	8^*b*^/—
C_8_H_17_OH	525	605	25/1.2	555	649	46/1.8	576	690	40/1.4
C_4_H_9_OH	526	610	36/1.3	555	653	58/1.7	581	687	45/1.9
C_2_H_5_OH	528	617	40/1.4	552	653	61/1.6	578	690	42/1.4
CH_3_OH	532	636	44/1.6	553	678	53/1.4	573	708	33/1.0

^*a*^Rhodamine B as reference (*Φ* = 66% in CH_3_OH).

^*b*^Rubrene as reference (*Φ* = 27% in CH_3_OH).

^*c*^Not measured because the lifetime is below our detection limit (0.5 ns).

**Table 2:** Photophysical properties of the new keto-heptamethines

Compound	Solvent	*λ*_abs_ (nm)	*ε* (L mol^–1^ cm^–1^)	*λ*_em_ (nm)	Stokes shift (cm^–1^)	*φ* ^*a*^ (%)	*τ* ^*b*^ (ns)
**D^1^D^1^ =O**	MeOH	532	56 000	636	3070	44	1.6
**D^2^D^2^ =O**	MeOH	531	54 000	634	3060	50	1.6
**D^2^D^5^ =O**	MeOH	530	67 000	633	3070	42	1.6
**D^5^D^5^ =O**	MeOH	527	35 000	633	3180	54	1.6
	H_2_O	567	38 000	645	2130	28	0.9
**D^6^D^6^ =O**	MeOH	522	58 000	624	3130	51	1.5
**DD^PHEA^ =O**	MeOH	525	—	625	3050	79	1.7
	H_2_O	541	—	629	2590	32	1.8
**D^2^A=O**	MeOH	552	67 000	672	3240	43	1.4
**D^3^A=O**	MeOH	549	66 000	673	3360	54	1.6
**D^5^A=O**	MeOH	551	67 000	672	3270	37	1.4
	H_2_O	574	35 000	679	2690	7	—
**AA=O**	MeOH	573	60 000	706	3290	33	1.0
**D^1^D^1^–OH** ^*c*^	MeOH	712	99 000	735	440	43	1.1
**D^3^A–OH** ^*c*^	MeOH	757	160 000	782	420	18	0.7
**AA–OH** ^*c*^	MeOH	804	111 000	837	490	N/A^*d*^	—

^*a*^Rhodamine B as reference (*Φ* = 66% in MeOH) for keto-derivatives and IR-125 as reference (*Φ* = 13% in DMSO) for hydroxy-derivatives.

^*b*^NanoLED excitation at 490 nm for keto-derivatives and at 732 nm for hydroxy-derivatives.

^*c*^ *In situ* formation by addition of concentrated acetic acid.

^*d*^Luminescence was too weak to determine fluorescence quantum yield and lifetime.

Compared to the chlorinated parent molecules, which present a typical cyanine-type absorption band, all keto-derivatives exhibit a distinctive broad and almost structureless absorption band. These transitions clearly present a marked charge transfer (CT) character, typical for bis-dipole-type electronic structure that is a general hallmark for the keto-substitution.^7^ As noted in ref. 7, the absorption band showed systematic hypochromic and hypsochromic shifts compared to the corresponding chloro-heptamethine chromophores. Molar extinction coefficients are relatively similar within the series, with values in the range of 50 000–60 000 L mol^–1^ cm^–1^. The transition energies vary in the order **D*^i^* D*^j^* =O** > **D*^i^* A=O** > **AA=O**, which is associated with the nature of the terminal substituents (Fig. 1).

The absorption properties of keto-heptamethines are highly sensitive to the protic or aprotic nature of the solvent. As shown in Fig. 2, the position of the absorption band is only little dependent on solvent polarity; conversely a significant red-shift is observed between aprotic and protic solvents. As an example, the absorption in octanol is red-shifted compared to that in a more polar solvent like DMSO. This can be almost certainly correlated to the formation of hydrogen bonding between the chromophore and protic solvent molecules with the keto-heptamethine acting as the hydrogen bond acceptor (*vide supra*). This hypothesis is discussed thoroughly and proved true in the theoretical calculation section.

The same dependence on the solvent acidity (protic or aprotic nature) is observed in the emission spectra. In non-protic solvents, all compounds show a relatively classical emission profile evolution, *i.e.* a red shift of the emission upon increasing solvent polarity (positive emission solvatochromism), which comes with a loss of its fine vibronic structure. This is a typical signature of a fluorescence emission arising from a CT excited state. In contrast, a different trend is observed in protic solvents: the emission becomes much narrower and is strongly red-shifted by up to 124 nm (3810 cm^–1^) for **D^1^D^1^ =O**, even as compared to the most polar aprotic solvent (DMSO). It is also remarkable that the position of the band in protic solvents is only weakly affected by their polarity. Indeed, for both **D^1^D^1^ =O** and **D^2^A=O**, a comparatively modest 30 nm red-shift (805 and 660 cm^–1^ respectively) is seen between octanol and methanol. Again, hydrogen bonding constitutes the only reasonable explanation to account for the observed behavior and the hydrogen-bonding effect is discussed below. The most remarkable effect associated with protic solvents concerns the emission quantum yield and lifetime of the chromophores (Table 1). Whereas all of the molecules in this study are poorly fluorescent in non-protic solvents (*Φ* < 5% for **D*^i^* D*^j^* =O**), their quantum yields increase spectacularly up to 50% in protic ones, which constitutes an exceptionally high value, in the far-red (**D*^i^* D*^j^* =O**) and near-infrared (**AA=O**) spectral range. The fluorescence lifetimes follow the same trend: the mono-exponential decay is particularly short in non-protic solvents (*τ* < 0.5 ns) and increased to 1.2–1.8 ns in protic solvents. These observations constitute a clear indication that the non-radiative relaxation pathways are disfavored in protic solvents, where hydrogen bonding with the solvent occurs.

### Effect of hydrogen bonds

**Fig. 3:**
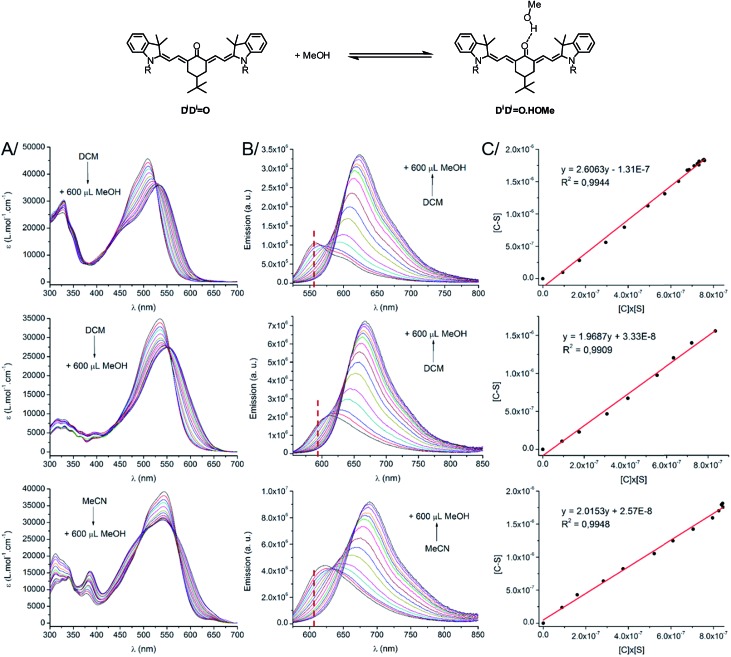
(Top scheme) H-bond equilibrium in the case of **D^1^D^1^ =O**. (Bottom) (A) Absorption, (B) emission spectra and (C) plot of [C–S] *vs.* [C] × [S] for **D^1^D^1^ =O** (top) **D^2^A=O** (middle) and **AA=O** (bottom). The wavelength used for the constant determination is indicated by a red dashed vertical line on the emission spectra.

As stated above, the protic nature of the solvent plays a dramatic role on the photophysical properties of all keto-derivatives. We hypothesized that this effect results from a hydrogen bonding process, where the keto-chromophores and the protic solvent play the roles of hydrogen bond acceptor and donor, respectively, following the equilibrium depicted on top of Fig. 3.

In order to test this hypothesis, titration experiments were performed with the **D^1^D^1^ =O**, **D^2^A=O** and **AA=O** chromophores, following a classical spectroscopic titration protocol, as detailed in the ESI.† As shown in Fig. 3, a progressive decrease of the absorption and emission bands assigned to the “hydrogen bond-free” form (noted C) was observed. It was accompanied by a concomitant increase of the corresponding absorption and emission bands from the “hydrogen bonded” chromophore (noted C–S). The evolution of the intensity of the emission band associated to C upon addition of methanol could be used to calculate, at each point, the concentration of the free dye [C], the H-bond adduct [C–S] and free methanol [S]. A linear correlation was obtained upon plotting [C–S] *versus* [C] × [S], for which the slope corresponds to the association constant *K*_a_ (Fig. 3C, for more information about the methodology, see ESI†). In each case, the three titration experiments could be fitted with a 1 : 1 binding isotherm with a very good accuracy (*R* ^2^ > 0.99) confirming that the spectral changes observed in protic solvents originate from hydrogen bonds between the solvent and the keto-chromophore. *K*_a_ values of 2.61, 1.97 and 2.02 L mol^–1^ (*i.e.* 2.4, 1.65 and 1.75 kJ mol^–1^, respectively) were found for **D^1^D^1^ =O**, **D^2^A=O** and **AA=O**, respectively. These association constants are rather low but support the occurrence of hydrogen bonding between the keto-heptamethines and protic solvents.

**Fig. 4:**
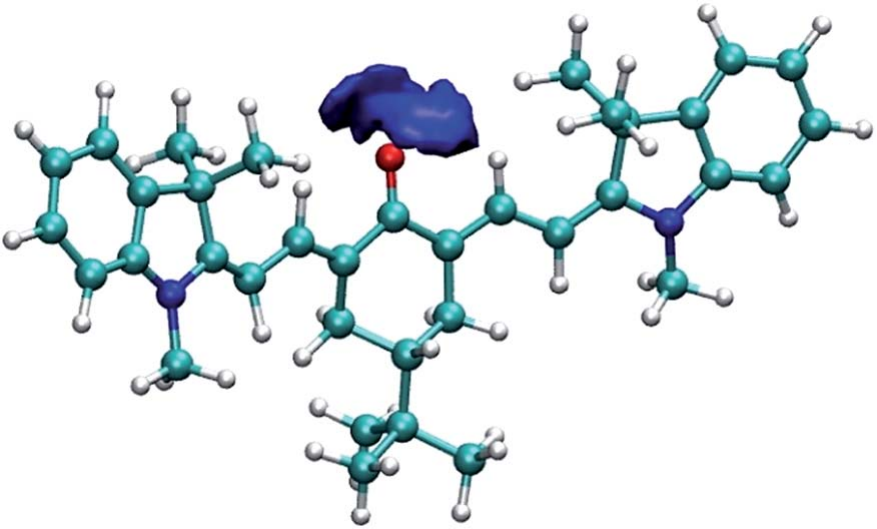
Isosurface of the three-dimensional density map describing the localization of the hydrogen atoms around the oxygen atom of **DD^Me^ =O** in methanol.

To further illustrate and provide a molecular scale description of the interaction of keto-polymethine dyes with the surrounding solvent molecules, we performed molecular dynamics (MD) simulations of the model **DD^Me^ =O** (*i.e.* with R^i^ = CH_3_) in methanol and dichloromethane (see computational details in the Experimental section). From these simulations, we extracted the radial distribution functions for O*–H (O* refers to the oxygen of the keto-heptamethine carbonyl moieties) in the two solvents. These data are presented in Fig. S3.† The O*–H radial distribution function in methanol displays a sharp peak at ∼1.8 Å. This confirms the suggested strong interaction between the methanol molecules and **DD^Me^ =O** that is much weaker in the dichloromethane solution. This is more clearly highlighted in Fig. 4 which shows the strong localization of hydrogen atoms in the vicinity of the O* atom. This confirms the involvement of hydrogen bonds between the chromophore and protic solvents.

### pH effect

**Fig. 5:**
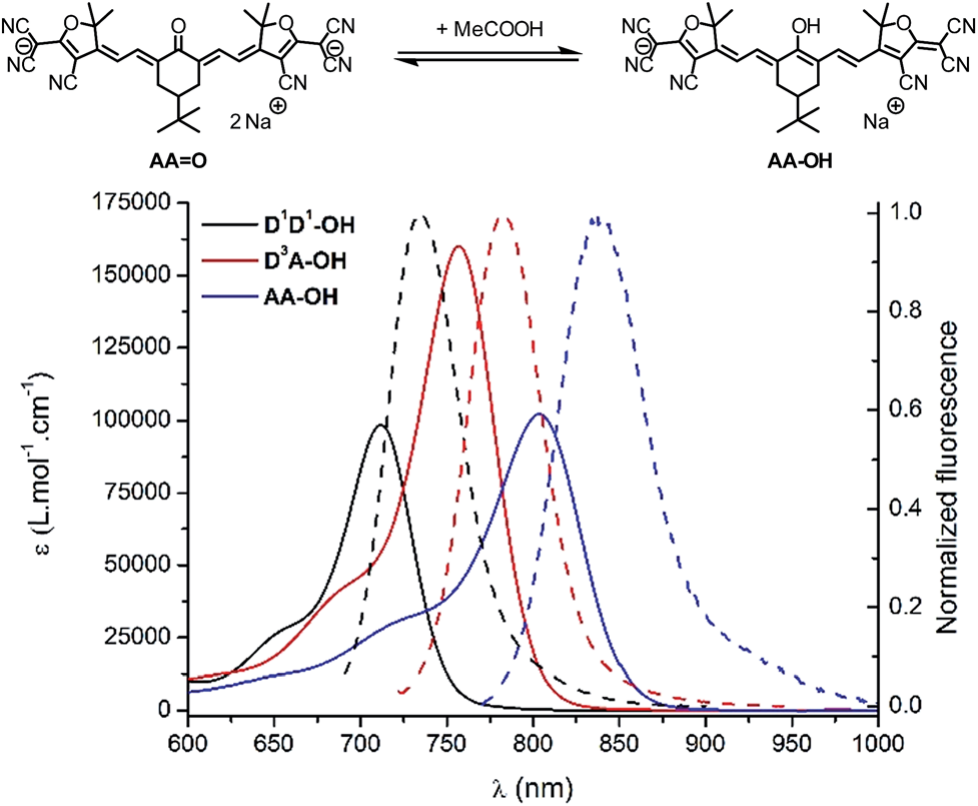
(Top scheme) Protonation equilibrium in the case of **AA=O**. (Bottom) Absorption (plain) and emission (dash) spectra of **D^1^D^1^–OH** (black), **D^3^A–OH** (red) and **AA–OH** (blue), the protonated forms of **DD^1^ =O**, **D^3^A=O** and **AA=O**, in methanol.

The strong dependency of the absorption and emission spectra of all keto-derivatives on the solvent's proticity and hydrogen bonding effects led us to investigate the behavior of these dyes upon protonation. Protonation of keto-polymethines have been reported already in the case of **DD=O** analogs^9*a*^ leading to the formation of the cationic hydroxyl derivative (**DD–OH**) featuring cyanine-type spectroscopic properties.^7^ A similar study was first performed on **D^1^D^1^ =O** and next generalized to **D^3^A=O** and **AA=O**. Upon addition of a drop of acetic acid into diluted methanolic solutions of **D^1^D^1^ =O**, **D^3^A=O** and **AA=O**, a dramatic modification of their absorption and emission spectra was recorded (Fig. 5).

As can be seen from the characteristic shape of the absorption and emission bands, the cyanine character of all molecules is fully restored upon protonation. As a result, both the absorption and emission maxima are strongly red-shifted, *e.g.* to 900 nm for the absorption of **AA–OH**, and the Stokes shifts are strongly decreased compared to the corresponding keto-derivatives. Unfortunately, all protonated forms (**D^1^D^1^–OH**, **D^3^A–OH** and **AA–OH**) exhibit a poor stability in solution, as illustrated by the relatively rapid decrease of the cyanine absorption band. This instability precluded further quantitative study on these molecules, such as p*K*_a_ measurements and, *a fortiori*, their use as pH sensitive probes.

### Nonlinear optical properties

**Fig. 6:**
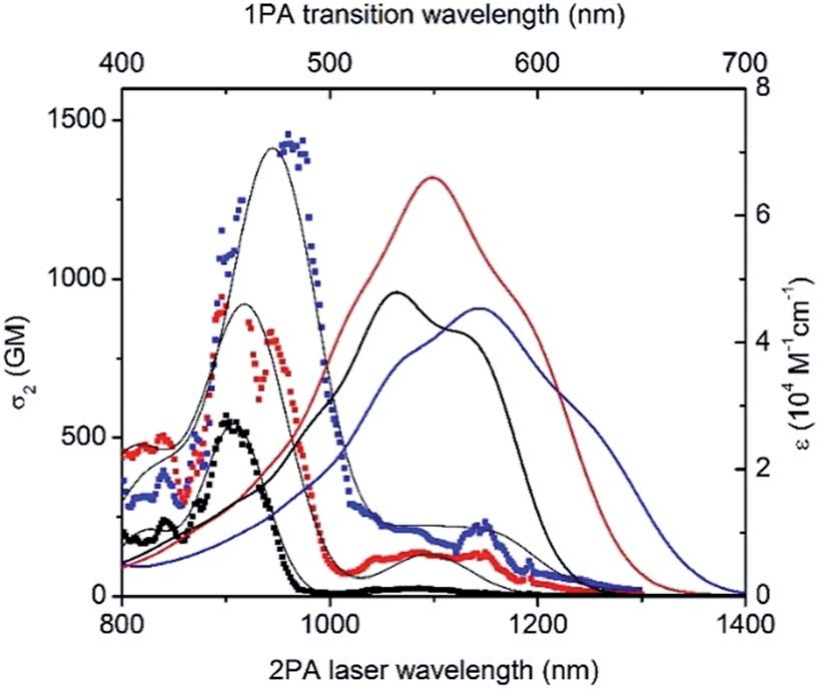
Two-photon absorption spectra of **D^1^D^1^ =O** (black), **D^3^A=O** (red) and **AA=O** (blue) keto-derivatives in methanol (dotted). Black lines were generated from the peak fit to guide the eyes. Linear spectra (solid lines) are included in the upper scale for comparison.

Besides having significant luminescence properties in the NIR, a chromophore should also display intense TPA to be considered as a relevant probe for two-photon microscopy in a NIR-to-NIR configuration. The TPA spectra of **D^1^D^1^ =O**, **D^3^A=O** and **AA=O** were mapped using the two-photon excited fluorescence (TPEF)^20^ and, due to the lack of a stable and reliable TPEF standard in NIR, the magnitude of cross sections were calibrated with open-aperture *z*-scan techniques.^21^ As a general feature, all keto-heptamethines present similar TPA spectra composed of two transitions (Fig. 6). The lowest energy TPA band is weak and matches well the wavelength-doubled one-photon absorption spectra. On the other hand, the transition energy of an intense TPA band is significantly blue shifted compared to one-photon transition energy. These spectral characteristics are generally associated with centrosymmetric quadrupolar molecules, in which selection rules for one- and two-photon absorption electronic transitions are mutually exclusive. In the case of non-centrosymmetric molecules, the transition energy of TPA and OPA generally overlap. In the present study, all keto-heptamethine dyes are non-centrosymmetric since they present either a quasi-*C*_2v_ symmetric CT-dipolar (**D^3^A=O**) or *C*_2v_ symmetric CT-bis-dipolar (**D^1^D^1^ =O** and **AA=O**) ground state electronic structure. Such molecules with *C*_2v_ or quasi-*C*_2v_ symmetries can be treated as bent quadrupolar molecules, of which the quadrupolar character results in intense, blue-shifted TPA while the residual dipolar character gives a weak TPA band that generally overlaps with the one-photon transition. Similar observations were recently reported by Perry and co-workers, including diarylboryl dyes and extended Michler's ketone derivatives, a class of molecules that are closely related to keto-heptamethines.^22^ In our case, remarkably large two-photon efficiencies were measured for the whole series, with two-photon cross section (*σ* ^2^) values ranging from *ca.* 570 to 640 GM at 900 and 944 nm for **D^1^D^1^ =O** and **D^3^A=O**, respectively. In the case of the di-anionic ketone **AA=O**, a TPA cross-section of 1400 GM was recorded at 960 nm, which constitutes a high value that is comparable with other TCF-heptamethines reported previously.^21*a*^

### Water-soluble keto-heptamethines

The detailed spectroscopic study of the lipophilic probes allowed us to identify interesting candidates as two-photon fluorescent probes. In particular, all measured keto-heptamethines present high two-photon brightness (*σ* ^2^ *Φ*) of *ca.* 250, 345 and 460 GM in methanol for **D^1^D^1^ =O**, **D^3^A=O** and **AA=O**, respectively, with two-photon absorption and emission maxima both located in the red–NIR spectral range. Consequently, these chromophores are ideal candidates for two-photon imaging microscopy in the NIR-to-NIR microscopy configuration. In order to test these chromophores in the context of cellular imaging and *in vivo* vascular imaging, it was first necessary to render the selected probes water-soluble and biocompatible. To that end, two types of functionalization of the indole moieties were considered using (i) sulfonate groups (**D^5^**) or (ii) water-solubilizing polymers (**D^PHEA^**).^14*b*^

**Fig. 7:**
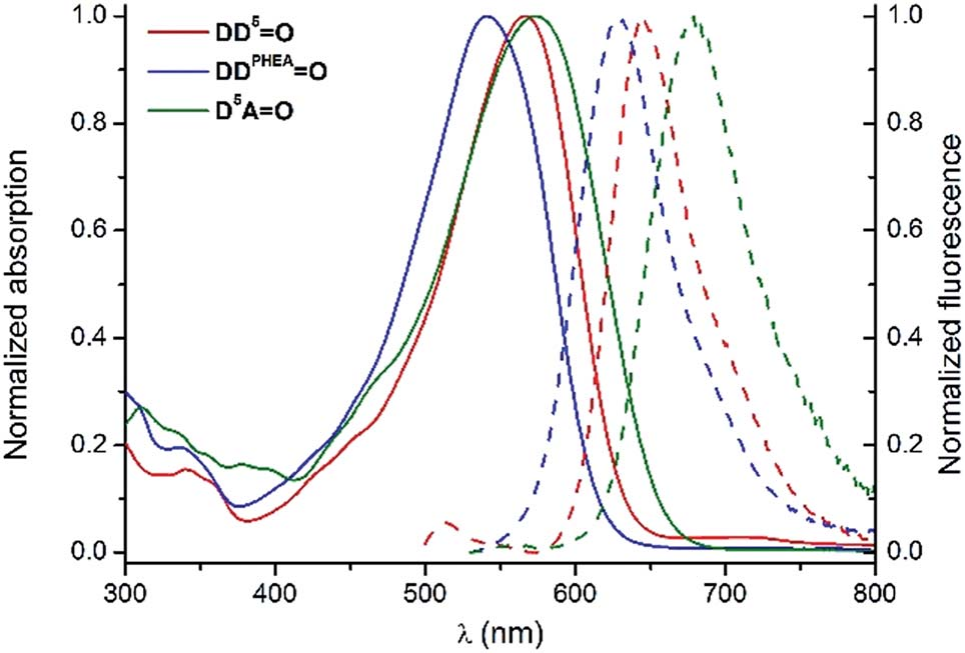
Normalized absorption (plain lines) and emission (dashed lines) spectra of **D^5^D^5^ =O** (red), **DD^PHEA^ =O** (blue) and **D^5^A=O** (green) in H_2_O.

For **D*^i^* D*^j^* =O** derivatives (Scheme 1), functionalization could be introduced either symmetrically on both indolenium groups leading to the formation of **D^5^D^5^ =O** and **DD^PHEA^ =O** or selectively at one extremity providing the amphiphilic **D^2^D^5^ =O** and **D^5^A=O** derivatives. The photophysical properties of these chromophores in water were then investigated, except for **D^2^D^5^ =O**, which did not display sufficient water-solubility to allow this study. For all water-soluble dyes, the shapes and positions of the absorption and emission bands are very similar to those obtained in polar protic solvents, which indicates systematic occurrence of hydrogen bonding with water (Fig. 7). In the case of **D^5^A=O**, a severe drop in the emission efficiency was observed in water (*Φ* = 7%, *vs.* 37% in MeOH). This may be due to an aggregative quenching of the emission related to the low solubility of the molecule in the medium. In contrast, the two hydrophilic molecules **D^5^D^5^ =O** and **DD^PHEA^ =O** conserved good quantum yields of *ca.* 30% in water, which constitute high values for red emitting water-soluble fluorophores.

### Cellular imaging

**Fig. 8:**
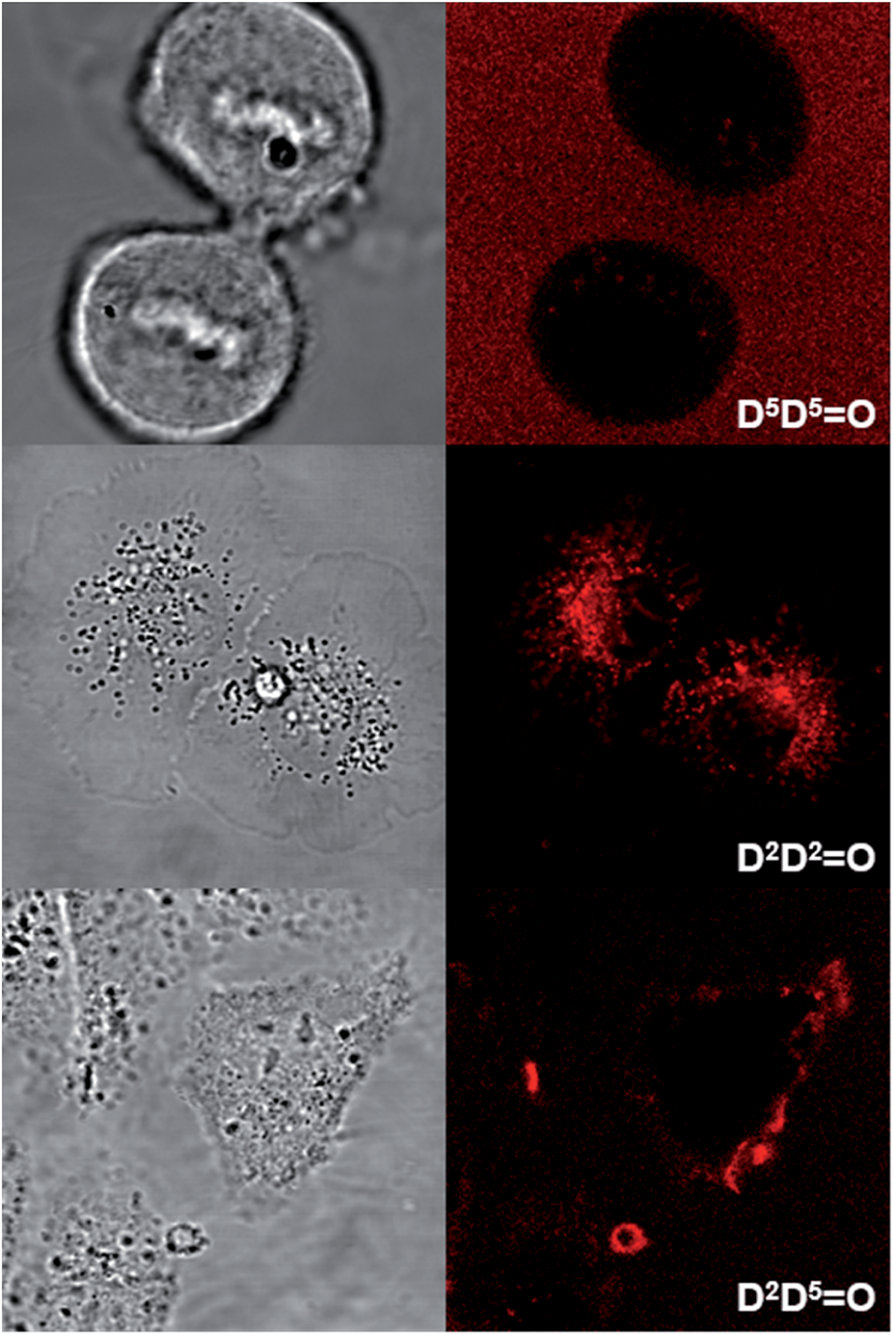
Transmission (left) and two-photon fluorescence (right) microscopy images of T24 cancer cells after their incubation with **D^5^D^5^ =O** (top), **D^2^D^2^ =O** (middle) and **D^2^D^5^ =O** (bottom). Two-photon excitation was performed at 950 nm.

**Fig. 9:**
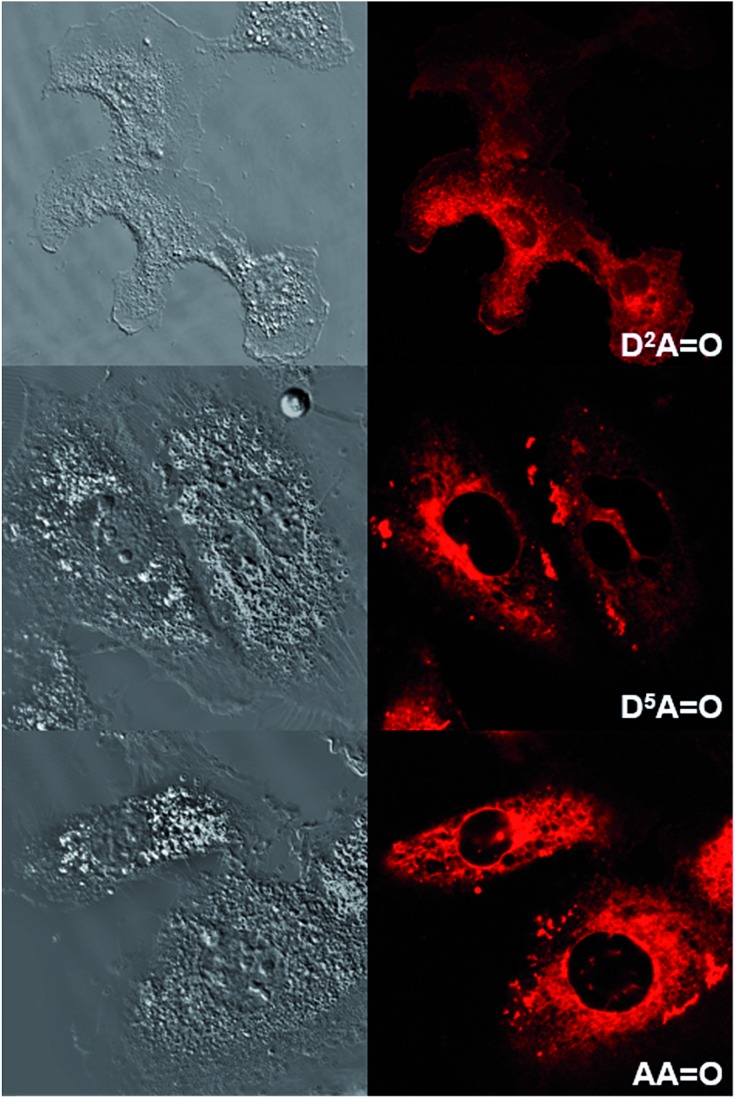
Transmission (left) and one-photon fluorescence (right) microscopy images of T24 cancer cells after their incubation with **D^2^A=O** (top), **D^5^A=O** (middle) and **AA=O** (bottom). One photon excitation was performed at 561 nm.

Human T24 cancer cells were imaged with keto-heptamethine dyes featuring various hydrophilic/hydrophobic groups in order to evaluate the influence of the chromophore structure (charge, substitution) on the internalization and localization processes. To this end, seven dyes with either hydrophilic (**D^5^D^5^ =O**, **DD^PHEA^ =O**), hydrophobic (**D^2^D^2^ =O**, **D^2^A=O** and **AA=O**) or amphiphilic (**D^2^D^5^ =O** and **D^5^A=O**) character were compared. All cells were imaged using either two-photon (Fig. 8 and S4†) or one-photon fluorescence confocal microscopies (Fig. 9 and S5†). With both techniques, highly contrasted images were obtained for all probes.

It can be noted that for the highly hydrophilic dyes that are either neutral (**DD^PHEA^ =O**) or dianionic (**D^5^D^5^ =O**), no internalization in the cells was observed. The chromophores stayed in the extracellular medium and the cells appeared as dark spots surrounded by a fluorescent medium (Fig. 8 and S5†). This feature is clearly detrimental for specific cellular imaging, but is potentially of interest for other imaging applications, as illustrated below. On the other hand, highly lipophilic dyes like neutral **D^2^D^2^ =O** or monoanionic **D^2^A=O** in DMSO solution were rapidly internalized in cells. The chromophores preferentially stained organelles in the cytoplasm and nucleoli inside the nucleus. Interestingly, **AA=O** presents the same behavior indicating that, in spite of its dianionic charge, this dye displays a strong lipophilic character (Fig. 9). This result can be explained by the strong charge delocalization over the entire C_sp^2^_-skeleton.^7,18^ Similarly the anionic charge delocalization of **D^2^A=O** also results in an overall lipophilic character and rapid cell penetration. On the other hand, the amphiphilic dyes (**D^2^D^5^ =O**, **D^2^A=O**) showed an intermediate behavior. The culture medium remained weakly fluorescent indicating that internalization was not complete and accumulation was mostly observed in the external membranes (**D^2^D^5^ =O**) and, to a lesser extent, in the cytoplasm (**D^2^A=O**).

**Fig. 10:**
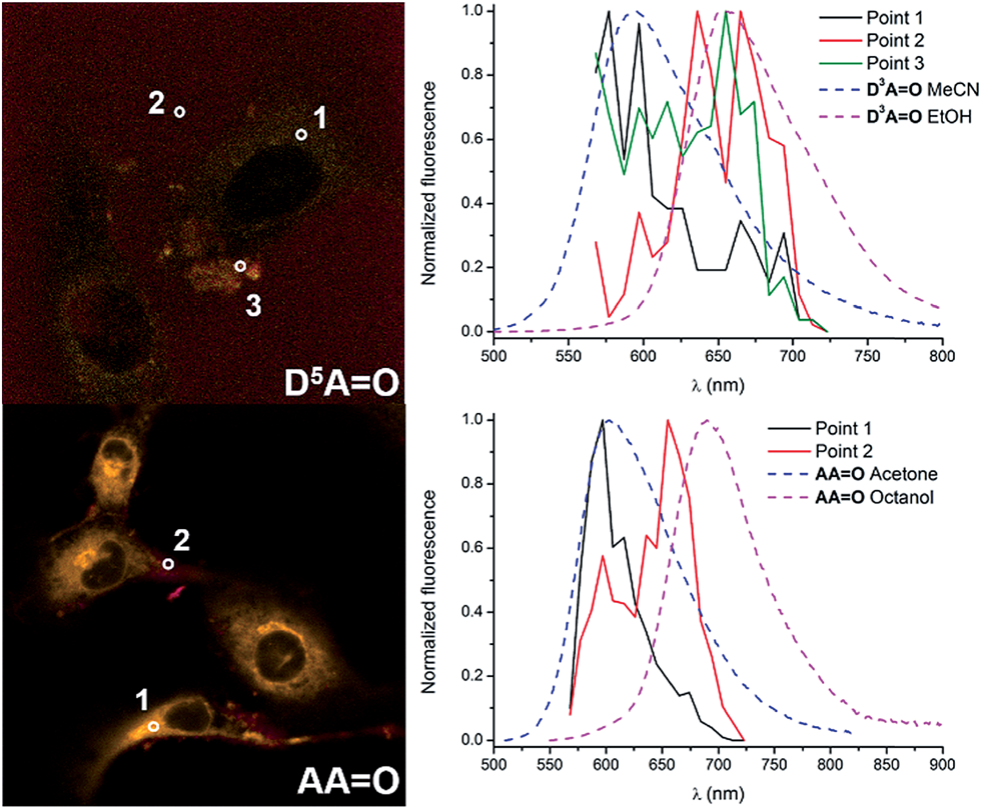
Fluorescence microscopy images of **D^5^A=O** (top) and **AA=O** (bottom) reconstructed from the spectral analysis. The corresponding graphs on the right are the comparison of the emission extracted from different points and the reference dyes in protic or non-protic solvents.

The emission profiles were recorded within the cells incubated with keto-dyes (**D^5^A=O** and **AA=O**) in different channels from 570 to 720 nm (Fig. 10). Interestingly, in both cases, the shape of the spectra consists of two emission bands whose relative intensity depends on the local environment. Comparison with emission spectra recorded in methanol or acetonitrile clearly indicates that these two contributions correspond to emission of the H-bonded and H-bond free chromophores. In the case of **D^5^A=O**, a hydrogen-bond free form of the dye is exclusively seen in the cytoplasm organelles (point 1), with an emission peak around 580 nm (the shape of the signal coincides with the emission of **D^2^A=O** in acetonitrile reported in Fig. 2 and Table 1). In contrast, the signal extracted from the culture media (point 2) exhibits a behavior close to **D^2^A=O** in ethanol, where hydrogen bonding leads to a red-shift of the emission around 660 nm. Emission from point 3 exhibits an intermediate profile, corresponding to the contribution of both forms. In the case of **AA=O**, a similar tendency is observed with a hydrogen-bond free contribution in the cytoplasm organelles like mitochondria (point 1) and a hydrogen-bonded contribution in the surroundings of the cells (point 2), with emission maxima at 600 and 660 nm, respectively. The emission profiles match again the fluorescence spectra of **AA=O** recorded in acetone and octanol. This dual emission imaging reveals the formation (or not) of hydrogen bonds *in cellulo* and suggests that keto-heptamethines can be considered as useful tools for probing the protic environment in cells or other biological media.

### *In vivo* two-photon microscopy

Finally, the two highly water-soluble keto-derivatives (**D^5^D^5^ =O** and **DD^PHEA^ =O**) were investigated in the context of intravital two-photon microscopy imaging of mice cerebral vascular networks. As these dyes exhibit both good solubility in water and high two-photon brightness in the biological transparency window, they could be potential candidates for in-depth NIR-to-NIR microscopy imaging.

**Fig. 11:**
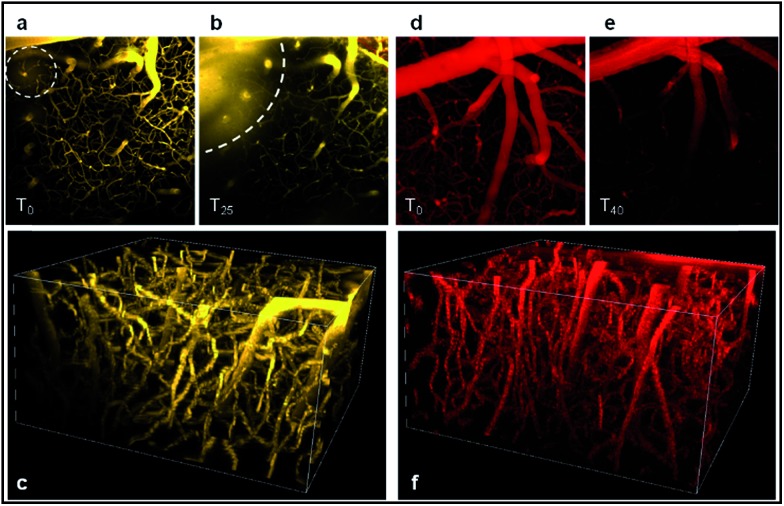
Two-photon fluorescence imaging of mouse cerebral vasculature with **D^5^D^5^ =O** (a–c) and **DD^PHEA^ =O** (d–f) after iv injection. (a, b, d and e) *Z* stack projections of images acquired between 0 and 300 µm deep, at *t* = 0 (a, d), *t* = 25 min (b) and *t* = 40 min (e). (c) and (f) show a 3D reconstruction (*t* = 0) of the brain vasculature with **D^5^D^5^ =O** and **DD^PHEA^ =O** probes, respectively. The difference in color between both was arbitrarily imposed for understandability, and does not correspond to differences in the emission wavelength.

As illustrated in Fig. 11, both probes stain the blood plasma with strong two-photon excitation efficiency at 900 nm. The images obtained were highly contrasted and allowed performing a deep brain vasculature imaging, up to depths of 500 µm. However the staining was not persistent for a long period. In the case of **D^5^D^5^ =O**, the diffusion of the dye across the blood brain barrier (BBB) occurred in the first five minutes. Blurry spots appeared on images (circle on Fig. 11a). Fig. 11b shows the diffusion of the dye within the brain tissue (dotted curved line). This leakage phenomenon increased over time as illustrated by the larger blurred area after 25 min (Fig. 11b). It might be due to a photothermic effect, related to the large absorption coefficient of the molecule and to non-radiative dissipation of the absorbed energy, which can damage the vascular endothelium inducing micro-hemorrhage. In the case of **DD^PHEA^ =O**, which features large water-solubilizing polymeric chains, the release of the dye through blood vessels was not observed (Fig. 11d), yet the cerebral vasculature staining rapidly decreased over time.

**Fig. 12:**
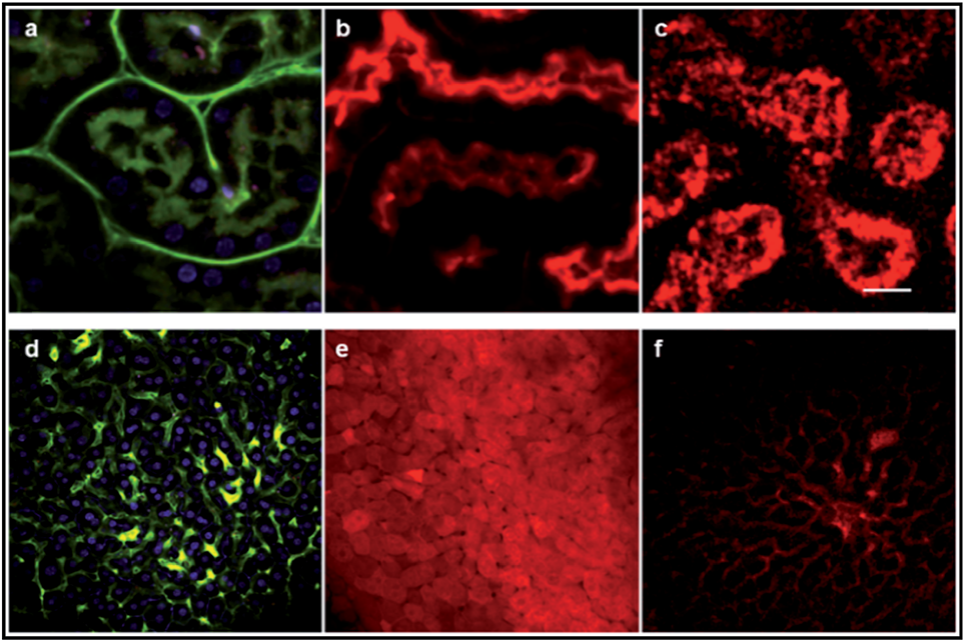
Two-photon microscopy images of the mouse kidney (a–c) and liver (d–f) *ex vivo*. Images (a) and (d) show mouse kidney tubules and hepatocytes, respectively after Hoechst (nucleus) and FITC-dextran (vasculature) iv injection in a control mouse. **D^5^D^5^ =O** circulates in kidney tubules (b) and accumulates in hepatocytes (e) whereas **DD^PHEA^ =O** makes aggregates in kidney tubules epithelial cells (c) and still circulates in the liver vasculature (f).

As shown in Fig. 11e, the image of the cerebral microvasculature becomes almost unresolvable 40 min after intra-venous injection. This difference in the probe behavior can be attributed to their difference in clearance mechanism. While the PHEA appended probe seems to be preferentially metabolized by the kidneys where it makes aggregates in tubule epithelial cells (Fig. 12c), **D^5^D^5^ =O** still circulates in the kidney tubules 2 hours after intravenous injection (Fig. 12b). In contrast, the **D^5^D^5^ =O** derivative stains hepatocytes (Fig. 12e) whereas the PHEA probe keeps circulating in the liver vasculature (Fig. 12f). These results indicate that these two chromophores featuring identical conjugated skeletons behave very differently *in vivo*. The influence of the pendant water-solubilizing groups thus turns out to be crucial regarding the probe properties. Sulfonate containing compounds diffuse through the blood vessels and tend to be more toxic due to liver accumulation. In contrast, the toxicity of water-soluble polymer containing dyes appears to be lower and no diffusion is observed. For these reasons, it seems that **DD^PHEA^ =O** is the best suited for *in vivo* applications in spite of its quite rapid excretion.

## Conclusions

This article constitutes the first systematic evaluation of the potential of keto-polymethine dyes for fluorescence bio-imaging by confocal and two-photon microscopies. Its scope is not limited to the classical **D*^i^* D*^j^* =O** derivatives, as our study involved original **D*^i^* A=O** and **AA=O** analogous species. Detailed spectroscopic investigations clearly demonstrated that this class of dyes provides a valuable alternative to some of the most efficient one- and two-photon fluorophores known to date, including state-of-the-art heptamethines. Regarding confocal microscopy, their large quantum yield in the red-NIR region and their enhanced Stokes shift are substantial advantages over their parent polymethine dyes. For two-photon microscopy, regardless of their symmetry and the nature of their end groups, all keto-heptamethines possess strong two-photon absorption in the 900–950 nm range, which makes them ideal candidates for NIR-to-NIR imaging applications.

The potential of these dyes in microscopy imaging was thus systematically investigated. We showed that the control of the hydrophilic/hydrophobic balance along with appropriate functionalization of **D*^i^* D*^j^* =O** and **D*^i^* A=O** regulates the live cell internalization and localization. Finally, highly hydrosoluble dyes were successfully used for *in vivo* two-photon microscopy of mouse brain vascular networks.

## Experimental section

### Linear and nonlinear optical measurements

UV-visible-NIR absorption spectra were recorded on a Jasco® V-670 spectrophotometer in spectrophotometric grade solvents (*ca.* 10^–5^ mol L^–1^). Molar extinction coefficients (*ε*) were precisely determined at least two times. The luminescence spectra were measured using a Horiba-Jobin Yvon Fluorolog-3® spectrofluorimeter, equipped with a three slit double grating excitation and emission monochromator with dispersions of 2.1 nm mm^–1^ (1200 grooves per mm). The steady-state luminescence was excited by unpolarized light from a 450 W xenon CW lamp and detected at an angle of 90° for diluted solution measurements (10 mm quartz cuvette) by a red-sensitive Hamamatsu R928 photomultiplier tube. Spectra were reference corrected for both the excitation source light intensity variation (lamp and grating) and the emission spectral response (detector and grating). Fluorescence quantum yields *Q* were measured in diluted solution with an optical density lower than 0.1 using the following equation *Q*_x_/*Q*_r_ = [*A*_r_(*λ*)/*A*_x_(*λ*)][*n*_x_^2^/*n*_r_^2^][*D*_x_/*D*_r_] where *A* is the absorbance at the excitation wavelength (*λ*), *n* the refractive index and *D* the integrated intensity. “r” and “x” stand for reference and sample. Excitation of reference and sample compounds was performed at the same wavelength. Short luminescence decay was monitored with the TC-SPC Horiba apparatus using Ludox in distilled water to determine the instrumental response function used for deconvolution. Excitation was performed using NanoLEDs, with models (peak wavelength; pulse duration) 495 (490 nm; <250 ps) and 740 (732 nm; 1.3 ns). The deconvolution was performed using the DAS6 fluorescence-decay analysis software. TPA spectra of keto-heptamethines were mapped with a TPEF setup and the TPA cross sections were characterized with an open-aperture *z*-scan technique. In both the TPEF and *z*-scan techniques, an amplified, femtosecond-pulsed Ti:sapphire laser (Solstice, Spectra-Physics, USA) was used. For TPEF measurements, laser excitation wavelengths were ranging from 760–1300 nm. Rhodamine 6G and LDS821 were used as fluorescence standards. Quartz cuvettes with 1 cm pathlength were used and the optical densities of solutions were <1. Due to the poor stability of LDS821, TPA cross sections of keto-heptamethines were measured with an open-aperture *z*-scan technique and the TPA spectra were calibrated accordingly. For *z*-scan measurements, a near Gaussian beam at 900 and 950 nm with *M* ^2^ ∼ 1.15, beam waist *ω*(HW_1/e2_) ∼ 40 µm, and pulse width *τ*_p_(HW_1/e_) ∼ 100 fs was used. The excitation irradiance ranges from 80–360 GW cm^–2^. The optical pathlength of sample cuvettes for *z*-scan measurements was 1 mm.

### Simulations details

Molecular dynamics simulations were performed using the self-consistent-charge density-functional based tight-binding method (SCC-DFTB), an approximated DFT scheme whose computational efficiency relies on the use of a minimal valence basis-set and parameterized integrals.^23^ In the present work, we used the mio-set^23^ for Slater–Koster tables for methanol based calculations and the halorg parameters to describe the dichloromethane based calculations.^24^ To this end, we used an empirical dispersion term and a correction for the Coulomb interaction in which the Mulliken charges are replaced by the Class IV – Charge Model 3 developed in the context of DFT^25^ and later introduced in the SCC-DFTB potential.^26^ The importance of including such terms to accurately describe molecular aggregates was demonstrated by Simon and co-workers, in particular, these authors showed that they lead to accurate results for water molecule aggregates.^27^ Jahangiri *et al.* also showed that the SCC-DFTB model and its extensions provide reliable results for the structure, energetics, charge distributions, and vibrational spectral of various hydrogen-bonded systems.^28^ Details on the parameterization of the potential is provided in ESI.† In the present work, we used the mio-set for Slater–Koster tables and the halorg parameters to describe the halogen atoms.^24^ The SCC-DFTB approach requires additional corrections to properly deal with weak intermolecular interactions.^26^ To this end, we used an empirical dispersion term^29^ and a correction for the Coulomb interaction in which the Mulliken charges are replaced by the class IV – Charge Model 3 developed in the context of DFT^25^ and later introduced in the SCC-DFTB potential.^30^ In all our calculations, the self-consistent process is stopped when the largest atomic charge fluctuation is smaller than 10^–8^ a.u. All the SCC-DFTB calculations were performed with the deMonNano code.^31^

We performed MD simulations of the simple symmetrical keto-polymethine molecule, *i.e.*, **DD^Me^ =O** where R^i^ = CH_3_ into two different solvents namely methanol and dichloromethane. The simulations consisted of a unique molecule in a 25.0 Å cubic box. To obtain a density that is close to the ambient temperature and pressure density of the systems, 213 and 134 molecules of methanol and dichloromethane were included in the simulation cells. All the simulations were performed in the canonical ensemble. The systems were equilibrated during 20 ps at 300 K using a Berendsen thermostat^32^ following by 20 ps using a Nosé–Hoover chain thermostat^33^ defined by a thermostat frequency and a number of thermostats in the chain of 800 cm^–1^ and 5, respectively. We then performed 80 ps of production run using the Nose–Hoover chain thermostat. In all calculations, we used a time step of 0.5 fs.

### Cell culturing and treatment

We used the T24 human epithelial bladder cancer cell line (ATCC No. HBT-4). In our experiments, T24 cells were cultured in 25 cm^2^ tissue-culture flasks (T25) at 37 °C, in a humidified atmosphere with 5% CO_2_. They were incubated in RPMI 1640 supplemented with 100 U mL^–1^ penicillin, 100 µg mL^–1^ streptomycin, and 10% fetal calf serum (complete medium). Cells were grown to near confluence in the culture flasks and then suspended with 0.05% trypsin–EDTA solution (Sigma). Twenty-four hours before experiments, cells were placed on a LabTek I chambered cover glass (Nunc) at low cell density in complete culture medium. Living T24 cells were incubated for 30 min with solutions of the dyes in water or water/DMSO (98/2) resulting in an overall chromophore concentration in the medium of *ca.* 1 to 2 × 10^–5^ M and imaged without rinsing.

### Confocal microscopy

All confocal experiments were performed using a LSM710 NLO (Carl Zeiss) confocal laser scanning microscope based on an inverted motorized stand (AxioObserver, Zeiss). The excitation was provided by a 561 nm DPSS cw laser in descanned detection mode. In the former case the pinhole was closed to 1 Airy Unit and in the latter one it was fully open. Spectral imaging was realized using an internal Quasar detector in the range 577–723 nm with the resolution of 9.7 nm.

### *In vivo* two-photon microscopy

In accordance with the policy of Grenoble Institute of Neuroscience (GIN) and French legislation, experiments were done in compliance with the European Community Council Directive of November 24, 1986 (86/609/EEC). The research involving animals was authorized by the *Direction Départementale des Services Vétérinaires de l'Isère* – *Ministère de l'Agriculture et de la Pêche, France* and the *Direction Départementale de la protection des populations* – *Préfecture de l'Isère-France* (F. Appaix, PhD, permit number 38 09 39). All efforts were made to minimize the number of mice used and their suffering during the experimental procedure. CD1 mice were housed in cages with food and water *ad libitum* in a 12 h light/dark cycle at 22 ± 1 °C. For *in vivo* two-photon microscopy, 4 months old CD1 mice (*n* = 4) were anesthetized using isoflurane (5% for induction and 1–2% during experiments) in a 70% air, 30% O_2_ gas mixture. Their body temperature was monitored with a rectal probe and maintained at 36 °C using a heating blanket. A MouseOx system (STARR Life Sciences Corp.) for monitoring arterial O_2_ saturation, heart and breath rate was used.

A catheter (Neoflon™, BD, USA) was inserted in the tail vein for an intravenous (iv) injection of 0.1 mL keto-heptamethines (**D^5^D^5^ =O** and **DD^PHEA^ =O**) (5 mg mL^–1^) in saline just before the imaging experiments. A mixture of Hoechst 34580 and Fluorescein IsoThioCyanate-dextran (FITC-dextran, 70 kDa) was injected 2 h after the first iv injection (0.1 mL) to visualize both the vasculature and the nuclei of the two main organs of elimination. Ten minutes afterward, one kidney and the liver were removed for *ex vivo* imaging.

For intravital two-photon imaging of the cerebral vasculature, a craniotomy of 2–3 mm in diameter was performed with a dental drill above the motor cortex and filled with ultrasound gel. In some experiments, the skull was thinned instead of a craniotomy using the same drill. The head was fixed in a homebuilt stereotactic frame.

For two-photon microscopy of *ex vivo* organs, the removed liver and kidney were put in a Petri dish and covered with ultrasound gel.

Two-photon microscopy was performed using a LSM 7MP (Zeiss, Germany) equipped with a 20× water-immersion objective (NA 1.0; Zeiss) and ZEN 2010 software. Laser excitation at 950 nm was done using a Ti:sapphire laser (Chameleon Vision II; Coherent, UK). All the TPM images were obtained using a constant laser power around 60 mW. Fluorescence emissions were detected simultaneously by three non-descanned detectors with a 492/SP25 nm filter (Semrock, USA) for “blue” fluorescence emission, a 542/50 nm filter (Semrock, USA) for “green” fluorescence emission and a 617/73 nm filter (Semrock, USA) for “red” fluorescence emission. Most 3D two-photon microscopic images were acquired as *z*-stacks with a 2 µm step size between each focus plane. The *z*-projections were performed with ImageJ software^34^ and Vaa3D software was used for 3D images reconstruction.^35^
