# Supplementary material for: Keto-polymethines: a versatile class of dyes with outstanding spectroscopic properties for in cellulo and in vivo two-photon microscopy imaging
Source: Chem Sci. 2016 Aug 3;8(1):381–94. doi: 10.1039/c6sc02488b (PMC5365052; doi:10.1039/c6sc02488b)
Supplement: Supplementary file 1 [file SC-008-c6sc02488b-s001.pdf]

Supporting information

Keto-Polymethines: a Versatile Class of Dyes with Outstanding Spectroscopic Properties for In Cellulo and In Vivo Two-Photon Microscopy Imaging

Simon Pascal,[a] Sandrine Denis-Quanquin,[a] Florence Appaix,[b] Alain Duperray,[c,d] Alexei Grichine,[c,d] Boris Le Guennic,[e] Denis Jacquemin,[f,g] Jérôme Cuny,[h] San-Hui Chi,[i] Joseph W. Perry,[i] Boudewijn van der Sanden,[b] Cyrille Monnereau,[a] Chantal Andraud*[,a] and Olivier Maury*[,a]

[a]ENS Lyon, Université de Lyon 1, CNRS Laboratoire de chimie de l'ENS Lyon, UMR 5182 CNRS, 46 allée d'Italie 69364 Lyon, France.

[b]Inserm, Grenoble Institute of Neuroscience, U1216 F-38000 Grenoble, France. Université Grenoble Alpes, GIN, F-38000 Grenoble, France

[c]Inserm, Institut Albert Bonniot, U823, F-38000 Grenoble, France

[d]Université Grenoble Alpes, IAB, F-38000 Grenoble, France

[e]Institut des Sciences Chimiques de Rennes, UMR 6226 CNRS, Université de Rennes 1, 263 Avenue du Général Leclerc, 35042 Rennes Cedex, France

[f]Laboratoire CEISAM, CNRS 6230, Université de Nantes, 2 Rue de la Houssinière, BP 92208, 44322 Nantes Cedex 3, France

[g]Institut Universitaire de France, 103 Bvd Michelet, 75005 Paris Cedex 5, France

[h]Laboratoire de Chimie et Physique Quantiques (LCPQ), Université de Toulouse III [UPS] and CNRS, 118 Route de Narbonne, 31062 Toulouse, France

[i]School of Chemistry and Biochemistry, Center for Organic Photonics and Electronics, Georgia Institute of Technology, 901 Atlantic Drive NW, Atlanta, GA 30332-0400, USA

Synthesis. NMR spectra (^{1}H, ^{13}C) were recorded on a BRUKER® Advance operating at 500.10 MHZ and 125.75 MHz for ^{1}H and ^{13}C, respectively. ^{13}C NMR spectra were recorded using the µDEFT experiment.[1] Data are listed in parts per million (ppm) and are reported relative to residual solvent peaks being used as internal standard (^{1}H ($CDCl_3$): 7.26 ppm, ^{13}C ($CDCl_3$): 77.2 ppm; ^{1}H (DMSO-d6): 2.50 ppm, ^{13}C (DMSO-d6): 39.5 ppm; ^{1}H (CD_3CN): 1.94 ppm). High resolution mass spectrometry measurements were performed at Centre Commun de Spectrometrie de Masse (Villeurbanne, France) by ESI-TOF: a Bruker Daltonics ® Micro TOF-Q II was used with a resolution of 8000, in positive mode with a capillary tension of 4500V, a source temperature of 180 °C, and a cone tension of 60V. The internal reference used for calibration was sodium formate. GPC/SEC measurement was performed at Centre for the Characterization of Polymers by Liquid Chromatography of the Institut de Chimie de Lyon using a Viscotek apparatus with the following configuration: Rhéodyne 7725i injector, three Styragel HR 4E columns (WAT0044241) at 70 °C and RI-55 °C (Viskotek VE3580) and viscosimeter (Viskotek T60A) detectors. Starting materials were purchased from Sigma Aldrich®, Acros Organics® or Alfa Aesar® with the best available quality grade. All reactions were routinely performed under argon atmosphere in anhydrous solvents. Column chromatography were performed using Acros Organics® (0.035-0.070 mm) silicagel or neutral aluminium oxide (50-200 µm, 60 A). All reagents were purchased from commercial sources and used without further purification, otherwise noted. **1**, **2**, **D^1**, **D^{3}A**,[2] **D^5**,[3] **D^3**,[4] **D^4**,[5] and **AA**[6] were prepared according to published procedures.

DOSY characterization for the polymer. 1D-1H and 2D-DOSY experiments were carried out at 298 K on a 500 MHz BRUKER® Avance NMR spectrometer employing a 5 mm TXI probe equipped with z-gradients. 2D-DOSY experiments were acquired using a LED experiment with bipolar pulses. Gradients were linearly sampled in 50 points. 16 scans were acquired on 8192 data points. The gradient pulse length was d/2 = 1.5 ms and the D diffusion delay were adapted to the sample for values in the 150-250 ms range. The DOSY spectra were obtained by applying an Inverse Laplace Transform (ILT) along the diffusion axis, using the Gifa algorithm embedded into the commercial software NMR notebook (NMRTEC, Illkirch). Careful spline polynomial correction was applied along the F2 dimension before the ILT processing. PDI was then calculated from the ratio of diffusion coefficients D_w/D_n measured from DOSY signals, D_w corresponding to the average diffusion coefficient of the polymer main chain, while D_n corresponds to the average diffusion coefficient of the chromophoric extremities.

Titrations. Plot of the spectroscopic data obtained upon titration of each keto-chromophore in solution with methanol aliquots was done using the following general methodology: to a known volume (3 mL) of a solution of the keto-heptamethine in dichloromethane (**D^{1}D^1=O** and **D^{2}A=O**) or acetonitrile (**AA=O**) of identical concentrations (2.7 10^{-6} M) were progressively added aliquots of methanol. At each point of the titration the total concentration of keto-chromophore, C_0 (mol.L^{-1}) is given by

$$C_0 = C_{in} \times \frac{V_0}{(V_0 + V_a)}$$

With C_i : initial concentration of the keto-chromophore's solution (mol.L^{-1}), V_0 : initial volume of the solution (L), V_a : added methanol volume (L)

Similarly it can be shown that the total concentration in methanol $_S$ (mol.L^{-1}) $_S$ given by

$$S = \frac{\rho_{MeOH}}{M_{MeOH}} \times \frac{V_a}{(V_0 + V_a)}$$

With ρ_{MeOH} : the density of methanol (g.L^{-1}) and M_{MeOH}: the molar mass of methanol (g.mol^{1})

By following the decrease in the emission intensity originating from the unbound keto-derivative, it is possible to calculate the concentration of the hydrogen-bonded complex [C-S] using the formula

$$[C-S] = Co \times \frac{(I_o - I)}{I_0}$$

Where I is the measured fluorescence intensity, and I_0 the „dilution corrected“ initial emission intensity of the pure non-bonded form, which is related to the initial emission intensity by:

$$I_0 = I_{in} \times \frac{Co}{C_{in}}$$

From the [C-S] concentration, it is possible to calculate [C] and [S], concentration fort the unbound keto species and unbound methanol, respectively, following

$$[C] = Co - [C-S]$$

$$[S] = So - [C-S]$$

Assuming a 1:1 binding isotherm, the binding constant K_a can be expressed as follows:

$$K_a = \frac{[C-S]}{[C]x[S]}$$

Or alternatively

$$[C]\text{x}[S]K_a = [C-S]$$

Upon plotting the evolution of [C]x[S] *vs* the product [C-S], a straight line should be obtained in case of a 1:1 association, which slope corresponds to K_a.

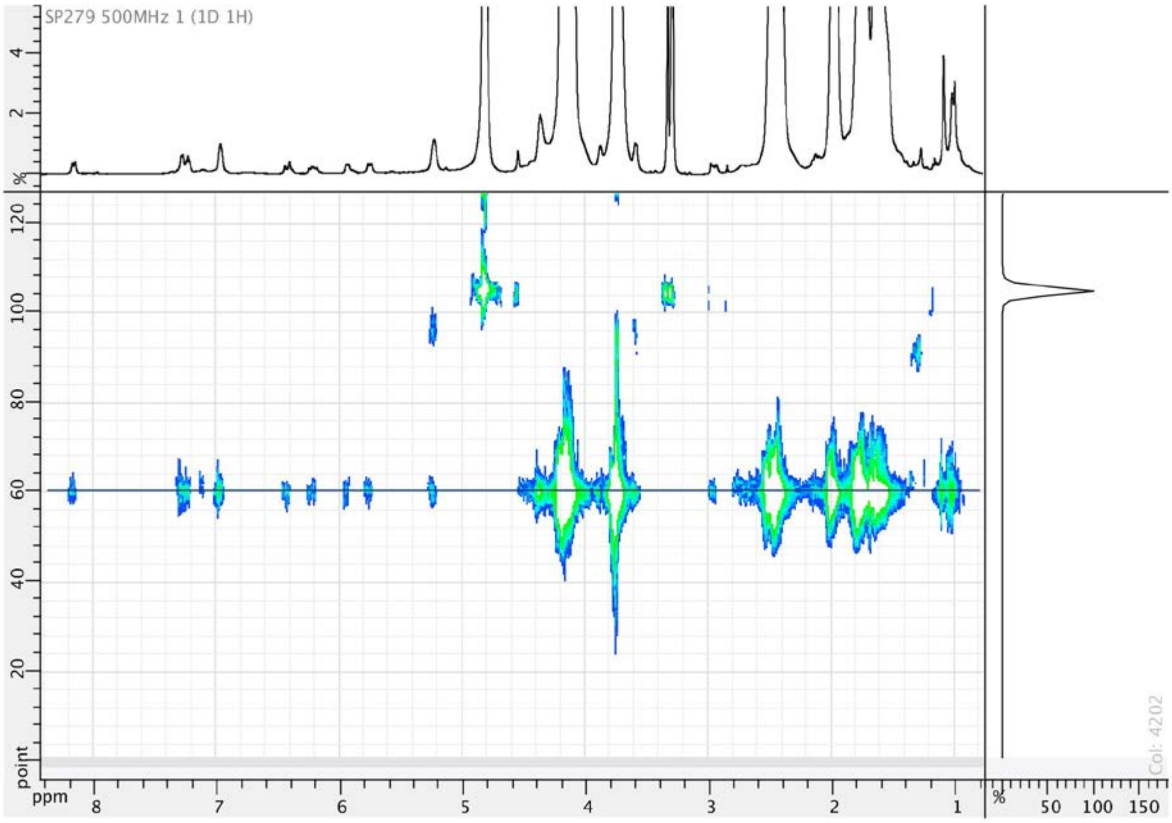

Figure S1. 2D DOSY NMR of **DD$^{\mathbf{PHEA}}$=O** in CD_3OD

Universal Calibration - Homopolymers : Results

Peak RV - (ml)	19,870
Mn - (Daltons)	32 655
Mw - (Daltons)	43 388
Mz - (Daltons)	57 344
Mp - (Daltons)	37 209
Mw / Mn	1,329
Percent Above Mw: 0	0,000
Percent Below Mw: 0	0,000
Mw 10.0% Low	15 508
Mw 10.0% High	100 118
IV - (dl/g)	0,1979
Rh - (nm)	5,010
Wt Fr (Peak)	1,000
Mark-Houwink a	0,636
Mark-Houwink logK	-3,647
Branches	0,000
Branch Freq.	0,000
RI Area - (mvml)	19,45
UV Area - (mvml)	0,00
RALS Area - (mvml)	0,00
LALS Area - (mvml)	0,00
IVDP Area - (mvml)	38,10

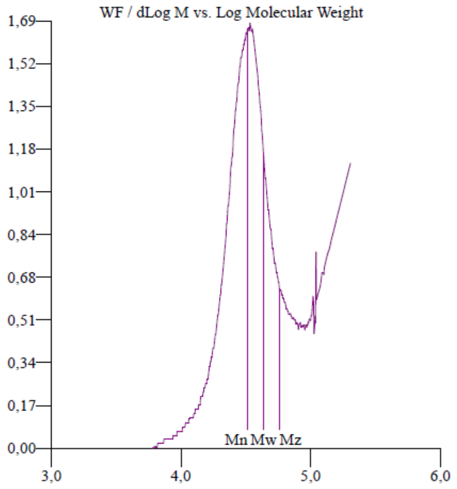

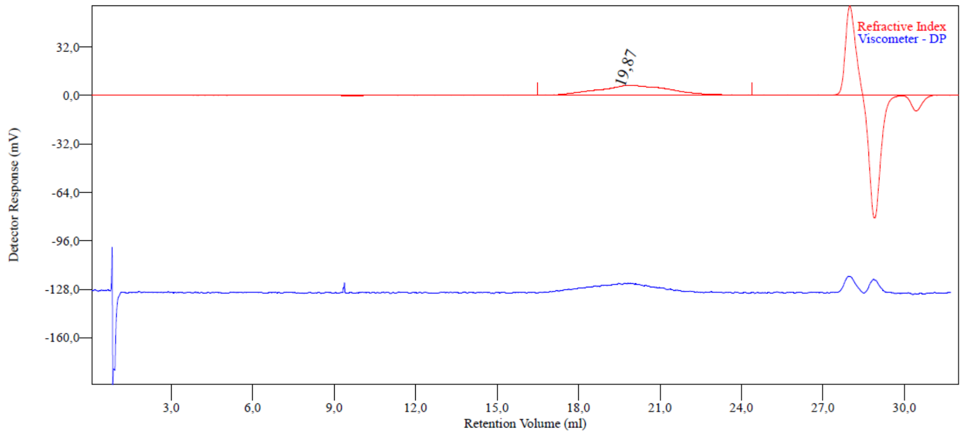

Figure S2. SEC analysis of **DD^{PHEA}=O** in LiBr-DMF (concentration: 3.58 mg.mL^{-1}). Top: with refractive index detection Bottom: with viscosimetry detection. Mn, M_w and PDI determination are based on the data obtained with refractive index detection

Additional simulations details. The parametrization of the SCC-DFTB CM3 charges used in the present work is the same used by Simon and co-workers for OH, *i.e.* D_{OH} = 0.12873. D_{CO} = 0.1 and D_{CH} = 0.0 were used as they well reproduce the M062X-D3 geometries and binding energies of small methanol clusters. For instance, the binding energy of the $(CH_3OH)_2$ dimer is -5.53 $kcal.mol^{-1}$ at the SCC-DFTB level of theory as compared to -5.89 and -6.37 $kcal.mol^{-1}$ at the M062X-D3/aug-cc-pVTZ and B3LYP-D3/QZVP levels of theory, respectively. These latter results were corrected for basis set superposition error (BSSE) and were obtained using the Gaussian 09 package.[7] For chlorine, the D_{CCl} = 0.0 value was used.

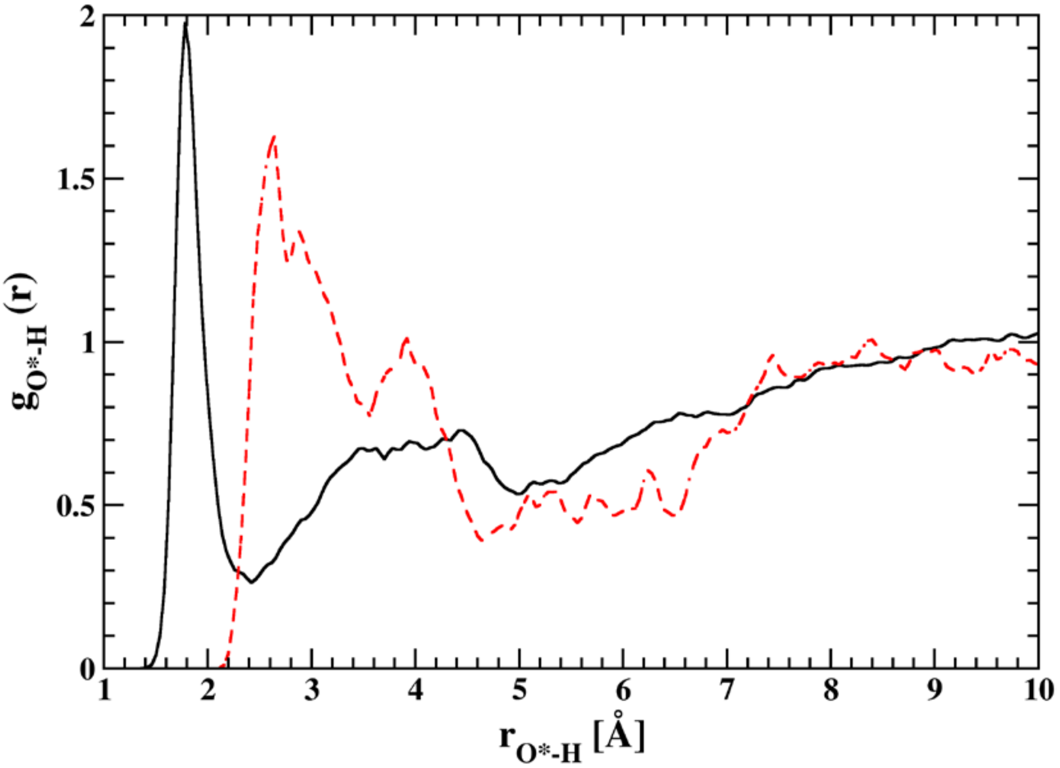

Figure S3. Radial distribution functions for O*-H in methanol (black curve, plain line) and dichloromethane (red curve, dashed line). O* stands for the oxygen atoms of the model **DD^{Me}=O** and H for the hydrogen atoms of the solvent.

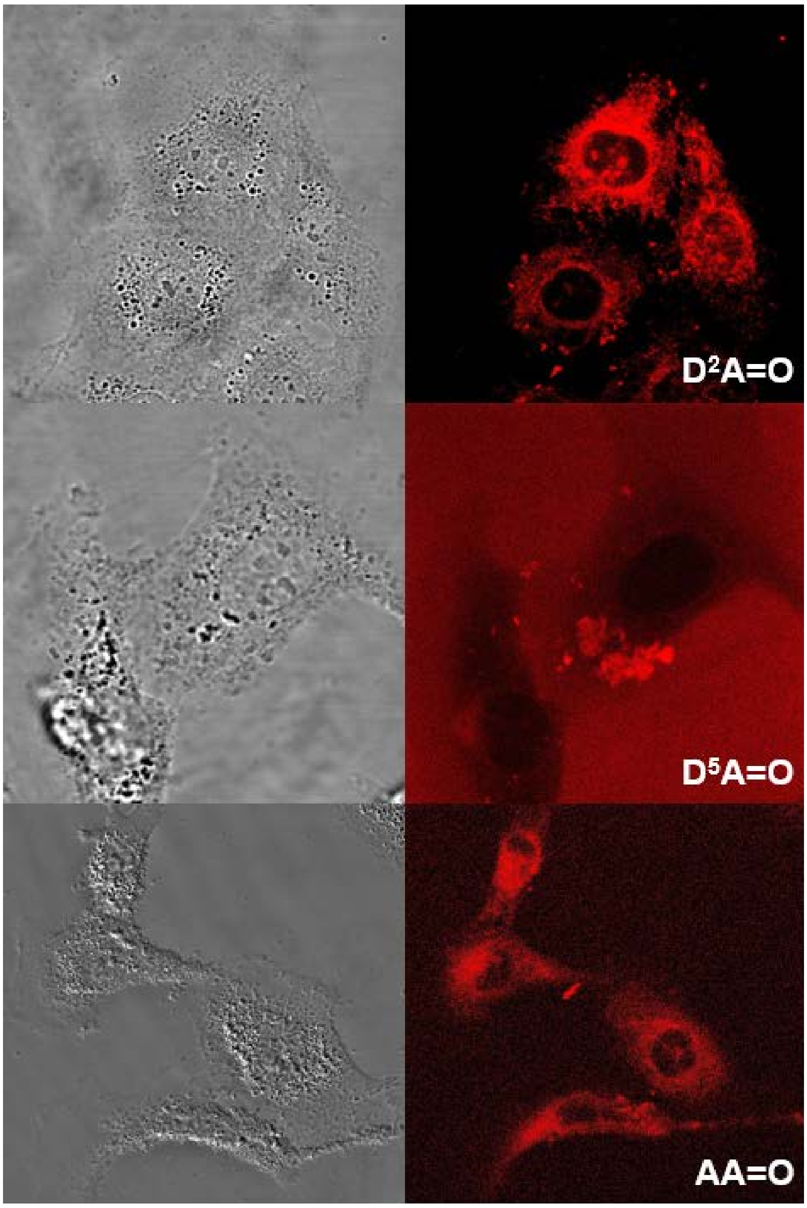

Figure S4. Transmission (left) and two-photon fluorescence (right) microscopy images of T24 cancer cells after their incubation with **D^{2}A=O** (top), **D^{5}A=O** (middle) and **AA=O** (bottom). Two-photon excitation was performed at 950 nm.

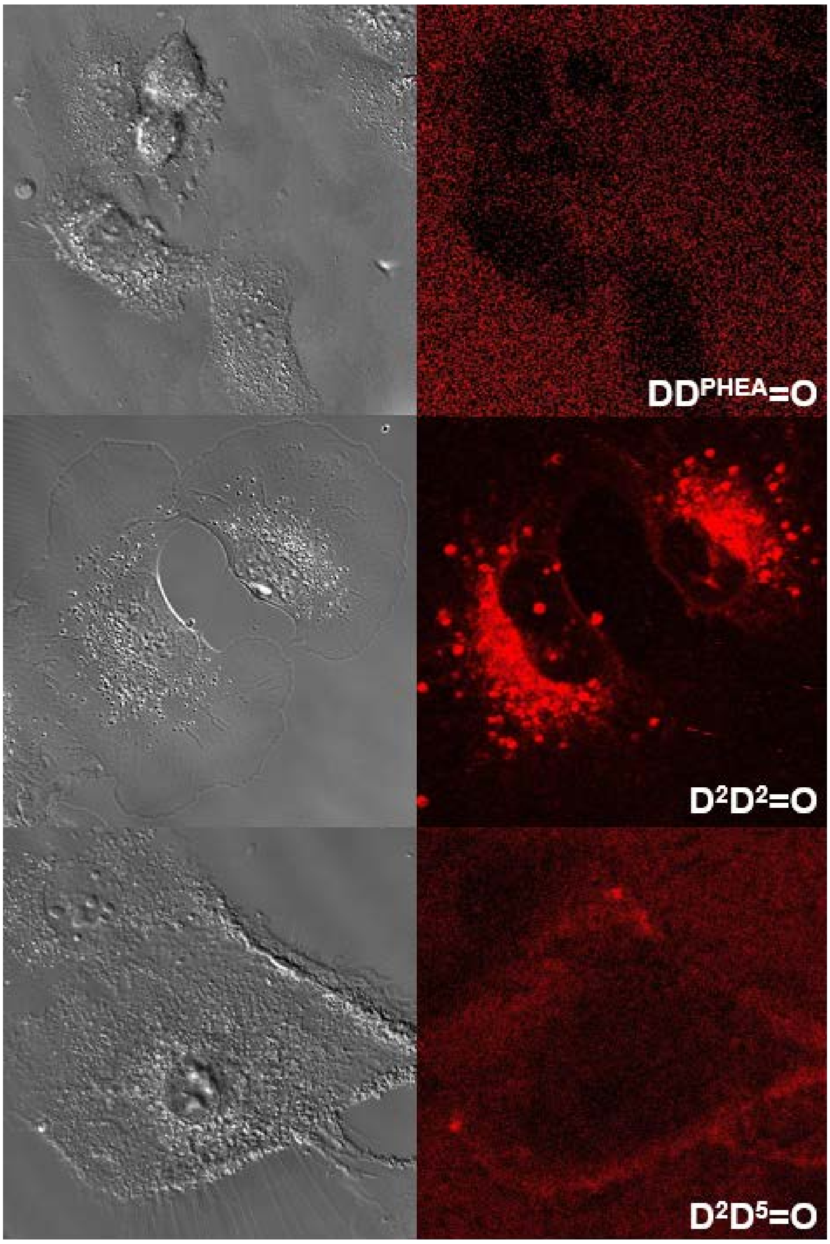

Figure S5. Transmission (left) and fluorescence (right) confocal microscopy images of T24 cancer cells after their incubation with **DD^{PHEA}=O** (top), **D^{2}D^2=O** (middle) and **D^{2}D^5=O** (bottom). One photon excitation was performed at 560 nm.

Compound D^1D^1

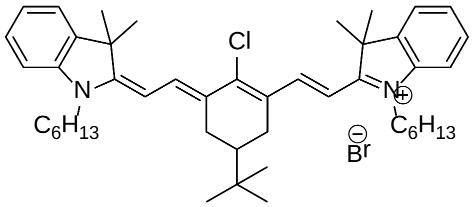

To a solution of 961 mg of **1** (4.20 mmol, 1 equiv.) and 2.86 g of **D^1** (8.82 mmol, 2.1 equiv.) in 60 mL of absolute ethanol was added 0.85 mL of distilled pyridine (10.5 mmol, 2.5 equiv.). The solution was stirred for 15 h at 40 °C. The resulting greenish solution was diluted with 100 mL of dichloromethane (DCM) and washed with a dilute aqueous solution of HCl (50 mL), then water (50 mL) and brine (50 mL). The organic layer was dried over Na_2SO_4 and concentrated. The crude solid was dissolved in a minimal amount of DCM and precipitated by slow addition of pentane to afford the product as a dark greenish solid in 64% yield (2.034 g).

^{1}H NMR ($CDCl_3$, 500.10 MHz): δ 8.35 (d, ^3J = 14 Hz, 2H, =C**H**), 7.40 (m, 4H, C**H**$_{Ar}$), 7.26-7.23 (m, 4H, C**H**$_{Ar}$), 6.17 (d, ^3J = 14 Hz, 2H, =C**H**), 4.19 (m, 4H, N-C**H**$_2$), 2.88 (m, 2H, **H**$_{eq}$), 2.23 (dd, ^2J = 14 Hz, ^3J = 14 Hz, 2H, **H**$_{ax}$), 1.86 (m, 4H, C**H**$_2$), 1.73 (s, 12H, C(C**H**$_3$)$_2$), 1.60 (m, 1H, C**H**), 1.46 (m, 4H, C**H**$_2$), 1.34 (m, 8H, C**H**$_2$), 1.08 (s, 9H, C(C**H**$_3$)$_3$), 0.88 (t, ^3J = 7 Hz, 6H, C**H**$_3$-CH_2).

^{13}C NMR ($CDCl_3$, 125.75 MHz): δ 172.5 (C_{quat}), 150.4 (C_{quat}), 144.3 (CH), 142.3 (C_{quat}), 141.2 (C_{quat}), 129.0 (CH), 127.4 (C_{quat}), 125.6 (CH), 122.4 (CH), 111.2 (CH), 101.1 (CH), 49.6 (C_{quat}), 45.0 (N-CH_2), 42.5 (CH), 32.6 (C_{quat}), 31.5 (CH_2), 28.3 (CH_3), 28.2 (CH_3), 27.7 (CH_2), 27.6 (CH_3), 27.4 (CH_2), 26.8 (CH_2), 22.6 (CH_2), 14.1 (CH_3).

UV-Vis (CH_3OH): λ_{max} = 780 nm (ε_{max} = 257000 $L.mol^{-1}.cm^{-1}$).

HRMS (ESI+): $[M-Br]^+$ = 679.4758 (calcd for $C_{46}H_{64}ClN_2^+$: 679.4753).

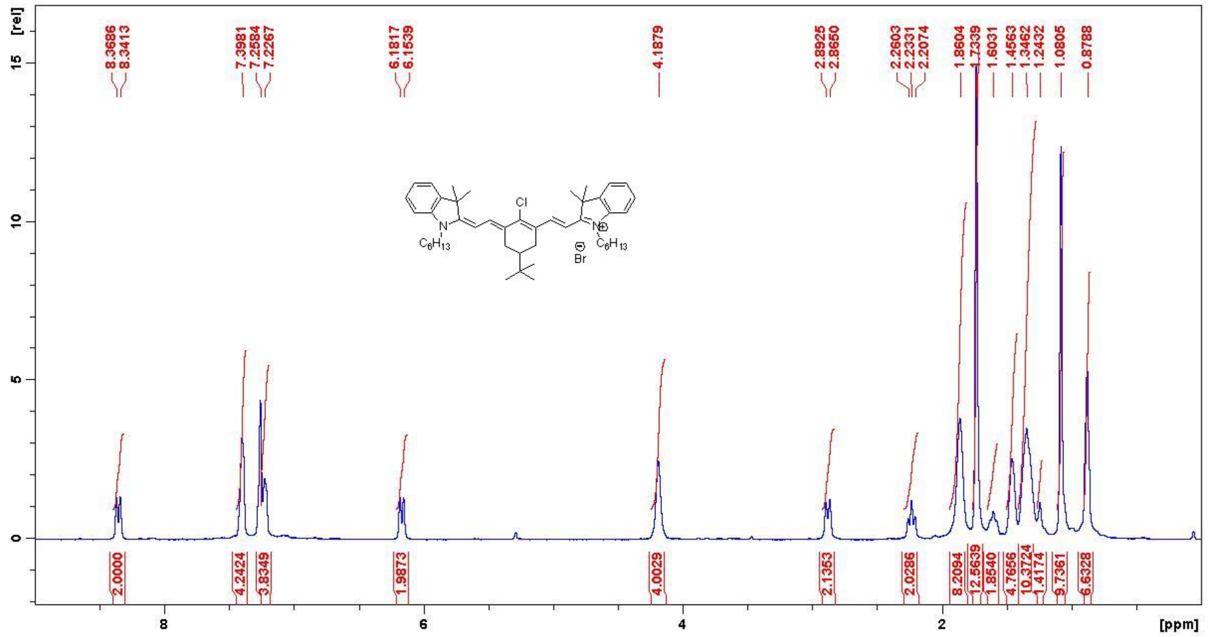

[rel]
[ppm]
8.3686
8.3413
7.3981
7.2584
7.2267
6.1817
6.1539
4.1879
2.8925
2.8650
2.2603
2.2331
2.2074
1.8604
1.7339
1.6031
1.4563
1.3462
1.2432
1.0805
0.8788
2.0000
4.2424
3.8349
1.9873
4.0029
2.1353
2.0286
8.2094
12.5639
1.8540
4.7656
10.3724
1.4174
9.7361
6.6328
Cl
N
C_6H_{13}
Br

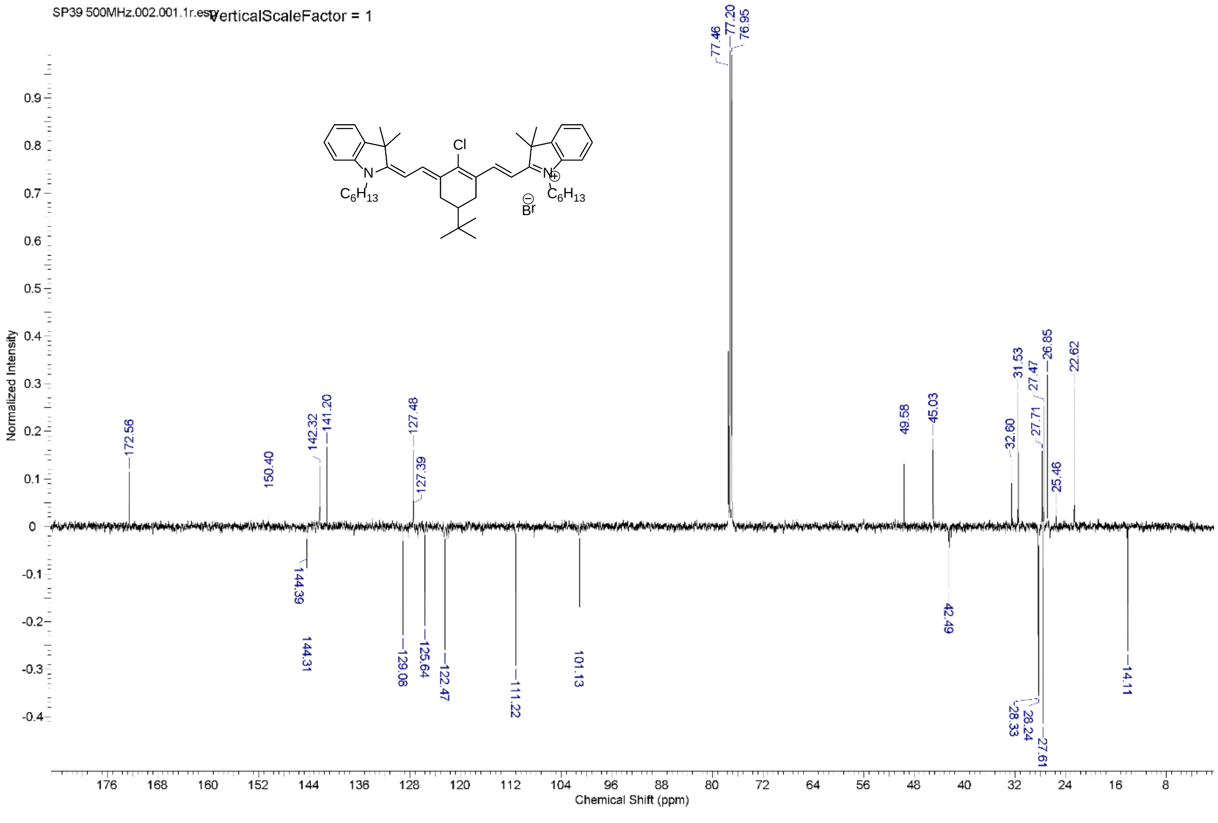

SP39 500MHz.002.001.1r.esp
VerticalScaleFactor = 1
Normalized Intensity
Chemical Shift (ppm)
77.46
77.20
76.95
172.56
150.40
142.32
141.20
127.48
127.39
144.39
144.31
129.06
125.64
122.47
111.22
101.13
49.58
45.03
42.49
32.60
31.53
27.71
27.47
26.85
25.46
22.62
28.33
28.24
27.61
14.11
Cl
N
C_6H_{13}
Br

Compound D^1D^1=O

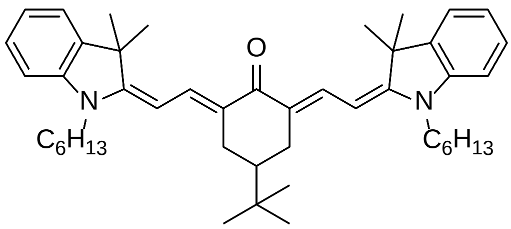

To a solution of 200 mg of **D^1D^1** (0.26 mmol, 1 equiv.) and 61 mg of *N*-hydroxysuccinimide (0.53 mmol, 2 equiv.) in 5 mL of anhydrous DMF were added 90 µL of distilled diisopropylethylamine (DIEA) (0.53 mmol, 2 equiv.) and the mixture was stirred for 3 h at RT. Then the solution was added to 10 mL of DCM and washed with an aqueous saturated solution of NH_4Cl (10 mL) then with water (10 mL). The organic layer was dried over Na_2SO_4 and concentrated. After filtration trough an activated alumina plug (75 g Al_2O_3 with 6% H_2O) and elution with DCM/PE (1:1, R_f = 0.26), the product was isolated as a reddish solid in 72% yield (126 mg).

^{1}H NMR ($CDCl_3$, 500.10 MHz): δ 8.16 (d, ^{3}J = 13 Hz, 2H, =C**H**), 7.26-7.17 (m, 4H, C**H**$_{Ar}$), 6.90 (d, ^{3}J = 7 Hz, 2H, C**H**$_{Ar}$), 6.68 (d, ^{3}J = 8 Hz, 2H, C**H**$_{Ar}$), 5.48 (d, ^{3}J = 13 Hz, 2H, =C**H**), 3.67 (t, ^{3}J = 7 Hz, 4H, N-C**H**$_2$), 2.87 (d, ^{2}J = 13 Hz, 2H, **H**$_{eq}$), 2.13 (dd, ^{2}J = 14 Hz, ^{3}J = 14 Hz, 2H, **H**$_{ax}$), 1.73 (t, ^{3}J = 7 Hz, 4H, C**H**$_2$), 1.68 (s, 6H, C(C**H**$_3$)$_2$), 1.67 (s, 6H, C(C**H**$_3$)$_2$), 1.45-1.32 (m, 13H, C**H**$_2$ and C**H**), 1.06 (s, 9H, C(C**H**$_3$)$_3$), 0.91 (t, ^{3}J = 7 Hz, 6H, C**H**$_3$-CH_2).

^{13}C NMR ($CDCl_3$, 125.75 MHz): δ 186.6 (C_{quat}), 162.4 (C_{quat}), 144.4 (CH), 139.8 (C_{quat}), 141.2 (C_{quat}), 132.9 (CH), 127.7 (CH), 126.6 (C_{quat}), 121.9 (CH), 120.5 (CH), 106.7 (CH), 46.6 (C_{quat}), 43.7 (CH), 42.7 (N-CH_2), 32.7 (C_{quat}), 31.6 (CH_2), 28.9 (CH_3), 28.8 (CH_3), 27.6 (CH_3), 27.0 (CH_2), 26.9 (CH_2), 26.2 (CH_2), 22.7 (CH_2), 14.1 (CH_3).

UV-Vis (CH_3OH): λ_{max} = 532 nm (ε_{max} = 56000 L.mol^{-1}.cm^{-1}).

UV-Vis (CH_3OH + CH_3CO_2H): λ_{max} = 712 nm (ε_{max} = 99000 L.mol^{-1}.cm^{-1}).

HRMS (ESI+): $[M+H]^+$ = 661.5081 (calcd for $C_{46}H_{65}N_2O^+$: 661.5091).

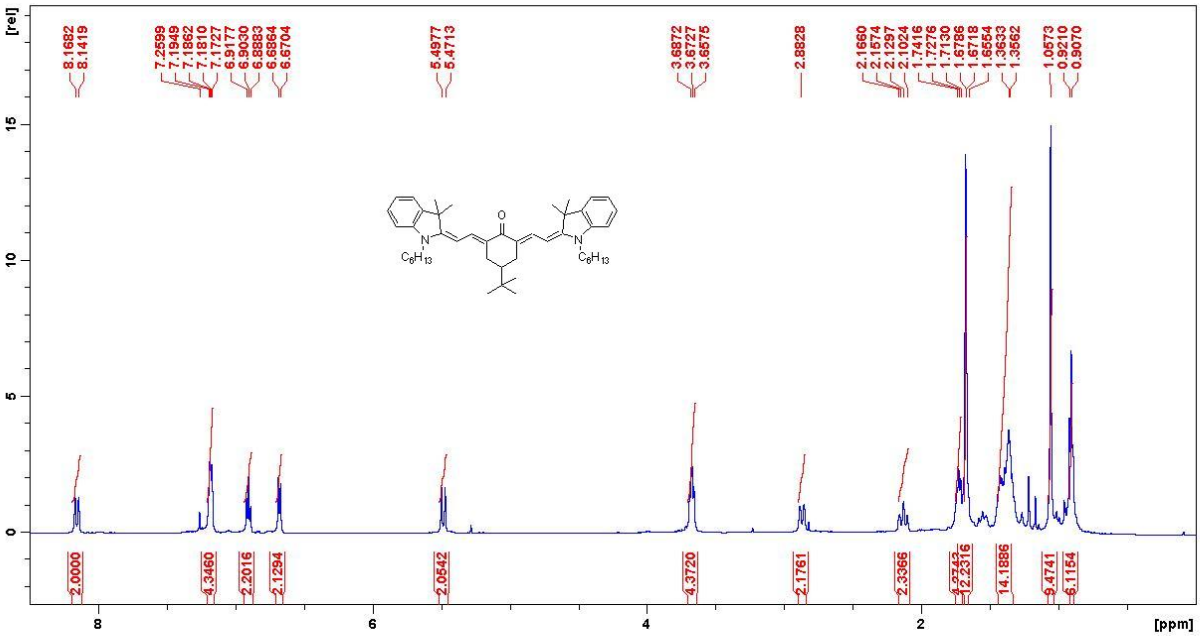
[rel]
8.1682
8.1419
7.2599
7.1949
7.1862
7.1810
7.1727
6.9177
6.9030
6.8883
6.6864
6.6704
5.4977
5.4713
3.6872
3.6727
3.6575
2.8828
2.1660
2.1574
2.1297
2.1024
1.7416
1.7276
1.7130
1.6786
1.6718
1.6554
1.3633
1.3562
1.0573
0.9210
0.9070
2.0000
4.3460
2.2016
2.1294
2.0542
4.3720
2.1761
2.3366
12.2316
14.1886
9.4741
6.1154
[ppm]

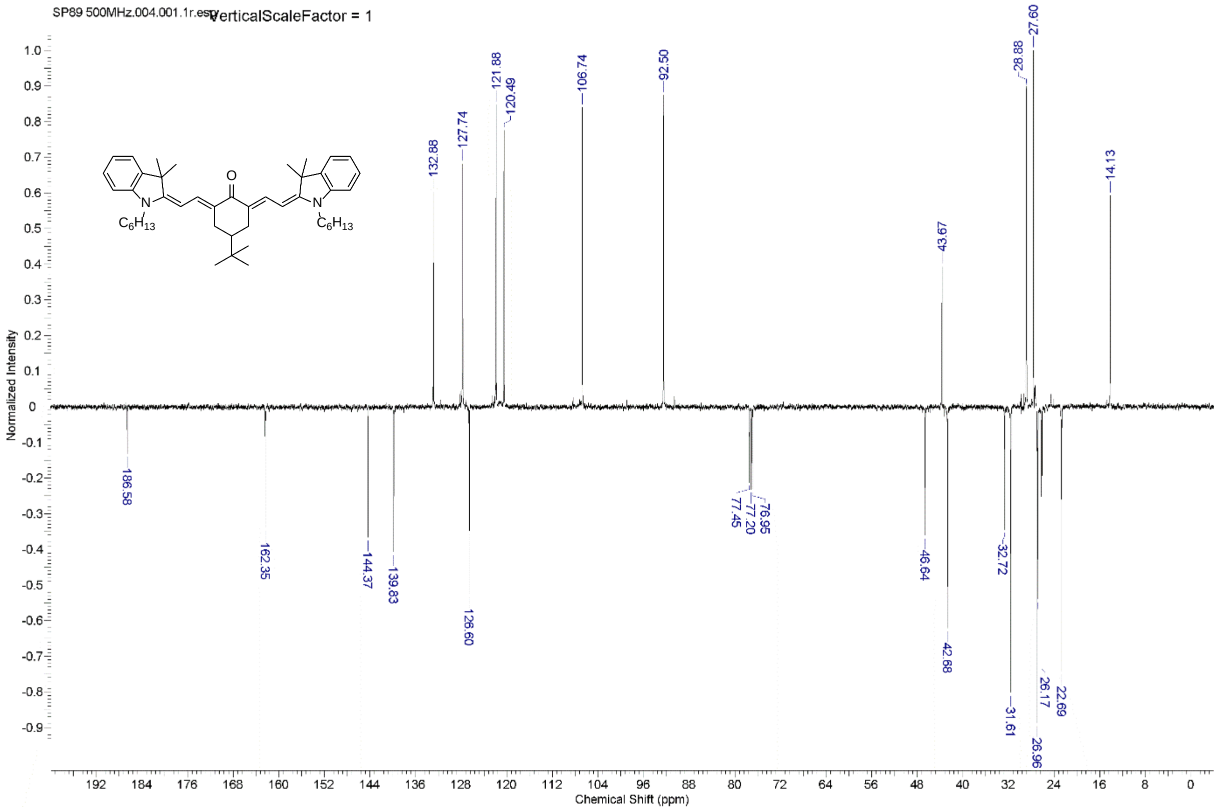
SP89 500MHz.004.001.1r.esp
VerticalScaleFactor = 1
Normalized Intensity
Chemical Shift (ppm)
132.88
127.74
121.88
120.49
106.74
92.50
28.88
27.60
14.13
43.67
186.58
162.35
144.37
139.83
126.60
77.45
77.20
76.95
46.64
42.68
32.72
31.61
26.17
26.96
22.69

Compound D[2]

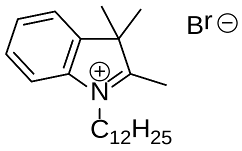

A solution of 6 g of freshly distilled 2,3,3-trimethylindolenine (37.68 mmol, 1 equiv.) and 18.1 mL of 1-bromododecane (75.36 mmol, 2 equiv.) in 30 mL of anhydrous toluene was stirred for 3 days at 110 °C. The purple solution was concentrated and the remaining starting material was removed using dynamic trap-to-trap technique. The obtained dark reddish residue was recrystallized in EtOAc to afford the product as a dark reddish solid in 21% yield (3.253 g).

^{1}H NMR (DMSO-d$_6$, 500.10 MHz): δ 7.98 (m, 1H, C**H**$_{Ar}$), 7.84 (m, 1H, C**H**$_{Ar}$), 7.61 (m, 2H, C**H**$_{Ar}$), 4.45 (t, ^{3}J = 8 Hz, 2H, N-C**H**$_2$), 2.84 (s, 3H, N=C-C**H**$_3$), 1.82 (quint, ^{3}J = 8 Hz, 2H, C**H**$_2$), 1.53 (s, 6H, C(C**H**$_3$)$_2$), 1.40-1.22 (m, 18H, C**H**$_2$), 0.85 (t, ^{3}J = 7 Hz, 3H, CH$_2$-C**H**$_3$).

^{13}C NMR (DMSO-d$_6$, 125.75 MHz): δ 196.4 (C$_{quat}$), 141.9 (C$_{quat}$), 141.1 (C$_{quat}$), 129.4 (CH), 128.9 (CH), 123.5 (CH), 115.5 (CH), 54.1 (**C**(CH$_3$)$_2$), 47.6 (N-CH$_2$), 31.2 (CH$_2$), 29.0 (2 CH$_2$), 28.9 (CH$_2$), 28.8 (CH$_2$), 28.7 (CH$_2$), 28.6 (CH$_2$), 27.2 (CH$_2$), 25.8 (CH$_2$), 22.4 (CH$_2$), 22.0 (C(**C**H$_3$)$_2$), 14.0 (CH$_3$), 13.9 (CH$_3$).

HRMS (ESI+): [M−Br]$^+$ = 328.2998 (calcd for $C_{23}H_{38}N^+$: 328.2999).

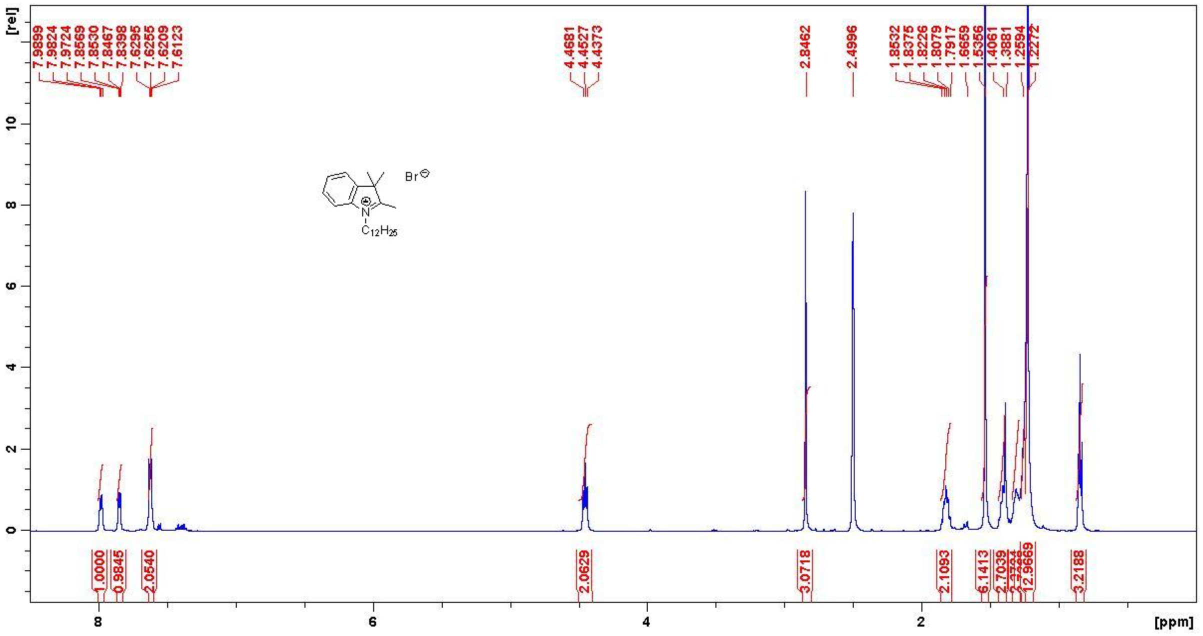

[rel]
7.9899
7.9824
7.9724
7.8569
7.8530
7.8467
7.8398
7.6295
7.6255
7.6209
7.6123
4.4681
4.4527
4.4373
2.8462
2.4996
1.8532
1.8375
1.8226
1.8079
1.7917
1.6659
1.5356
1.4061
1.3881
1.2594
1.2272
Br
C12H25
1.0000
0.9845
2.0540
2.0629
3.0718
2.1093
6.1413
2.7039
12.9669
3.2188
[ppm]

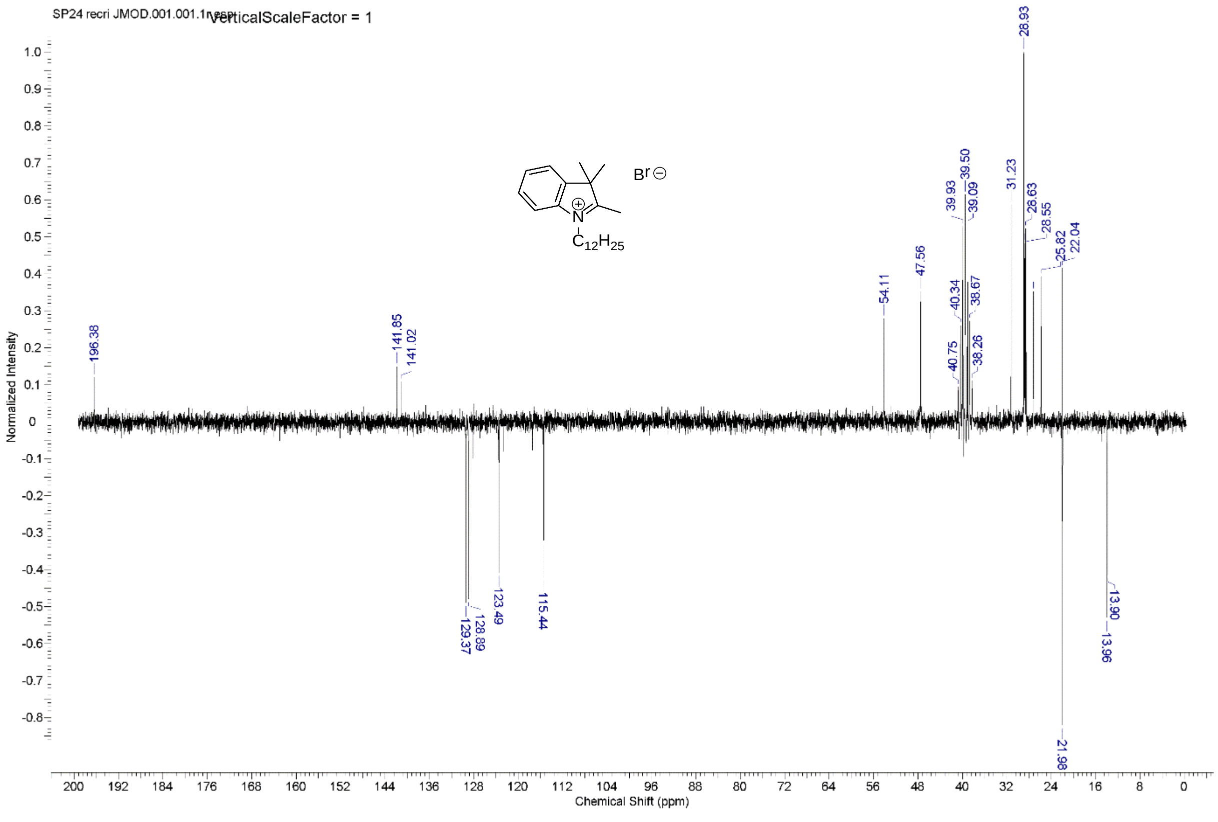

SP24 recri JMOD.001.001.1r.esp
verticalScaleFactor = 1
Normalized Intensity
Chemical Shift (ppm)
196.38
141.85
141.02
129.37
128.89
123.49
115.44
54.11
47.56
40.75
40.34
39.93
39.50
39.09
38.67
38.26
31.23
28.93
28.63
28.55
25.82
22.04
21.98
13.90
13.96
Br
C12H25

Compound D^2D^2

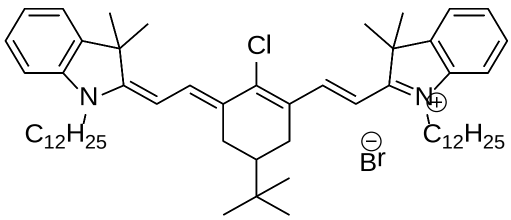

To a solution of 234 mg of **1** (1.02 mmol, 1 equiv.) and 920 mg of $\mathbf{D^2}$ (2.25 mmol, 2.2 equiv.) in 10 mL of absolute ethanol was added 0.21 mL of distilled pyridine (2.56 mmol, 2.5 equiv.). The reaction was stirred for 17 h at 40 °C and then allowed to cool to RT. Ethanol was evaporated and the residue was dissolved in DCM and washed with an aqueous solution of HBr 1M then with water. The organic layer was dried over Na_2SO_4 and concentrated. The crude solid was purified by flash chromatography on silica, using DCM/MeOH as eluent (95:5, R_f = 0.63) to afford the product as a greenish solid in 68% yield (649 mg).

^{1}H NMR ($CDCl_3$, 500.10 MHz): δ 8.34 (d, ^3J = 14 Hz, 2H, =C**H**), 7.42-7.39 (m, 4H, C$\mathbf{H}_{Ar}$), 7.27-7.24 (m, 2H, C$\mathbf{H}_{Ar}$), 7.22 (d, ^3J = 8 Hz, 2H, C$\mathbf{H}_{Ar}$), 6.19 (d, ^3J = 14 Hz, 2H, =C**H**), 4.22 (m, 4H, C$\mathbf{H}_2$), 2.89 (dd, ^2J = 14 Hz, ^3J = 3 Hz, 2H, $\mathbf{H}_{eq}$), 2.23 (dd, ^2J = 14 Hz, ^3J = 14 Hz, 2H, $\mathbf{H}_{ax}$), 1.87 (m, 4H, C$\mathbf{H}_2$), 1.73 (s, 6H, C(C$\mathbf{H}_3$)$_2$), 1.72 (s, 6H, C(C$\mathbf{H}_3$)$_2$), 1.60 (m, 1H, C**H**), 1.47-1.42 (m, 4H, C$\mathbf{H}_2$), 1.40-1.35 (m, 4H, C$\mathbf{H}_2$), 1.28 (m, 28H, C$\mathbf{H}_2$), 1.10 (s, 9H, C(C$\mathbf{H}_3$)$_3$), 0.87 (t, ^3J = 7 Hz, 6H, C$\mathbf{H}_3$-CH_2).

^{13}C NMR ($CDCl_3$, 125.75 MHz): δ 172.5 (C_{quat}), 150.1 (C_{quat}), 144.1 (CH), 142.4 (C_{quat}), 141.2 (C_{quat}), 129.0 (CH), 127.5 (C_{quat}), 125.5 (CH), 122.4 (CH), 111.2 (CH), 101.3 (CH), 49.5 (C_{quat}), 45.0 (N-CH_2), 42.5 (CH), 32.6 (C_{quat}), 32.0 (CH_2), 29.7 (2 CH_2), 29.6 (2 CH_2), 29.4 (2 CH_2), 28.3 (CH_3), 28.2 (CH_3), 27.7 (CH_2), 27.6 (CH_3), 27.6 (CH_2), 27.2 (CH_2), 22.8 (CH_2), 14.3 (CH_3).

UV-Vis (CH_3OH): λ_{max} = 782 nm (ε_{max} = 266000 L.mol^{-1}.cm^{-1}).

HRMS (ESI+): [M−Br]$^+$ = 874.6606 (calcd for $C_{58}H_{88}ClN_2^+$: 847.6631).

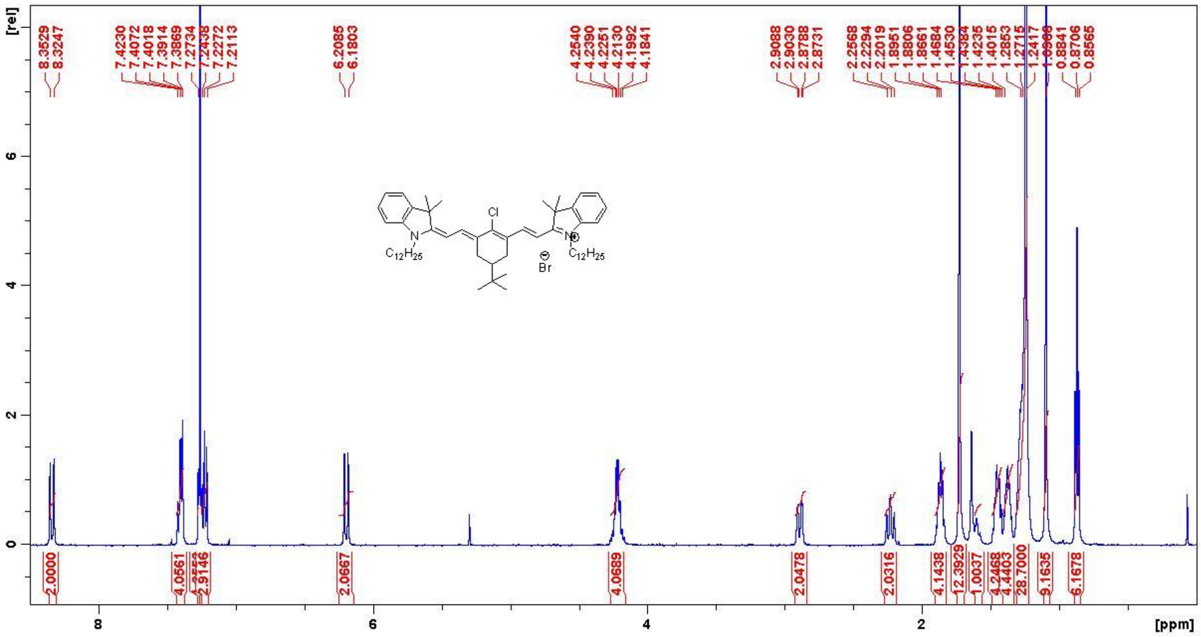
[rel]
[ppm]
Cl
N
C12H25
Br

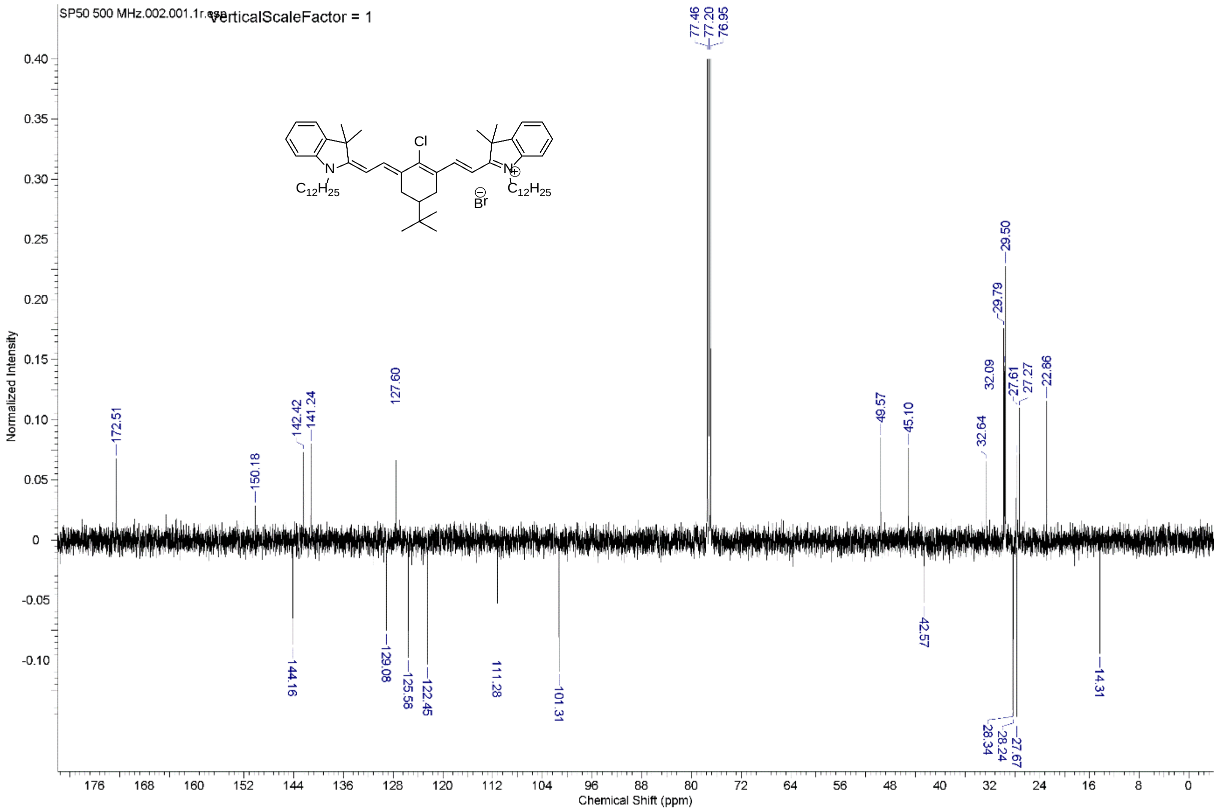
SP50 500 MHz.002.001.1r.esp
VerticalScaleFactor = 1
Normalized Intensity
Chemical Shift (ppm)
Cl
N
C12H25
Br

Compound D^2D^2=O

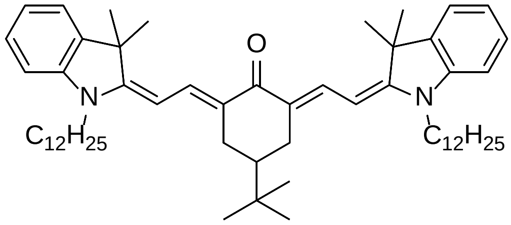

To a solution of 90 mg of **D^2D^2** (0.10 mmol, 1 equiv.) and 17 mg of *N*-hydroxysuccinimide (0.15 mmol, 1.5 equiv.) in 5 mL of anhydrous DMF were added 30 µL of distilled DIEA (0.19 mmol, 2 equiv.) and the mixture was stirred for 5 h at RT. The solution was evaporated and the residue was dissolved in 10 mL of DCM and washed with an aqueous saturated solution of NH_4Cl (10 mL). The organic layer was dried over $MgSO_4$ and concentrated. The crude solid was purified by flash chromatography on silica, using EtOAc/PE as eluent (1:9, R_f = 0.47) to afford the product as reddish solid in 79% yield (63 mg).

^{1}H NMR ($CDCl_3$, 500.10 MHz): δ 8.14 (d, ^3J = 13 Hz, 2H, =C**H**), 7.19-7.17 (m, 4H, C**H**$_{Ar}$), 6.90 (dd, ^3J = 7 Hz, 2H, C**H**$_{Ar}$), 6.67 (d, ^3J = 8 Hz, 2H, C**H**$_{Ar}$), 5.47 (d, ^3J = 13 Hz, 2H, =C**H**), 3.66 (t, ^3J = 7 Hz, 4H, N-C**H**$_2$), 2.85 (d, ^2J = 13 Hz, 2H, **H**$_{eq}$), 2.12 (dd, ^2J = 14 Hz, ^3J = 14 Hz, 2H, **H**$_{ax}$), 1.72 (t, ^3J = 7 Hz, 4H, C**H**$_2$), 1.67 (s, 6H, C(**CH$_3$**)$_2$), 1.66 (s, 6H, C(**CH$_3$**)$_2$), 1.55 (m, 1H, C**H**), 1.44-1.33 (m, 8H, C**H**$_2$), 1.26 (m, 28H, C**H**$_2$), 1.05 (s, 9H, C(C**H**$_3$)$_3$), 0.88 (t, ^3J = 7 Hz, 6H, C**H**$_3$-CH_2).

^{13}C NMR ($CDCl_3$, 125.75 MHz): δ 186.6 (C_{quat}), 162.4 (C_{quat}), 144.4 (C_{quat}), 139.9 (C_{quat}), 132.9 (CH), 127.8 (CH), 126.6 (C_{quat}), 121.9 (CH), 120.5 (CH), 106.8 (CH), 92.5 (CH), 46.7 (C_{quat}), 43.7 (CH), 42.8 (N-CH_2), 32.8 (C_{quat}), 32.1 (CH_2), 29.8 (CH_2), 29.8 (CH_2), 29.8 (CH_2), 29.7 (CH_2), 29.6 (CH_2), 29.5 (CH_2), 28.9 (CH_2), 28.9 (CH_3), 27.7 (CH_3), 27.4 (CH_2), 26.9 (CH_2), 26.3 (CH_2), 22.8 (CH_2), 14.2 (CH_3).

UV-Vis (CH_3OH): λ_{max} = 531 nm (ε_{max} = 54000 $L.mol^{-1}.cm^{-1}$).

UV-Vis (CH_3OH+CH_3CO_2H): λ_{max} = 712 nm (ε_{max} = 116000 $L.mol^{-1}.cm^{-1}$).

HRMS (ESI+): $[M+H]^+$ = 829.6936 (calcd for $C_{58}H_{89}N_2O^+$: 829.6969).

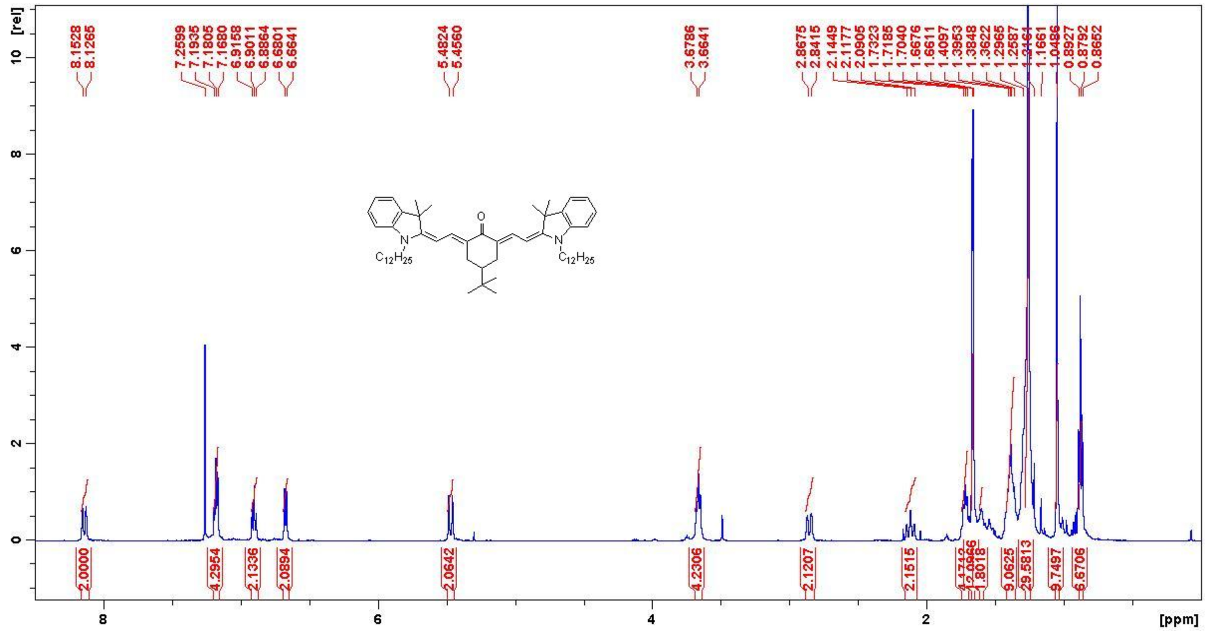

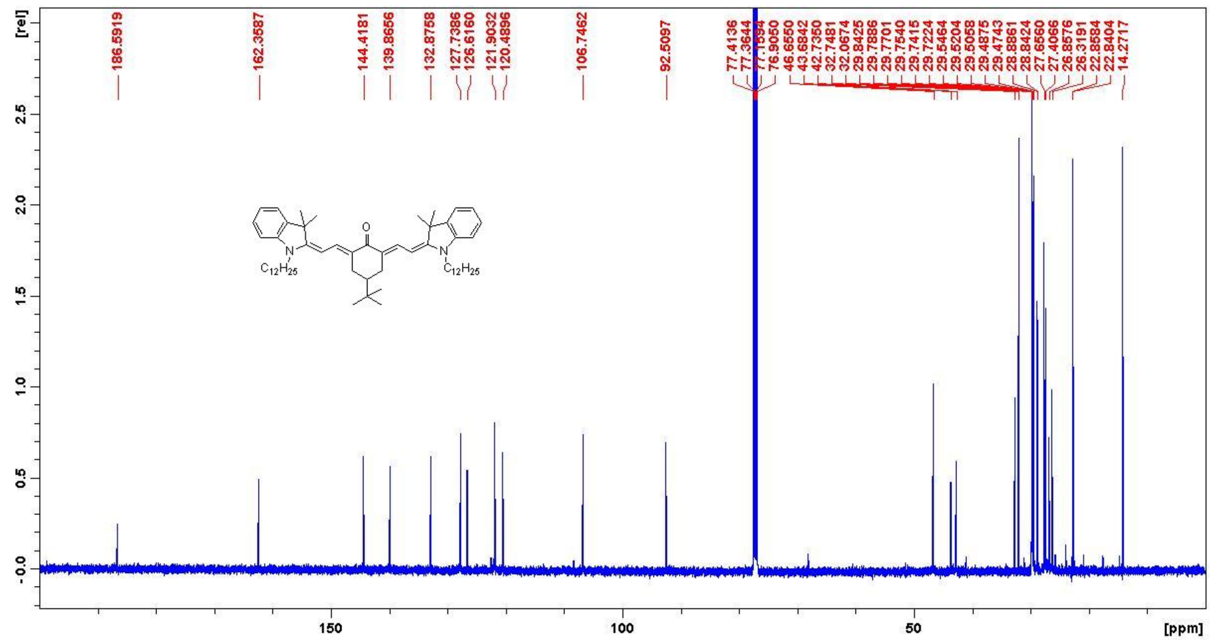

Compound D^5D^5

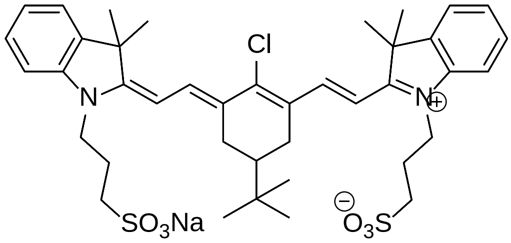

To a solution of 74 mg of **1** (0.32 mmol, 1 equiv.) and 200 mg of $\mathbf{D^5}$ (0.71 mmol, 2.2 equiv.) in 10 mL of anhydrous ethanol were added 70 µL of distilled pyridine (0.81 mmol, 2.5 equiv.). The solution was stirred for 15 h at 80 °C. The resulting solution was concentrated under reduced pressure, and the crude residue was submitted to flash chromatography on silica gel using $CH_3CN/MeOH/H_2O$ (85:10:5, R_f = 0.36) as eluent to afford the product as a dark greenish solid in 90% yield (220 mg).

^{1}H NMR (CD_3OD, 500.10 MHz): δ 8.46 (d, ^3J = 14 Hz, 2H, =C**H**), 7.51 (d, ^3J = 7 Hz, 2H, C$\mathbf{H}_{Ar}$), 7.43-7.41 (m, 4H, C$\mathbf{H}_{Ar}$), 7.29-7.26 (m, 2H, C$\mathbf{H}_{Ar}$), 7.50 (d, ^3J = 14 Hz, 2H, =C**H**), 4.42 (m, 4H, C$\mathbf{H}_2$), 3.07 (d, ^2J = 15 Hz, 2H, $\mathbf{H}_{eq}$), 2.95 (t, ^3J = 6 Hz, 4H, C$\mathbf{H}_2$), 2.30-2.24 (m, 6H, C$\mathbf{H}_2$ and $\mathbf{H}_{ax}$), 1.75 (s, 12H, C(C$\mathbf{H}_3$)$_2$), 1.54 (m, 1H, C**H**-C(CH_3)$_3$), 1.14 (s, 9H, C(C$\mathbf{H}_3$)$_3$).

^{13}C NMR (CD_3OD, 125.75 MHz): δ 174.3 (C_{quat}), 151.3 (C_{quat}), 145.9 (CH), 143.6 (C_{quat}), 142.7 (C_{quat}), 129.9 (CH), 129.3 (C_{quat}), 126.4 (CH), 123.4 (CH), 112.3 (CH), 102.6 (CH), 49.7 (C_{quat}), 49.0 (CH_2), 44.4 (CH), 46.0 (CH_2), 33.5 (C_{quat}), 29.0 (CH_2), 28.4 (CH_3), 28.1 (CH_3), 24.2 (CH_2).

UV-Vis (H_2O): λ_{max} = 774 nm (ε_{max} = 130000 L.mol^{-1}.cm^{-1}).

UV-Vis (CH_3OH): λ_{max} = 782 nm (ε_{max} = 223000 L.mol^{-1}.cm^{-1}).

HRMS (ESI-): [M−Na]$^-$ = 753.2788 (calcd for $C_{40}H_{50}ClN_2O_6S_2^-$: 753.2804).

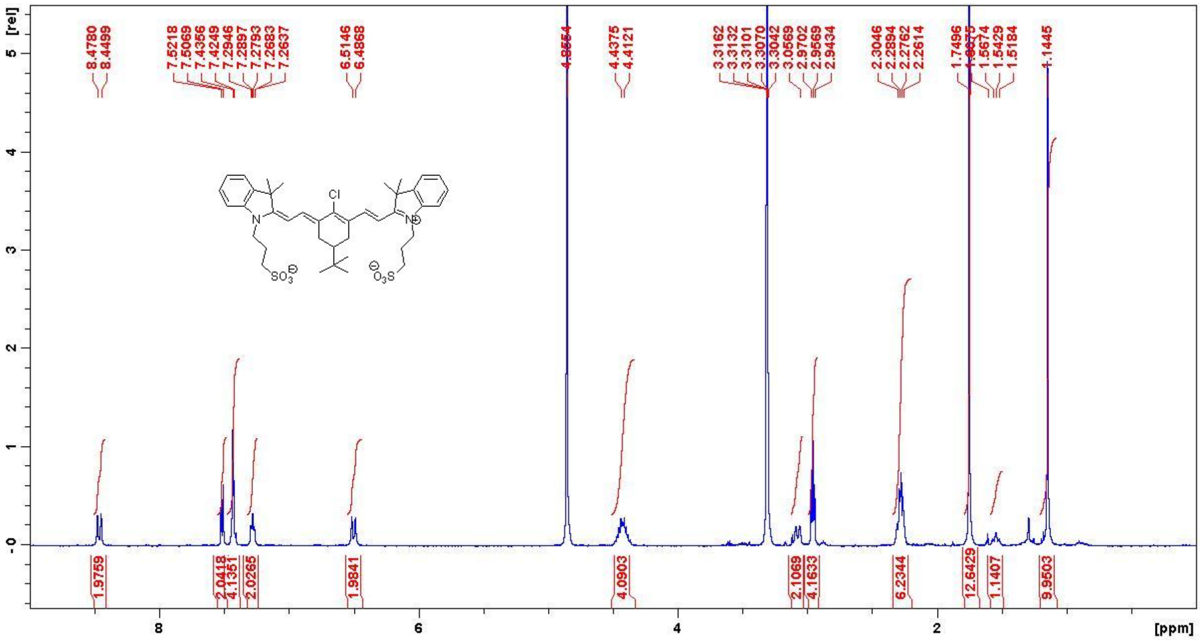

8.4780
8.4499
7.5218
7.5069
7.4356
7.4249
7.2946
7.2897
7.2793
7.2683
7.2637
6.5146
6.4868
4.4375
4.4121
3.3162
3.3132
3.3101
3.3070
3.3042
3.0569
2.9702
2.9569
2.9434
2.3046
2.2894
2.2762
2.2614
1.7496
1.5674
1.5429
1.5184
1.1445
1.9759
2.0418
4.1361
2.0265
1.9841
4.0903
2.1069
4.1633
6.2344
12.6429
1.1407
9.9503
[rel]
[ppm]

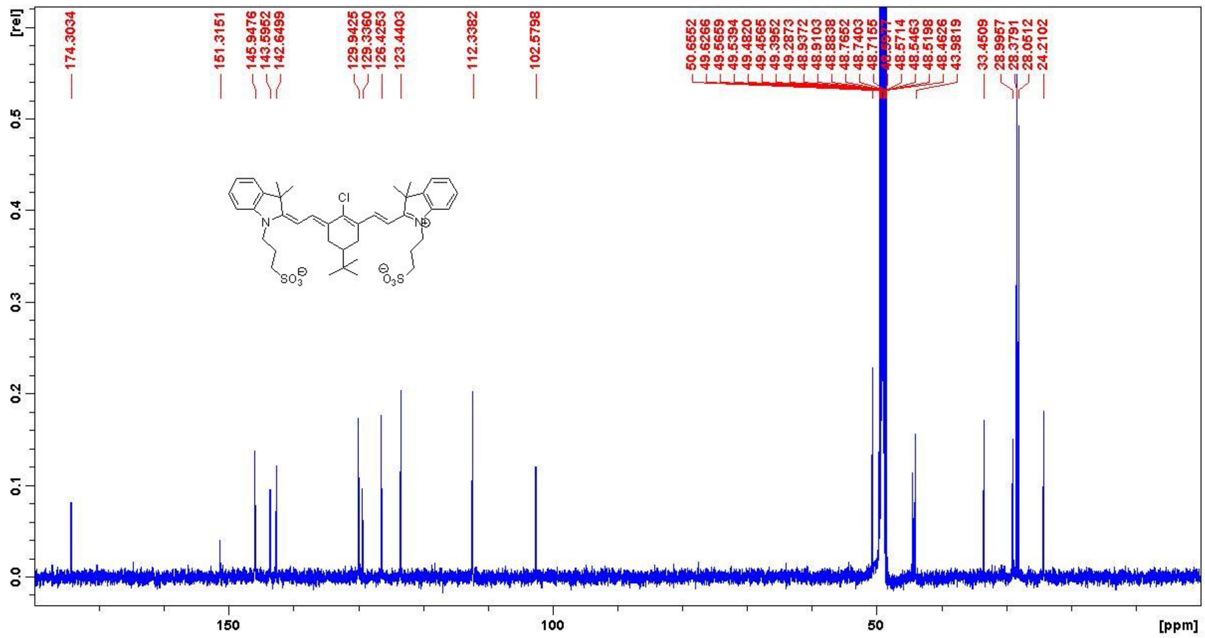

174.3034
151.3151
145.9476
143.5952
142.6499
129.9425
129.3360
126.4253
123.4403
112.3382
102.5798
50.6552
49.6266
49.5559
49.5394
49.4820
49.4565
49.3952
49.2873
48.9372
48.9103
48.8838
48.7652
48.7403
48.7155
48.5714
48.5463
48.5198
48.4626
43.9819
33.4509
28.9957
28.3791
28.0512
24.2102
[rel]
[ppm]

Compound D^5D^5=O

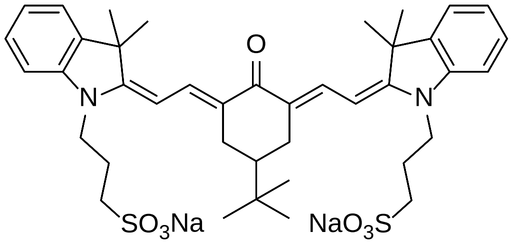

To a solution of 100 mg of **D^5D^5** (0.13 mmol, 1 equiv.) and 30 mg of *N*-hydroxysuccinimide (0.17 mmol, 2 equiv.) in 5 mL of anhydrous DMF were added 50 µL of distilled DIEA (0.29 mmol, 2.2 equiv.) and the mixture was stirred for 3 h at RT. The solvent was removed under reduced pressure and the crude residue was submitted to flash chromatography on silica gel using acetone/MeOH/H_2O (90:5:5, R_f = 0.52) as eluent. The product was isolated as deep purple solid in 85% yield (83 mg).

^1H NMR (DMSO-d6, 500.10 MHz): δ 7.90 (d, ^3J = 13 Hz, 2H, =C**H**), 7.31 (d, ^3J = 7 Hz, 2H, C$\mathbf{H}_{Ar}$), 7.17 (t, ^3J = 7 Hz, 2H, C$\mathbf{H}_{Ar}$), 6.95 (d, ^3J = 7 Hz, 2H, C$\mathbf{H}_{Ar}$), 6.88 (t, ^3J = 7 Hz, 2H, C$\mathbf{H}_{Ar}$), 5.59 (d, ^3J = 13 Hz, 2H, =C**H**), 3.90 (m, 4H, N-C$\mathbf{H}_2$), 2.86 (m, 2H, $\mathbf{H}_{eq}$), 2.51 (m, 4H, C$\mathbf{H}_2$-SO_3^-), 2.00 (dd, ^2J = 14 Hz, ^3J = 14 Hz, 2H, $\mathbf{H}_{ax}$), 1.92 (m, 4H, C$\mathbf{H}_2$), 1.55 (s, 12H, C(C$\mathbf{H}_3$)$_2$), 1.30 (m, 1H, C**H**-C(CH_3)$_3$), 1.02 (s, 9H, C(C$\mathbf{H}_3$)$_3$).

^{13}C NMR (DMSO-d6, 125.75 MHz): δ 184.9 (C=O), 161.6 (C_{quat}), 144.0 (C_{quat}), 138.9 (C_{quat}), 132.0 (=CH), 127.9 (CH), 126.1 (C_{quat}), 121.7 (CH), 120.4 (CH), 107.4 (CH), 92.3 (=CH), 46.2 (CH_2), 45.9 (C_{quat}), 43.6 (CH), 40.9 (CH_2), 32.5 (C_{quat}), 28.3 (CH_3), 27.5 (CH_3), 26.5 (CH_2), 22.4 (CH_2).

UV-Vis (H_2O): λ_{max} = 567 nm (ε_{max} = 38000 $L.mol^{-1}.cm^{-1}$).

UV-Vis (CH_3OH): λ_{max} = 527 nm (ε_{max} = 35000 $L.mol^{-1}.cm^{-1}$).

HRMS (ESI-): $[M-2Na]^{2-}/2$ = 367.1518 (calcd for $C_{40}H_{50}N_2O_7S_2^{2-}$: 367.1535).

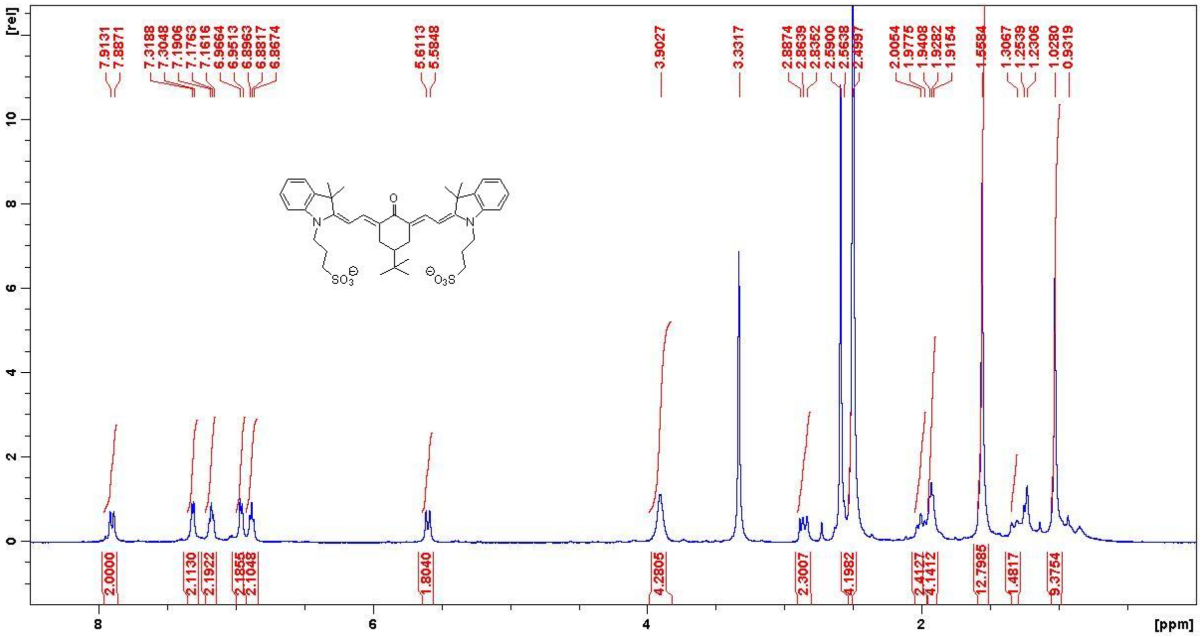

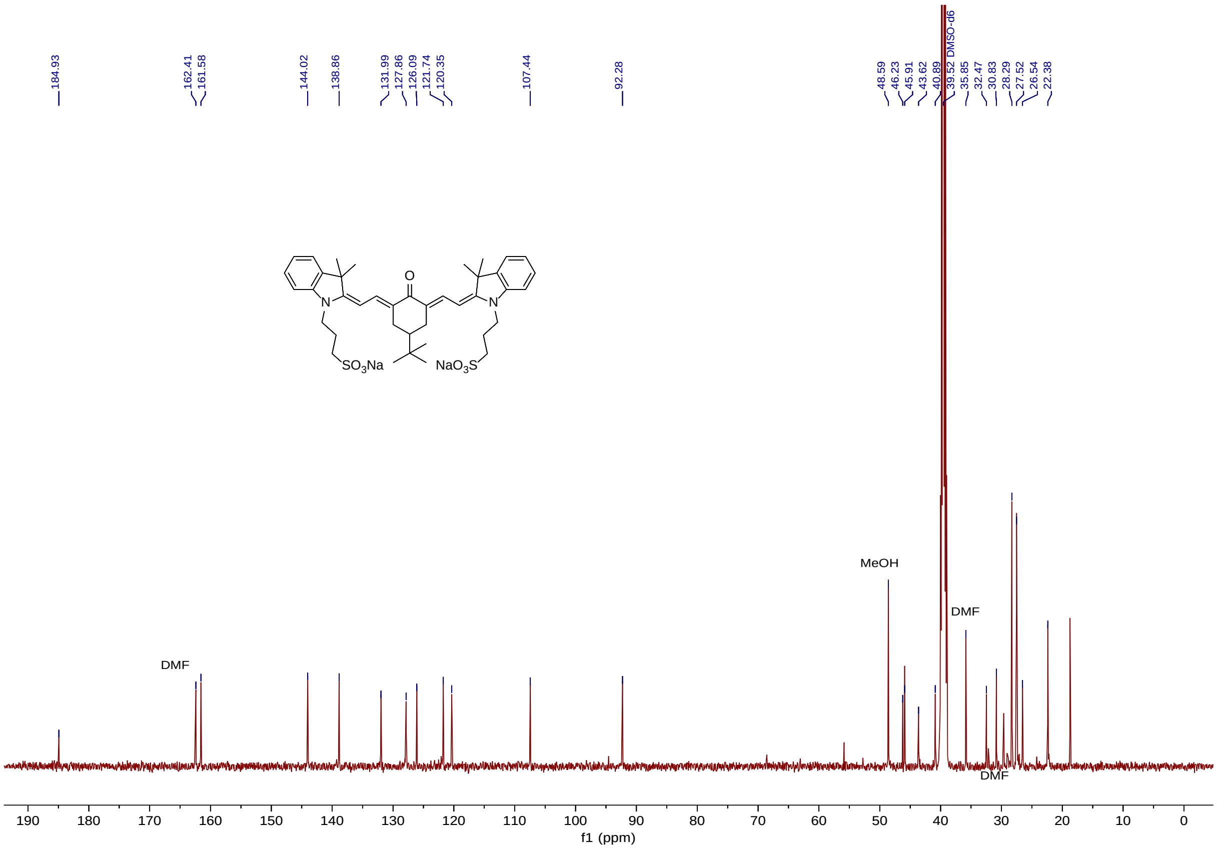

Compound 3

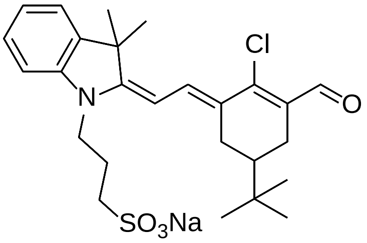

To a solution of 244 mg of **1** (1.07 mmol, 1 equiv.) in 10 mL of absolute ethanol were added dropwise 300 mg of **D**[5] (1.07 mmol, 1 equiv.) in 10 mL of anhydrous ethanol over 1 h. The mixture was stirred for 18 h at 80 °C. The resulting solution was concentrated under reduced pressure, and the crude residue was submitted to flash chromatography on silica gel using $CH_3CN/MeOH/H_2O$ (85:5:5) as eluent to afford the product as a dark orange solid in a 74% yield (386 mg).

^{1}H NMR (DMSO-d6, 500.10 MHz): δ 10.10 (s, 1H, C**H**O), 7.78 (d, ^3J = 14 Hz, 1H, =C**H**), 7.33 (d, ^3J = 7 Hz, 1H, C**H**$_{Ar}$), 7.20 (t, ^3J = 7 Hz, 1H, C**H**$_{Ar}$), 7.00 (d, ^3J = 7 Hz, 1H, C**H**$_{Ar}$), 6.92 (t, ^3J = 7 Hz, 1H, C**H**$_{Ar}$), 5.83 (d, ^3J = 14 Hz, 1H, =C**H**), 3.97 (m, 2H, N-C**H**$_2$), 2.98 (d, ^3J = 15 Hz, 1H, **H$_{eq}$**), 2.69 (d, ^3J = 15 Hz, 1H, **H$_{eq}$**), 2.50 (revealed by HSQC, 2H, C**H**$_2$-SO_3^-), 1.96 (dd, ^2J = 13 Hz, ^3J = 13 Hz, 1H, **H**$_{ax}$), 1.92 (m, 2H, C**H**$_2$), 1.83 (dd, ^2J = 13 Hz, ^3J = 13 Hz, 1H, **H**$_{ax}$), 1.58 (s, 6H, C(C**H**$_3$)$_2$), 1.28 (m, 1H, C**H**), 0.96 (s, 9H, C(C**H**$_3$)$_3$).

^{13}C NMR (DMSO-d6, 125.75 MHz): δ 189.1 (CHO), 162.4 (C_{quat}), 147.5 (C_{quat}), 143.7 (C_{quat}), 138.8 (C_{quat}), 131.7 (=CH), 127.9 (CH), 127.4 (C_{quat}), 122.8 (C_{quat}),121.8 (CH), 120.8 (CH), 107.9 (CH), 93.2 (=CH), 48.3 (CH_2), 46.1 (C_{quat}), 42.1 (CH), 40.9 (CH_2), 32.1 (C_{quat}), 28.0 (CH_3), 27.9 (CH_3), 27.4 (CH_2), 27.2 (CH_3), 25.7 (CH_2), 22.5(CH_2).

UV-Vis (CH_3OH): λ_{max} = 488 nm (ε_{max} = 46000 L.mol^{-1}.cm^{-1}).

HRMS (ESI-): [M−Na]$^-$ = 490.1839 (calcd for $C_{26}H_{33}ClNO_4S^-$: 490.1824).

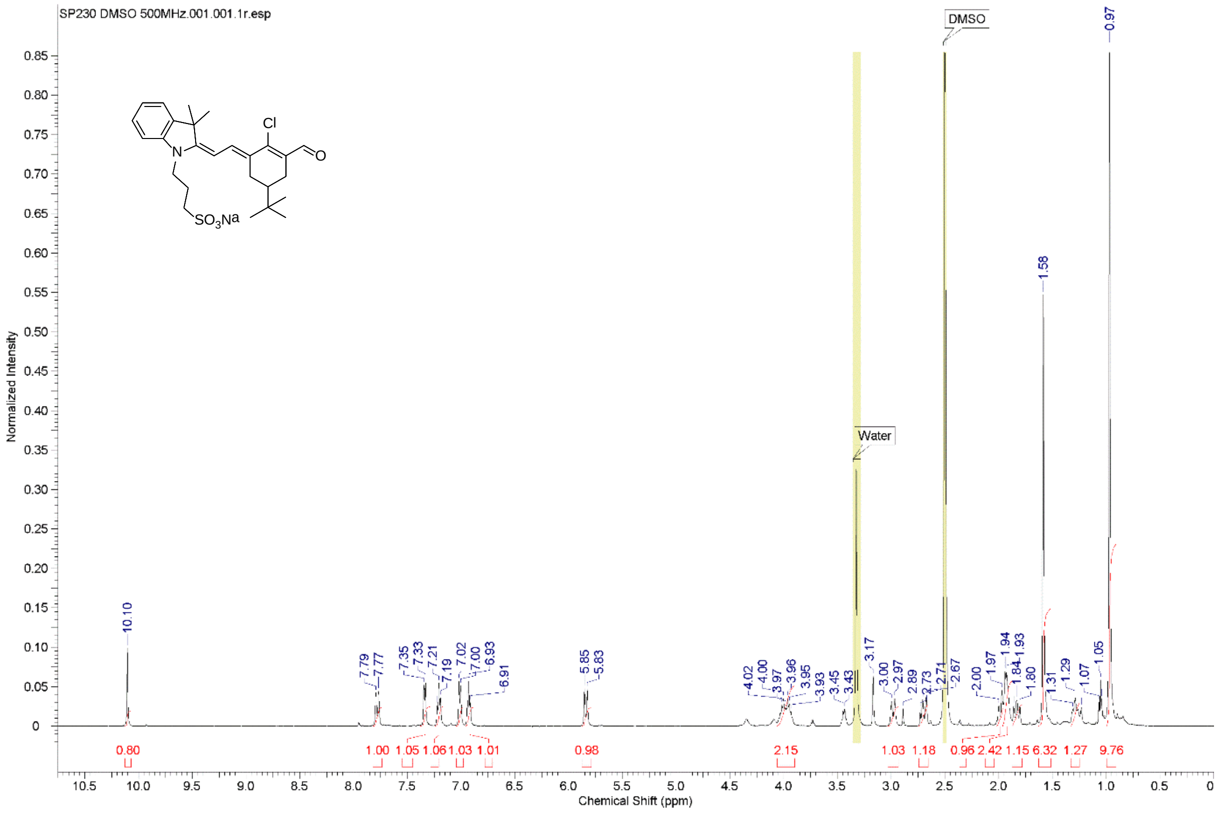
SP230 DMSO 500MHz.001.001.1r.esp
Normalized Intensity
Chemical Shift (ppm)
Water
DMSO
SO3Na
Cl
N
O

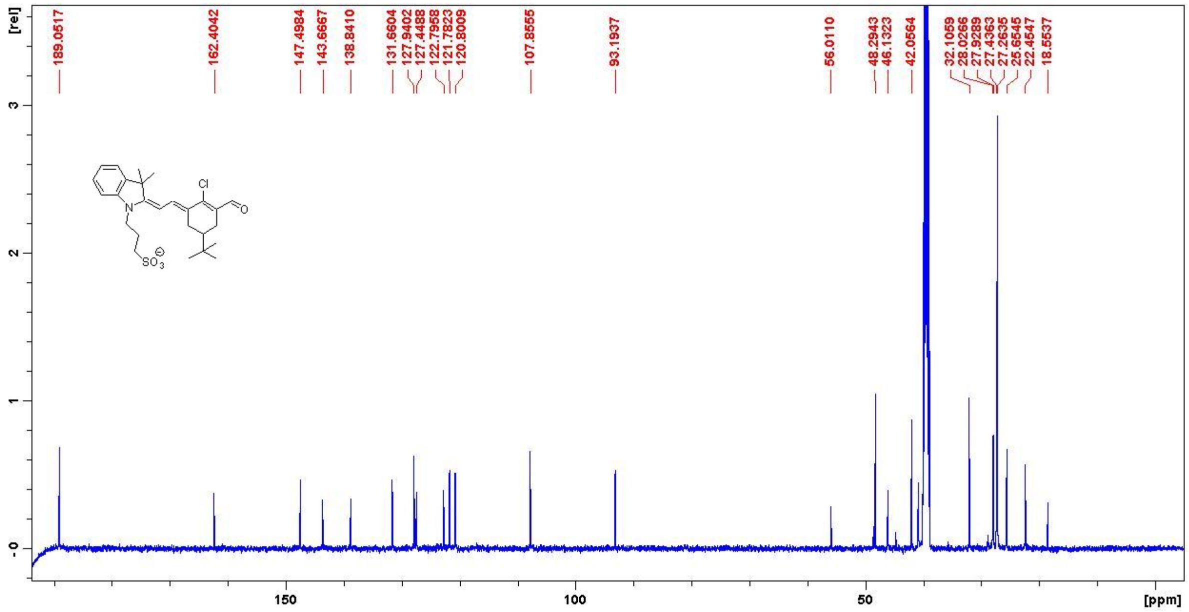
[rel]
[ppm]
189.0517
162.4042
147.4984
143.6667
138.8410
131.6604
127.9402
127.4488
122.7958
121.7823
120.8009
107.8555
93.1937
56.0110
48.2943
46.1323
42.0564
32.1059
28.0266
27.9289
27.4363
27.2635
25.6545
22.4547
18.5537

Compound D^2D^5

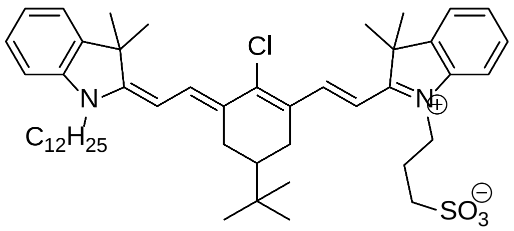

To a solution of 200 mg of compound **3** (0.41 mmol, 1 equiv.) and 200 mg of $\mathbf{D^2}$ (0.49 mmol, 1.2 equiv.) in 5 mL of anhydrous ethanol were added 50 µL of pyridine (0.61 mmol, 1.5 equiv.). The mixture was stirred for 1.5h at 80 °C. The resulting solution was concentrated under reduced pressure, and the crude residue was submitted to flash chromatography on silica gel using DCM/MeOH (9:1, R_f = 0.47) as eluent to afford the product as a dark greenish solid in a 88% yield (288 mg).

^{1}H NMR ($CDCl_3$, 500.10 MHz): δ 8.51 (d, ^3J = 14 Hz, 1H, =C**H**), 8.17 (d, ^3J = 13 Hz, 1H, =C**H**), 7.46-7.40 (m, 2H, C$\mathbf{H}_{Ar}$), 7.37-7.31 (m, 4H, C$\mathbf{H}_{Ar}$), 7.16-7.12 (m, 2H, C$\mathbf{H}_{Ar}$ and =C**H**), 6.95 (d, ^3J = 8 Hz, 1H, C$\mathbf{H}_{Ar}$), 5.87 (d, ^3J = 13 Hz, 1H, =C**H**), 4.84 (dt, ^2J = 13 Hz, ^3J = 7Hz, 1H, N-C$\mathbf{H}_2$), 4.51 (dt, ^2J = 13 Hz, ^3J = 7Hz, 1H, N-C$\mathbf{H}_2$), 3.88 (t, ^3J = 7 Hz, 2H, N-C$\mathbf{H}_2$), 3.22 (d, ^2J = 16 Hz, 1H, $\mathbf{H}_{eq}$), 2.99 (m, 2H, C$\mathbf{H}_2$), 2.84 (d, ^2J = 16 Hz, 1H, $\mathbf{H}_{eq}$), 2.44 (dd, ^2J = 15 Hz, ^3J = 15 Hz, 1H, $\mathbf{H}_{ax}$), 2.34 (m, 2H, C$\mathbf{H}_2$), 2.12 (dd, ^2J = 13 Hz, ^3J = 13 Hz, 1H, $\mathbf{H}_{ax}$), 1.80 (t, ^3J = 7 Hz, 2H, C$\mathbf{H}_2$), 1.73 (s, 3H, C(C$\mathbf{H}_3$)$_2$), 1.71 (s, 3H, C(C$\mathbf{H}_3$)$_2$), 1.62 (s, 6H, C(C$\mathbf{H}_3$)$_2$), 1.56 (m, 1H, C**H**), 1.46-1.34 (m, 4H, C$\mathbf{H}_2$), 1.32-1.22 (m, 14H, C$\mathbf{H}_2$), 1.14 (s, 9H, C(C$\mathbf{H}_3$)$_3$), 0.87 (t, ^3J = 7 Hz, 3H, C$\mathbf{H}_3$).

^{13}C NMR ($CDCl_3$, 125.75 MHz): δ 176.0 (C_{quat}), 168.6 (C_{quat}), 150.5 (C_{quat}), 148.7 (CH), 142.9 (C_{quat}), 142.0 (C_{quat}), 141.8 (C_{quat}), 140.7 (CH), 140.5 (C_{quat}), 130.7 (C_{quat}), 129.3 (CH), 128.6 (CH), 127.8 (C_{quat}), 126.6 (CH), 123.8 (CH), 122.4 (CH), 122.3 (CH), 112.2 (CH), 109.4 (CH), 106.2 (CH), 98.0 (CH), 50.2 (C_{quat}), 48.4 (C_{quat}), 47.0 (CH_2), 44.3 (CH_2), 44.0 (CH_2), 42.8 (CH), 32.7 (C_{quat}), 32.0 (CH_2), 29.7 (CH_2), 29.6 (CH_2), 29.4 (CH_2), 29.4 (CH_2), 28.5 (CH_2), 28.4 (CH_2), 28.3 (CH_2), 28.3 (CH_3), 28.1 (CH_3), 27.7 (CH_3), 27.3 (CH_2), 27.0 (CH_2), 24.4 (CH_2), 22.8 (CH_2), 14.3 (CH_3).

UV-Vis (CH_3OH): λ_{max} = 782 nm (ε_{max} = 270000 L.mol^{-1}.cm^{-1}).

HRMS (ESI+): $[M+H]^+$ = 801.4771 (calcd for $C_{49}H_{70}ClN_2O_3S^+$: 801.4790).

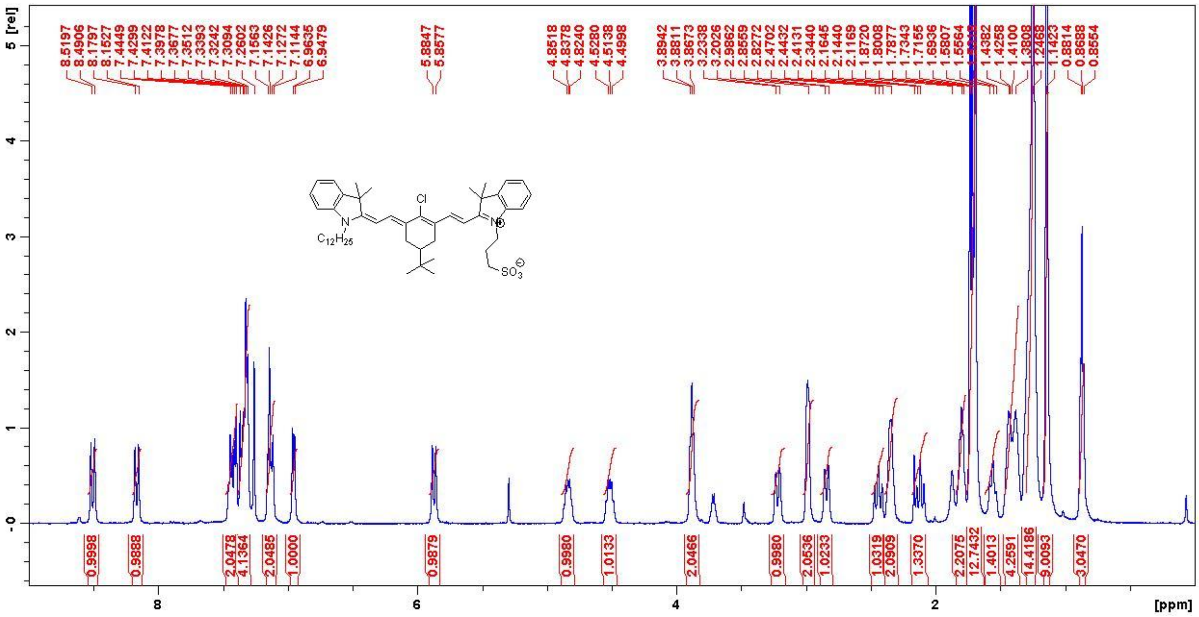
8.5197
8.4906
8.1797
8.1527
7.4449
7.4299
7.4122
7.3978
7.3677
7.3512
7.3393
7.3242
7.3094
7.2602
7.1563
7.1426
7.1272
7.1144
6.9635
6.9479
5.8847
5.8577
4.8518
4.8378
4.8240
4.5280
4.5138
4.4998
3.8942
3.8811
3.8673
3.2338
3.2026
2.9862
2.8559
2.8272
2.4702
2.4432
2.4131
2.3440
2.1645
2.1440
2.1169
1.8720
1.8008
1.7877
1.7343
1.7155
1.6936
1.5807
1.5564
1.4382
1.4258
1.4100
1.3808
1.2468
1.1423
0.8814
0.8688
0.8554
0.9998
0.9888
2.0478
4.1364
2.0485
1.0000
0.9879
0.9980
1.0133
2.0466
0.9980
2.0536
1.0233
1.0319
2.0909
1.3370
2.2075
12.7432
1.4013
4.2591
14.4186
9.0093
3.0470
Cl
N
C12H25
N⊕
SO3⊖
[rel]
[ppm]

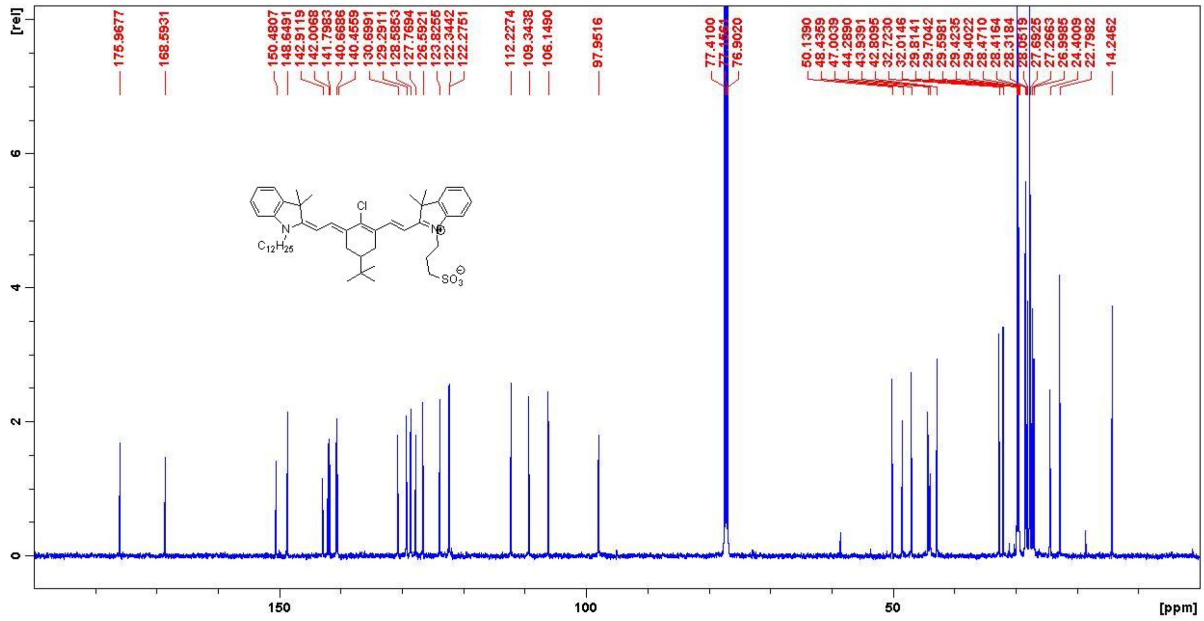
175.9677
168.5931
150.4807
148.6491
142.9119
142.0068
141.7983
140.6686
140.4559
130.6991
129.2911
128.5853
127.7694
126.5921
123.8255
122.3442
122.2751
112.2274
109.3438
106.1490
97.9516
77.4100
76.9020
50.1390
48.4359
47.0039
44.2890
43.9391
42.8095
32.7230
32.0146
29.8141
29.7042
29.5981
29.4235
29.4022
28.4710
28.4164
28.3184
28.0519
27.6925
27.2663
26.9985
24.4009
22.7982
14.2462
Cl
N
C12H25
N⊕
SO3⊖
[rel]
[ppm]

Compound D^2D^5=O

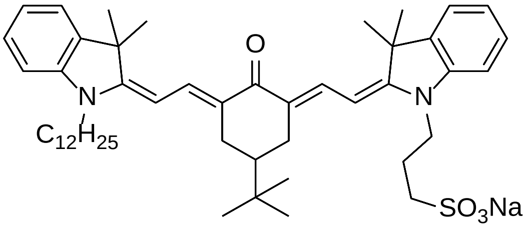

To solution of 130 mg of $\mathbf{D^2D^5}$ (0.16 mmol, 1 equiv.) and 37 mg of *N*-hydroxysuccinimide (0.32 mmol, 2 equiv.) in 3 mL of anhydrous DMF were added 70 µL of distilled DIEA (0.41 mmol, 2.5 equiv.) and the mixture was stirred for 3 h at RT. The solvent was removed under reduced pressure, the residue was dissolved in DCM and washed with a saturated aqueous ammonium chloride solution, dried over Na_2SO_4 and concentrated. The crude residue was submitted to flash chromatography on silica gel using $CHCl_3$/MeOH (85:15, R_f = 0.29) as eluent. The product was isolated as deep purple solid in a 69% yield (88 mg).

^{1}H NMR (DMSO-d6, 500.10 MHz): δ 7.92 (d, ^3J = 14 Hz, 1H, =C**H**), 7.89 (d, ^3J = 14 Hz, 1H, =C**H**), 7.32 (d, ^3J = 7 Hz, 2H, C$\mathbf{H}_{Ar}$), 7.18 (m, 2H, C$\mathbf{H}_{Ar}$), 6.98 (d, ^3J = 7 Hz, 2H, C$\mathbf{H}_{Ar}$), 6.89 (m, 2H, C$\mathbf{H}_{Ar}$), 5.64 (d, ^3J = 13 Hz, 1H, =C**H**), 5.49 (d, ^3J = 13 Hz, 1H, =C**H**), 3.92 (m, 2H, C$\mathbf{H}_2$), 3.75 (m, 2H, C$\mathbf{H}_2$), 2.86 (d, ^2J = 15 Hz, 1H, $\mathbf{H}_{eq}$), 2.80 (d, ^2J = 14 Hz, 1H, $\mathbf{H}_{eq}$), 2.01 (m, 2H, C$\mathbf{H}_2$), 1.94 (m, 2H, C$\mathbf{H}_2$), 1.62 (m, 2H, C$\mathbf{H}_2$), 1.55 (s, 12H, C(C$\mathbf{H}_3$)$_2$), 1.34 (m, 5H, C$\mathbf{H}_2$ and C**H**), 1.22 (m, 16H, C$\mathbf{H}_2$), 1.02 (s, 9H, C(C$\mathbf{H}_3$)$_3$), 0.84 (t, ^3J = 7 Hz, 3H, C$\mathbf{H}_3$).

^{13}C NMR (DMSO-d6, 125.75 MHz): Compound was too insoluble to record a ^{13}C spectrum.

UV-Vis (CH_3OH): λ_{max} = 530 nm (ε_{max} = 67000 L.mol^{-1}.cm^{-1})

UV-Vis (CH_3OH + CH_3CO_2H): λ_{max} = 711 nm (ε_{max} = 170000 L.mol^{-1}.cm^{-1}).

HRMS (ESI-): [M−Na]$^-$ = 781.4998 (calcd for $C_{49}H_{69}N_2O_4S^-$: 781.4984).

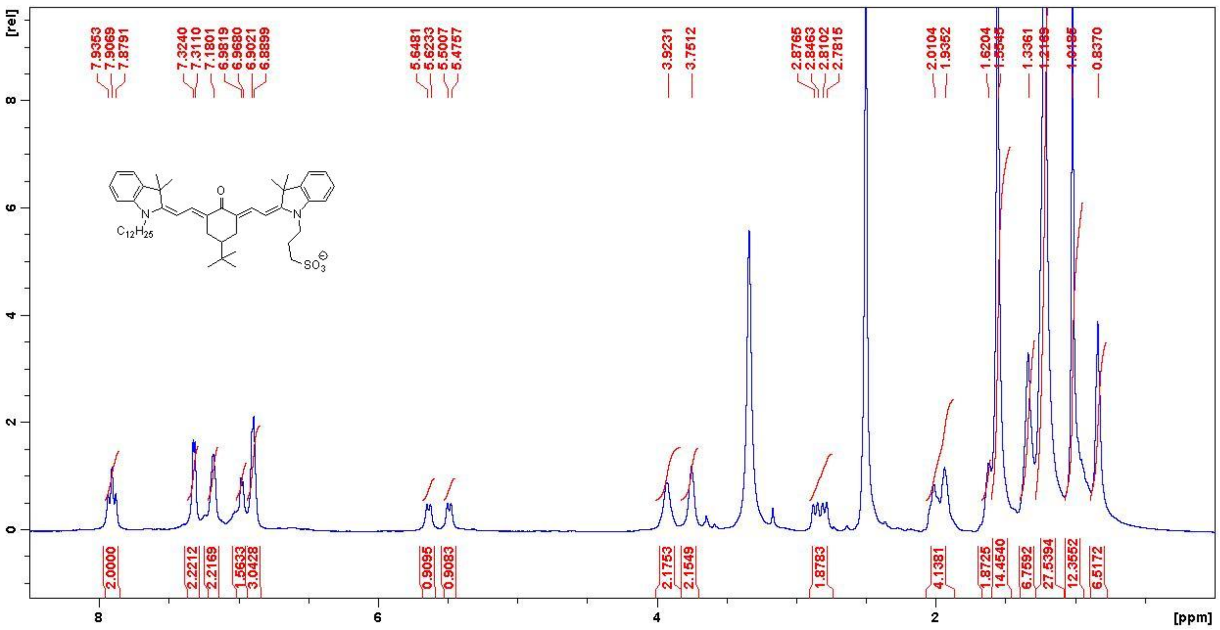
[rel]
7.9353
7.9069
7.8791
7.3240
7.3110
7.1801
6.9819
6.9680
6.9021
6.8899
5.6481
5.6233
5.5007
5.4757
3.9231
3.7512
2.8765
2.8463
2.8102
2.7815
2.0104
1.9352
1.6204
1.3361
0.8370
2.0000
2.2212
2.2169
1.5633
3.0428
0.9095
0.9083
2.1753
2.1549
1.8783
4.1381
1.8725
14.4540
6.7592
27.5394
12.3552
6.5172
[ppm]

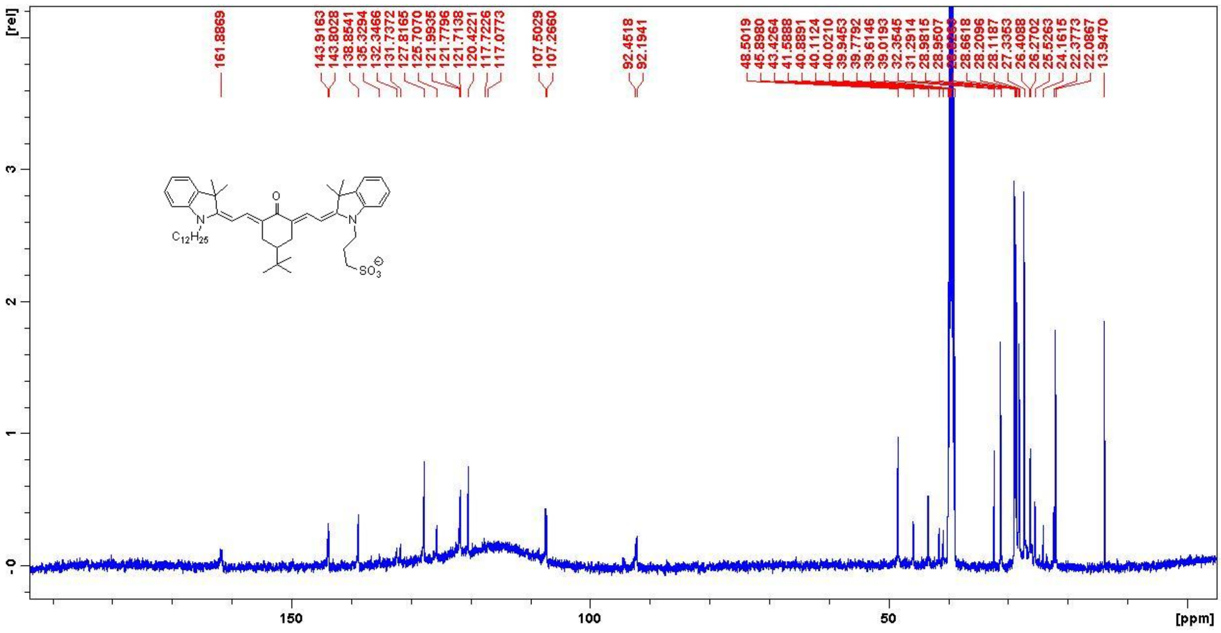
[rel]
161.8869
143.9163
143.8028
138.8541
135.3294
132.3466
131.7372
127.8165
125.7070
121.9935
121.7796
121.7138
120.4221
117.7226
117.0773
107.5029
107.2660
92.4518
92.1941
48.5019
45.8980
43.4264
41.5888
40.8891
40.1124
40.0210
39.9453
39.7792
39.6146
39.0193
32.3545
31.2914
28.9815
28.9507
28.6618
28.2096
28.1187
27.3353
26.4088
26.2702
25.5263
24.1615
22.3773
22.0867
13.9470
[ppm]

Compound D^4D^4

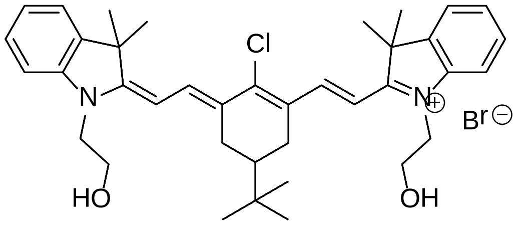

To solution of 628 mg of **1** (2.75 mmol, 1 equiv.) and 2 g of $\mathbf{D^4}$ (6.04 mmol, 2.2 equiv.) in 20 mL of absolute ethanol was added 0.55 mL of pyridine (6.86 mmol, 2.5 equiv.). The solution was stirred for 17 h at 80 °C. The resulting green solution was then concentrated, added by 20 mL DCM and washed with an aqueous solution of HBr 1M and water. The organic layer was dried over Na_2SO_4, filtered and concentrated. The crude residue was purified by flash chromatography on silica gel using DCM/MeOH as eluent (from 95:5 to 90:10, R_f = 0.42) to afford a greenish solid in a 71% yield (1.335 g).

^{1}H NMR ($CDCl_3$, 500.10 MHz): δ 8.35 (d, ^3J = 14 Hz, 2H, =C**H**), 7.39-7.33 (m, 6H, C$\mathbf{H}_{Ar}$), 7.23-7.19 (m, 2H, C$\mathbf{H}_{Ar}$), 6.38 (d, ^3J = 14 Hz, 2H, =C**H**), 4.35 (m, 4H, C$\mathbf{H}_2$), 4.05 (m, 4H, C$\mathbf{H}_2$), 2.91 (dd, ^2J = 15 Hz, ^3J = 2 Hz, 2H, $\mathbf{H}_{eq}$), 2.26 (dd, ^2J = 14 Hz, ^3J = 14 Hz, 2H, $\mathbf{H}_{ax}$), 1.73 (s, 6H, C(C$\mathbf{H}_3$)$_2$), 1.72 (s, 6H, C(C$\mathbf{H}_3$)$_2$), 1.53 (m, 1H, C**H**-C(CH_3)$_3$), 1.10 (s, 9H, C(C$\mathbf{H}_3$)$_3$).

^{13}C NMR ($CDCl_3$, 125.75 MHz): δ 173.3 (C_{quat}), 150.5 (C_{quat}), 144.7 (CH), 142.8 (C_{quat}), 141.0 (C_{quat}), 129.0 (CH), 128.2 (C_{quat}), 125.3 (CH), 122.1 (CH), 111.8 (CH), 101.9 (CH), 58.9 (CH_2), 49.5 (C_{quat}), 47.5 (CH_2), 43.0 (CH), 32.7 (C_{quat}), 28.4 (CH_3), 28.4 (CH_3), 28.1 (CH_2), 27.9 (CH_3).

UV-Vis (CH_3OH): λ_{max} = 780 nm (ε_{max} = 161000 L.mol^{-1}.cm^{-1}).

HRMS (ESI+): $[M-Br]^+$ = 599.3392 (calcd for $C_{38}H_{48}ClN_2O_2^+$: 599.3399).

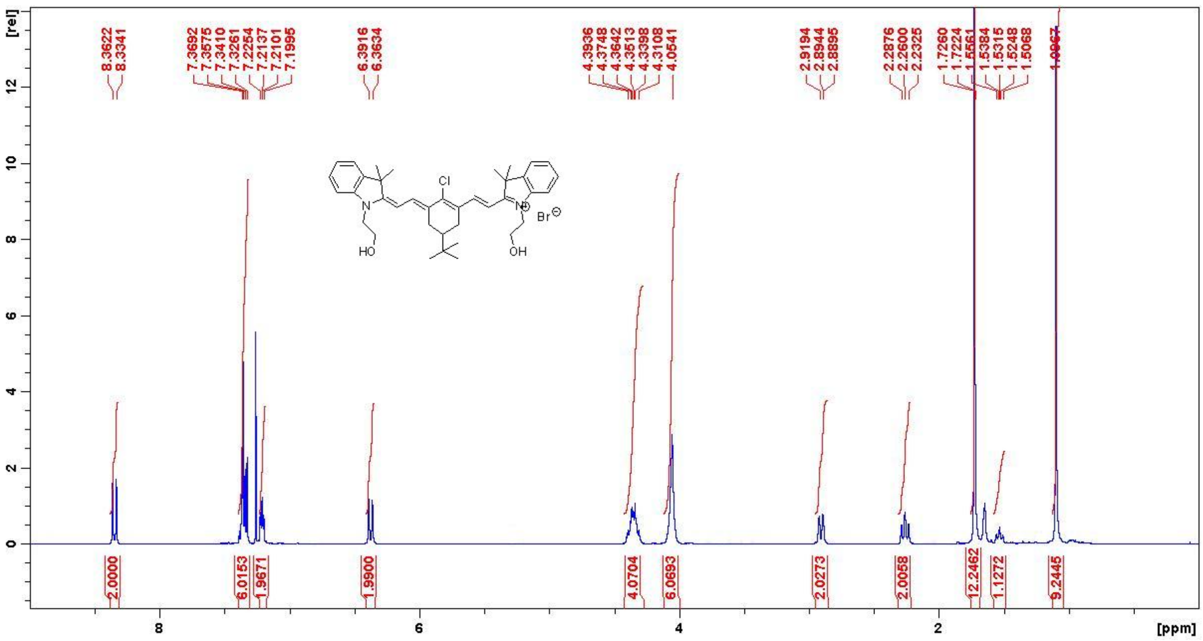
[rel]
8.3622
8.3341
7.3692
7.3575
7.3410
7.3261
7.2254
7.2137
7.2101
7.1995
6.3916
6.3634
4.3936
4.3748
4.3642
4.3513
4.3398
4.3108
4.0541
2.9194
2.8944
2.8895
2.2876
2.2600
2.2325
1.7260
1.7224
1.5561
1.5384
1.5315
1.5248
1.5068
Cl
N
N
Br
HO
OH
2.0000
6.0153
1.9671
1.9900
4.0704
6.0693
2.0273
2.0058
12.2462
1.1272
9.2445
[ppm]

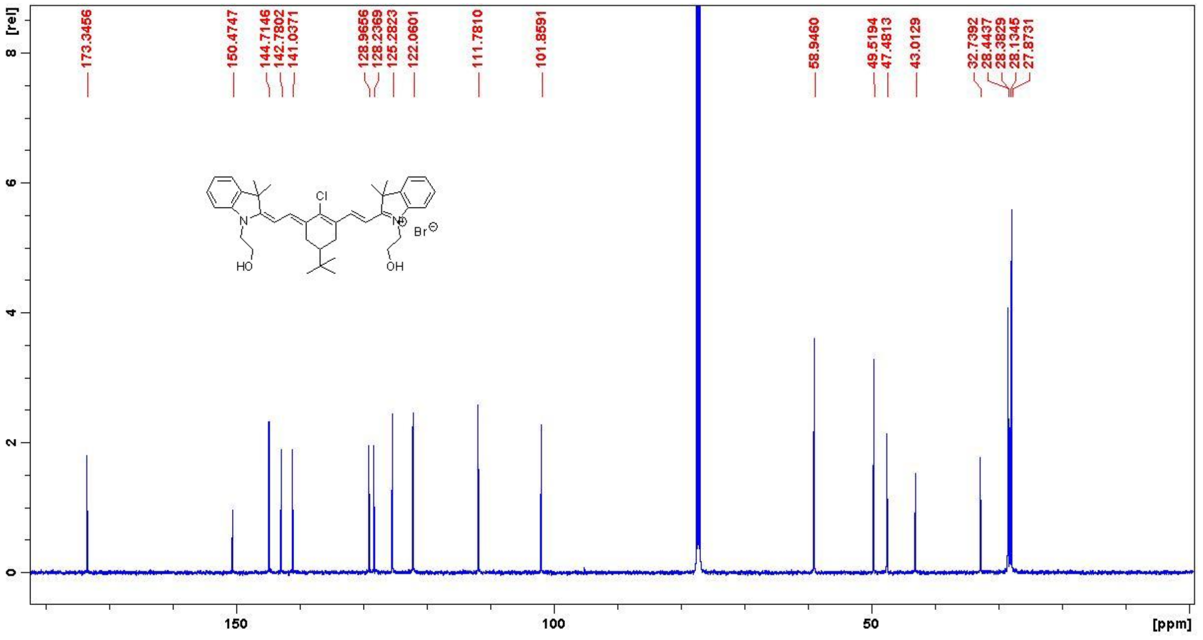
[rel]
173.3456
150.4747
144.7146
142.7802
141.0371
128.9656
128.2369
125.2823
122.0601
111.7810
101.8591
58.9460
49.5194
47.4813
43.0129
32.7392
28.4437
28.3829
28.1345
27.8731
Cl
N
N
Br
HO
OH
[ppm]

Compound D^6D^6

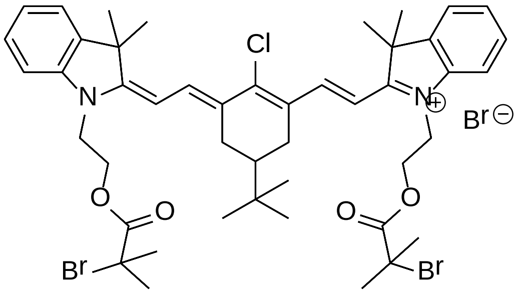

To a solution of 500 mg of $\mathbf{D^4D^4}$ (0.74 mmol, 1 equiv.) in 6 mL of anhydrous DCM at 0 °C were added 1.09 mL of distilled DIEA (6.62 mmol, 9 equiv.) and 0.73 mL of 2-bromoisobutyrylbromide (5.88 mmol, 8 equiv.). The solution was stirred for 15 min at 0 °C and for 1 h at RT. The reaction was quenched with an aqueous solution of HBr 1M. The organic layer was then washed with water, dried over Na_2SO_4 and concentrated. The crude was purified by flash chromatography on silica gel with DCM/MeOH as eluent (from 95:5 to 90:10) to afford a greenish solid in a 87% yield (623 mg).

^{1}H NMR ($CDCl_3$, 500.10 MHz): δ 8.37 (d, ^3J = 13 Hz, 2H, =C**H**), 7.40-7.30 (m, 6H, C$\mathbf{H}_{Ar}$), 7.24 (m, 2H, C$\mathbf{H}_{Ar}$), 6.46 (d, ^3J = 13 Hz, 2H, =C**H**), 4.79 and 4.68 (m, 8H, C$\mathbf{H}_2$), 3.00 (d, ^2J = 12 Hz, 2H, $\mathbf{H}_{eq}$), 2.72 (dd, ^2J = 12 Hz, ^3J = 12 Hz, 2H, $\mathbf{H}_{ax}$), 1.77 (s, 12H, C(C$\mathbf{H}_3$)$_2$), 1.73 (s, 12H, C(C$\mathbf{H}_3$)$_2$), 1.56 (m, 1H, C**H**), 1.12 (s, 9H, C(C$\mathbf{H}_3$)$_3$).

^{13}C NMR ($CDCl_3$, 125.75 MHz): δ 172.9 (C_{quat}), 171.7 (C_{quat}), 150.6 (C_{quat}), 144.5 (CH), 142.3 (C_{quat}), 141.0 (C_{quat}), 129.0 (C_{quat}), 128.9 (CH), 125.6 (CH), 122.3 (CH), 111.4 (CH), 102.3 (CH), 62.4 (CH_2), 55.1 (C_{quat}), 49.6 (C_{quat}), 43.7 (CH_2), 42.8 (CH), 32.7 (C_{quat}), 30.7 (CH_3), 28.3 (CH_3), 28.1 (CH_2), 27.7 (CH_3).

UV-Vis (CH_3OH): λ_{max} = 779 nm (ε_{max} = 214000 L.mol^{-1}.cm^{-1}).

HRMS (ESI+): $[M-Br]^+$ = 895.2408 (calcd for $C_{46}H_{58}Br_2ClN_2O_4^+$: 895.2446).

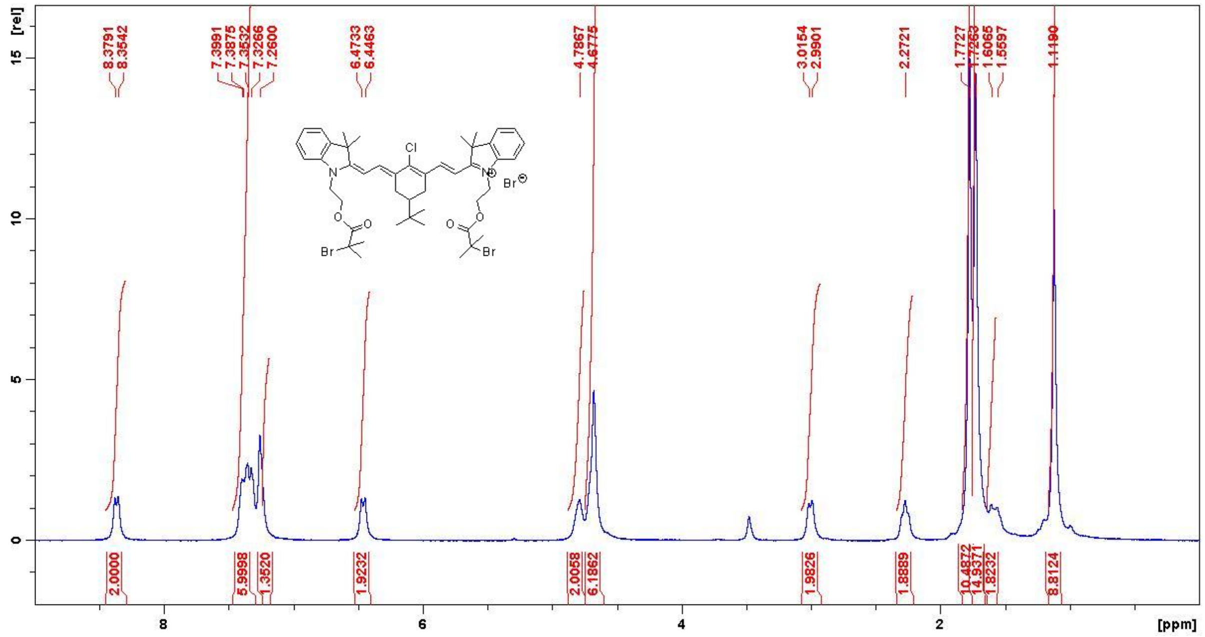

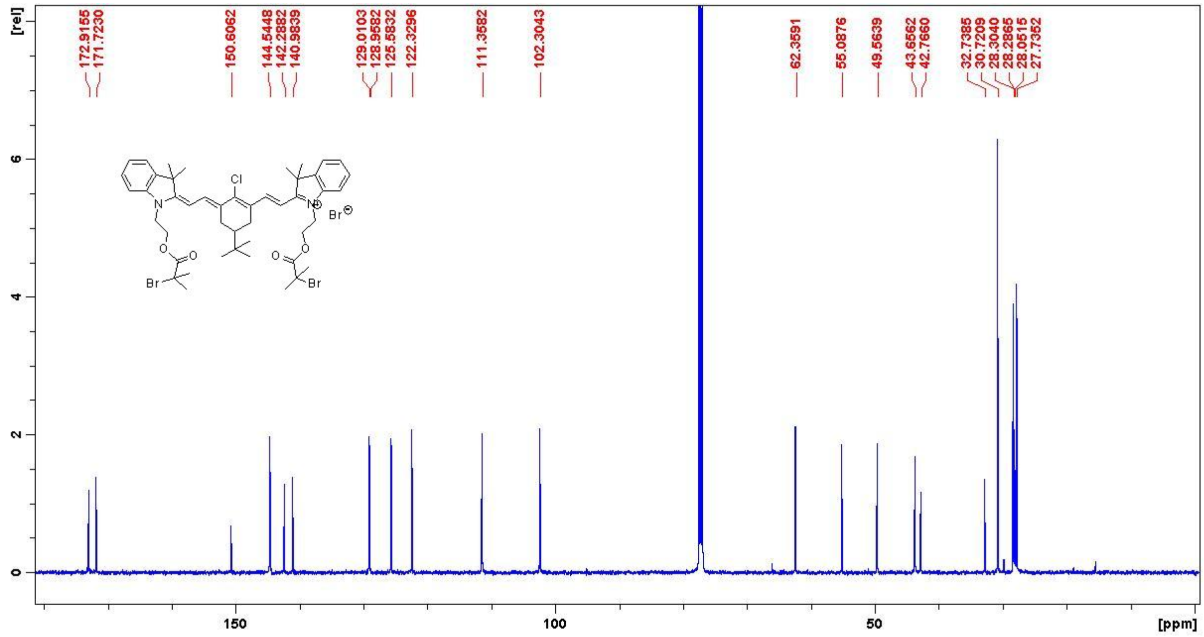

D^6D^6=O

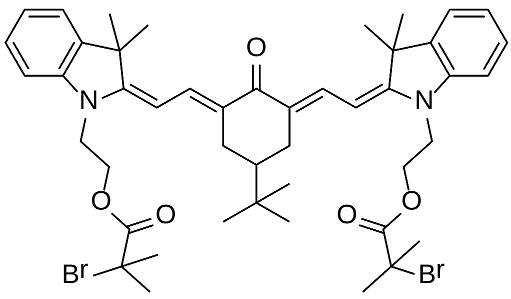

To a solution of 60 mg of **D^6D^6** (0.06 mmol, 1 equiv.) and 11 mg of *N*-hydroxysuccinimide (0.09 mmol, 1.5 equiv.) in 3 mL of anhydrous DMF were added 20 µL of distilled DIEA (0.12 mmol, 2 equiv.) and the mixture was stirred for 3 h at 40 °C. Then the solvent was removed under reduced pressure, the residue was dissolved in DCM, washed with a saturated aqueous solution of NH_4Cl, then with brine, dried over Na_2SO_4 and finally concentrated. The crude residue was submitted to flash chromatography on silica gel using DCM/MeOH (98:2, R_f = 0.90) as eluent. The product was isolated as an orange-reddish solid in 74% yield (40 mg).

^{1}H NMR ($CDCl_3$, 500.10 MHz): δ 8.13 (d, ^3J = 13 Hz, 2H, =C**H**), 7.19 (m, 4H, C**H**$_{Ar}$), 6.93 (t, ^3J = 8 Hz, 2H, C**H**$_{Ar}$), 6.74 (t, ^3J = 8 Hz, 2H, C**H**$_{Ar}$), 5.62 (d, ^3J = 13 Hz, 2H, =C**H**), 4.47 (t, ^3J = 7 Hz, 2H, C**H**$_2$), 4.04 (t, ^3J = 7 Hz, 2H, C**H**$_2$), 2.92 (d, ^2J = 15 Hz, 2H, **H**$_{eq}$), 2.14 (dd, ^2J = 14 Hz, ^3J = 14 Hz, 1H, **H$_{ax}$**), 1.81 (s, 12H, C(C**H**$_3$)$_2$), 1.67 (s, 6H, C(C**H**$_3$)$_2$), 1.67 (s, 6H, C(C**H**$_3$)$_2$), 1.52 (m, 1H, C**H**), 1.06 (s, 9H, C(C**H**$_3$)$_3$).

^{13}C NMR ($CDCl_3$, 125.75 MHz): δ 187.0 (C_{quat}), 171.8 (C_{quat}), 162.0 (C_{quat}), 144.0 (C_{quat}), 139.5 (C_{quat}), 132.5 (CH), 127.8 (CH), 127.6 (C_{quat}), 122.1 (CH), 121.0 (CH), 106.8 (CH), 93.1 (CH), 61.6 (CH_2), 55.4 (C_{quat}), 46.7 (C_{quat}), 43.7 (CH), 40.8 (CH_2), 32.8 (C_{quat}), 30.7 (CH_3), 29.0 (CH_3), 28.9 (CH_3), 27.7 (CH_3), 27.0 (CH_2).

UV-Vis (CH_3OH): λ_{max} = 522 nm (ε_{max} = 58000 L.mol^{-1}.cm^{-1}).

UV-Vis (CH_3OH + CH_3CO_2H): λ_{max} = 707 nm (ε_{max} = 133000 L.mol^{-1}.cm^{-1}).

HRMS (ESI+): $[M+H]^+$ = 877.2762 (calcd for $C_{46}H_{59}Br_2N_2O_5^+$: 877.2785).

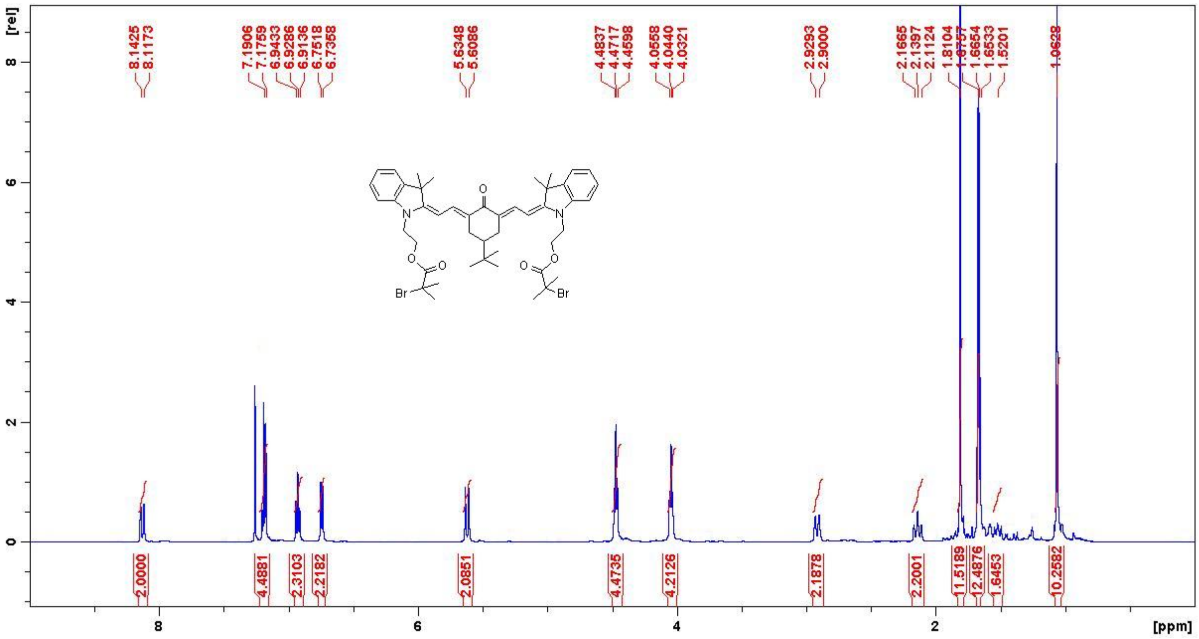

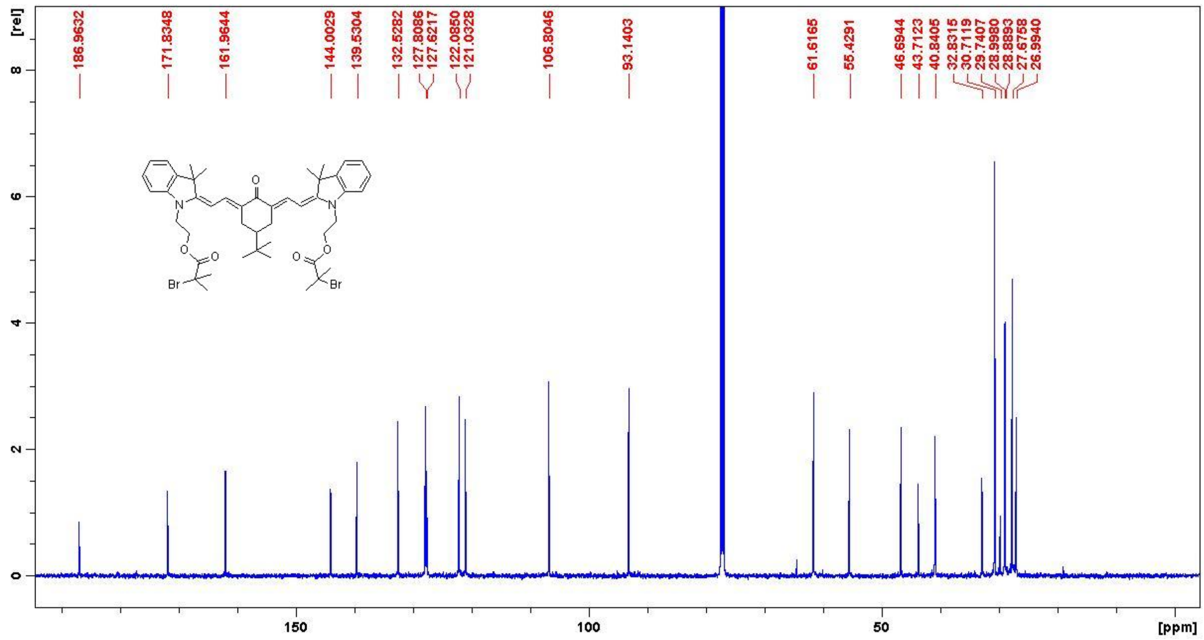

Compound DD^{PHEA}=O

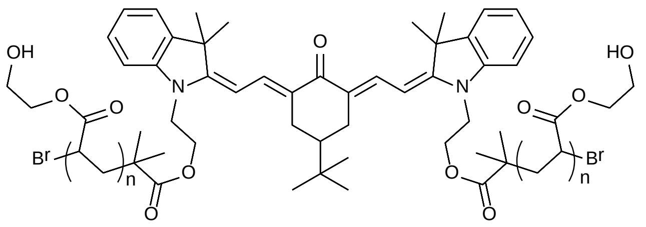

A glass tube was filled with 1.6 mL of 2-hydroxyethyl acrylate (15.24 mmol, 335 equiv.). The solution was degassed using Argon bubbling for 10 min and 40 mg of **D^6D^6=O** (0.05 mmol, 1 equiv.), 13 mg of CuBr (0.09 mmol, 2 equiv.) and 0.28 mg of 2,2'-bipyridine (0.18 mmol, 4 equiv.) were added. The glass tube was sealed and the mixture was stirred for exactly 1 h at 85 °C. The reaction was quenched by addition of distilled water and the crude solution was transferred to a dialysis bag (MWCO = 2000 Da) and dialyzed against a large volume of distilled water (which was replaced at 8H intervals) for 24 hours. The resulting reddish solution was lyophilized, yielding reddish fibers (390 mg).

^{1}H NMR (CD_3OD, 500.10 MHz): δ 8.12 (m, 2H, =C**H**), 7.24 (m, 4H, C**H**$_{Ar}$), 6.94 (m, 4H, C**H**$_{Ar}$), 5.73 (m, 2H, =C**H**), 4.13 (m, C**H**$_2$ PHEA), 3.71 (m, C**H**$_2$ PHEA), 2.40 (m, C**H** PHEA), 1.95-1.58 (m, C**H**$_2$ PHEA), 1.06 (s, 9H, C(C**H**$_3$)$_3$). Unexpected aromatic signals were observed on the ^{1}H NMR. DOSY experiment confirmed that these signals were part of the compound. The remaining aliphatic signals (2 C(C**H**$_3$)$_2$) and C**H**) generally observed *ca.* 1-2 ppm lie under the dominant PHEA signal.

^{13}C NMR (CD_3OD, 125.75 MHz): δ 178.6 (C_{quat}), 177.1 (C_{quat}), 176.3 (C_{quat} PHEA), 167.4 (C_{quat}), 165.2 (C_{quat}), 145.1 (C_{quat}), 140.6 (C_{quat}), 136.1 (=CH), 129.3 (CH), 122.9 (CH), 122.4 (CH), 108.9 (CH), 94.4 (=CH), 67.2 (CH_2 PHEA), 60.9 (CH_2 PHEA), 42.6 (CH PHEA), 35.9 (CH_2 PHA), 29.1 (CH_3), 28.1 (CH_3), 27.9 (CH_2). 1 CH_3, 2 CH_2, 1 CH and 3 C_{quat} signals are hidden between polymer signals in the aliphatic part of the spectrum.

n ~ 80, determined by ^{1}H NMR.

UV-Vis (CH_3OH): λ_{max} = 525 nm.

UV-Vis (H_2O): λ_{max} = 541 nm.

GPC: M_n = 32650 g.mol^{-1}; PDI = 1.33 (GPC); 1.09 (DOSY).

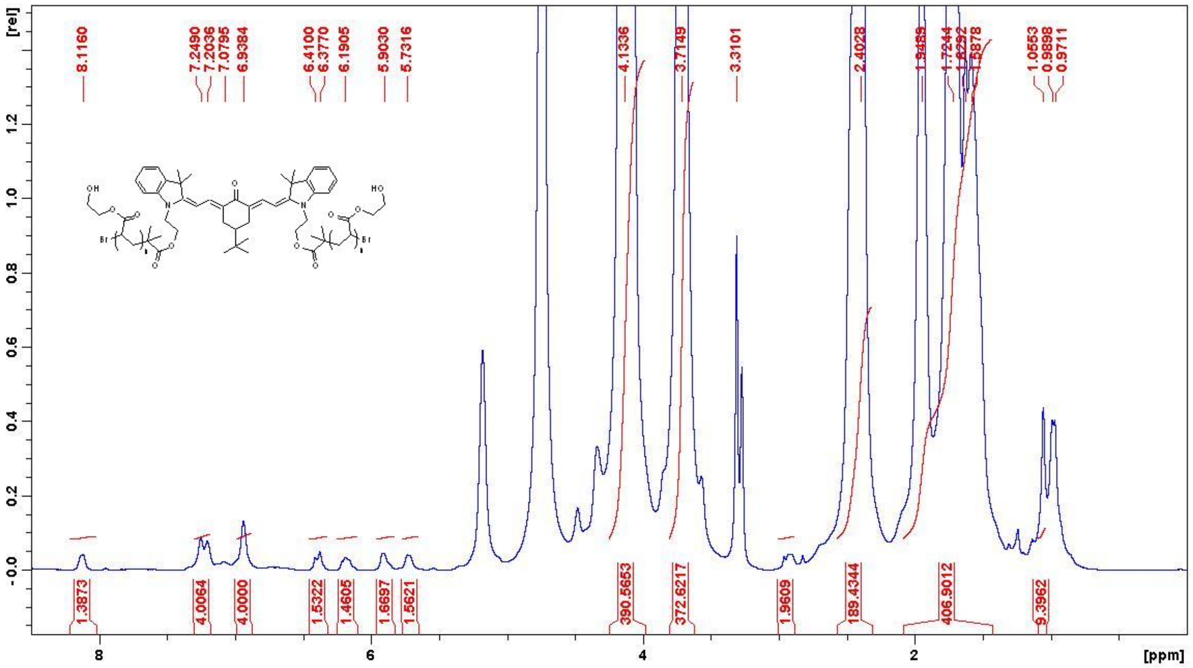
[rel]
8.1160
7.2490
7.2036
7.0795
6.9384
6.4100
6.3770
6.1905
5.9030
5.7316
4.1336
3.7149
3.3101
2.4028
1.7244
1.5878
1.0553
0.9898
0.9711
1.3873
4.0064
4.0000
1.5322
1.4605
1.6697
1.5621
390.5653
372.6217
1.9609
189.4344
406.9012
9.3962
[ppm]

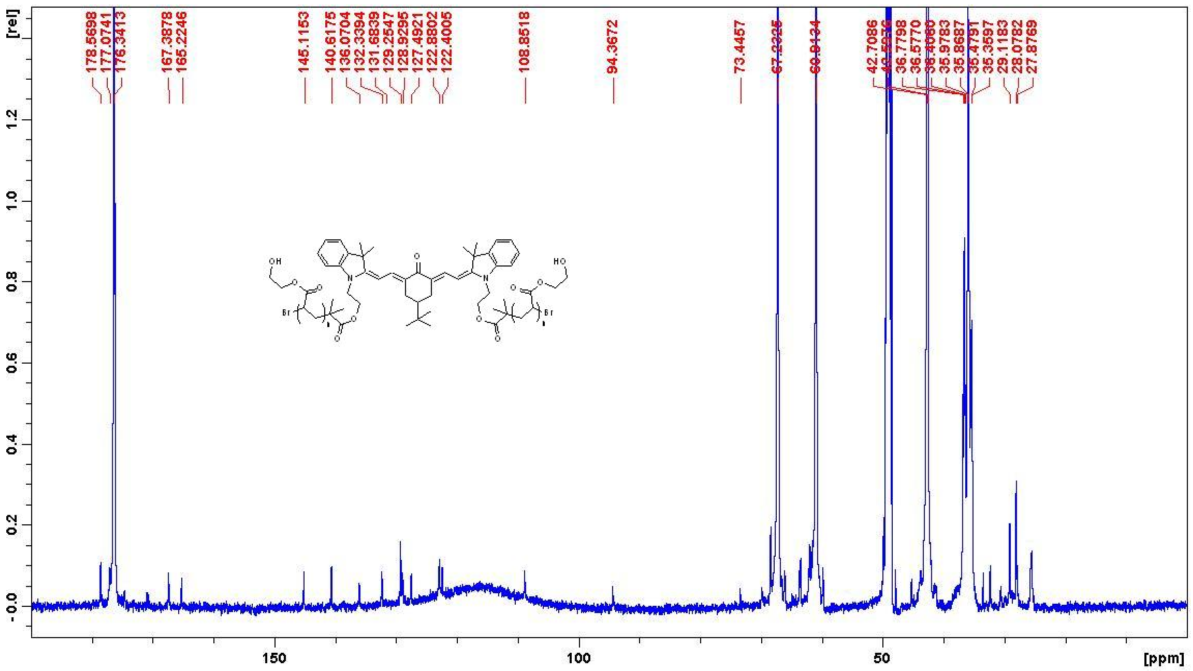
[rel]
178.5698
177.0741
176.3413
167.3878
165.2246
145.1153
140.6175
136.0704
132.3394
131.6839
129.2547
128.9295
127.4921
122.8802
122.4005
108.8518
94.3672
73.4457
42.7086
36.7798
36.5770
35.9783
35.8687
35.4791
35.3597
29.1183
28.0782
27.8769
[ppm]

Compound D^3A=O

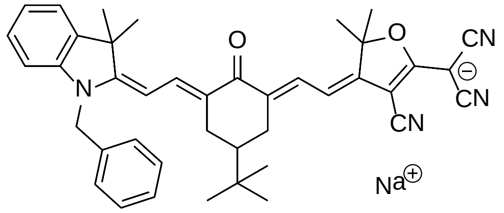

To a solution of 200 mg of **D^3A** (0.31 mmol, 1 equiv.) and 54 mg of *N*-hydroxysuccinimide (0.47 mmol, 2.5 equiv.) in 5 mL of anhydrous DMF was added 0.10 mL of distilled DIEA (0.62 mmol, 2 equiv.) and the mixture was stirred for 2 h at RT. The solvent was evaporated under reduced pressure and the residue was dissolved in 10 mL of MeOH. 234 mg of sodium iodide (1.56 mmol, 5 equiv.) were added and the resulting mixture was stirred for 30 min at RT. The solvent was evaporated and the residue was dissolved in 50 mL of DCM and washed with water. The organic layer was dried over Na_2SO_4 and concentrated. The crude residue was purified by flash chromatography on silica gel with EtOAc/MeOH (98:2, R_f = 0.49) as eluent to afford the target compound as a deep purple solid in a 70% yield (141 mg).

^{1}H NMR (CD_3CN, 500.10 MHz): δ 8.19 (m, 1H, =C**H**), 7.98 (d, ^3J = 12 Hz, 1H, =C**H**), 7.30-7.21 (m, 7H, C**H**$_{Ar}$), 7.00 (t, ^3J = 8 Hz, 1H, C**H**$_{Ar}$), 6.94 (d, ^3J = 8 Hz, 1H, C**H**$_{Ar}$), 5.66 (d, ^3J = 12 Hz, 1H, =C**H**), 5.52 (d, ^3J = 12 Hz, 1H, =C**H**), 5.06 (d, ^2J = 17 Hz, 2H, C**H**$_2$), 4.96 (d, ^2J = 17 Hz, 2H, C**H**$_2$), 2.75 (d, ^2J = 15 Hz, 1H, **H**$_{eq}$), 2.51 (d, ^2J = 15 Hz, 1H, **H**$_{eq}$), 1.84 (dd, ^2J = 14 Hz, ^3J = 14 Hz, 1H, **H**$_{ax}$), 1.58 (m, 1H, **H**$_{ax}$), 1.58 (s, 3H, C(C**H**$_3$)$_2$), 1.55 (s, 3H, C(C**H**$_3$)$_2$), 1.50 (s, 6H, C(C**H**$_3$)$_2$), 1.28 (m, 1H, C**H**), 0.95 (s, 9H, C(C**H**$_3$)$_3$).

^{13}C NMR ($CDCl_3$, 125.75 MHz): Compound was too insoluble to record a ^{13}C spectrum.

UV-Vis (CH_3OH): λ_{max} = 549 nm (ε_{max} = 66000 L.mol^{-1}.cm^{-1}).

UV-Vis (CH_3OH + CH_3CO_2H): λ_{max} = 757 nm (ε_{max} = 160000 L.mol^{-1}.cm^{-1}).

HRMS (ESI-): [M−Na]$^-$ = 621.3218 (calcd for $C_{41}H_{41}N_4O_2^-$: 621.3235).

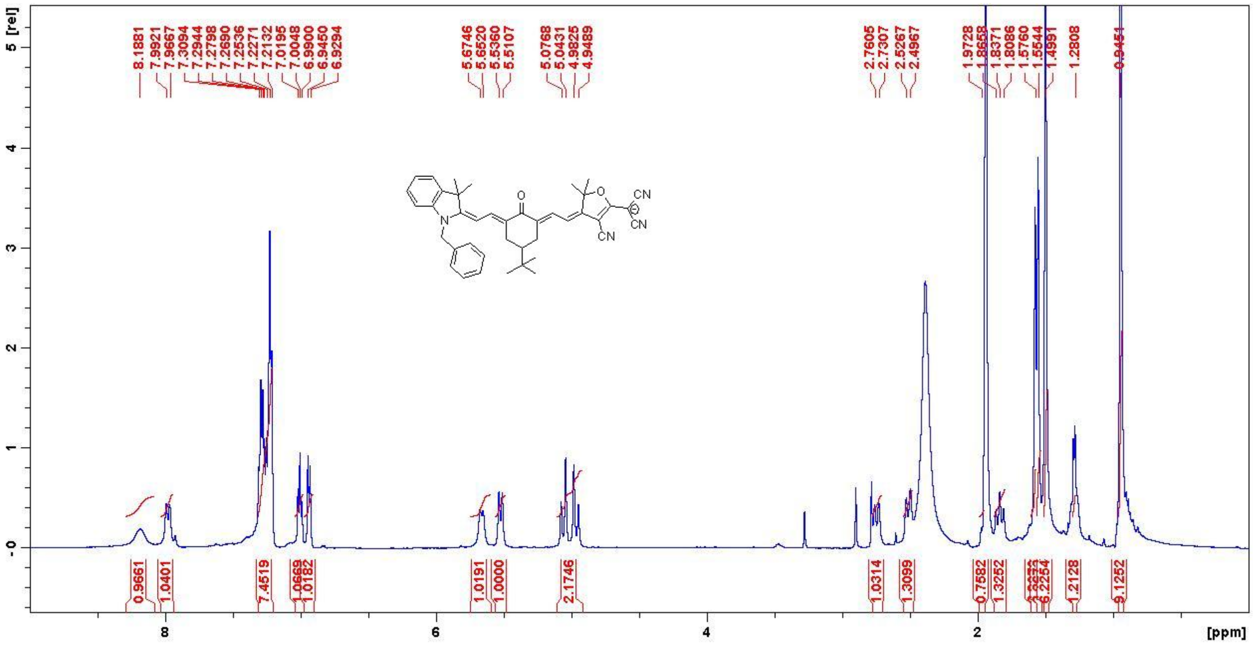
5 [rel]
8.1881
7.9921
7.9667
7.3094
7.2944
7.2798
7.2690
7.2536
7.2271
7.2132
7.0195
7.0048
6.9900
6.9450
6.9294
5.6746
5.6520
5.5360
5.5107
5.0768
5.0431
4.9825
4.9489
2.7605
2.7307
2.5267
2.4967
1.9728
1.8658
1.8371
1.8086
1.5760
1.5544
1.4991
1.2808
0.9451
CN
CN
CN
O
N
0.9661
1.0401
7.4519
1.0669
1.0182
1.0191
1.0000
2.1746
1.0314
1.3099
0.7582
1.3252
6.2254
1.2128
9.1252
[ppm]

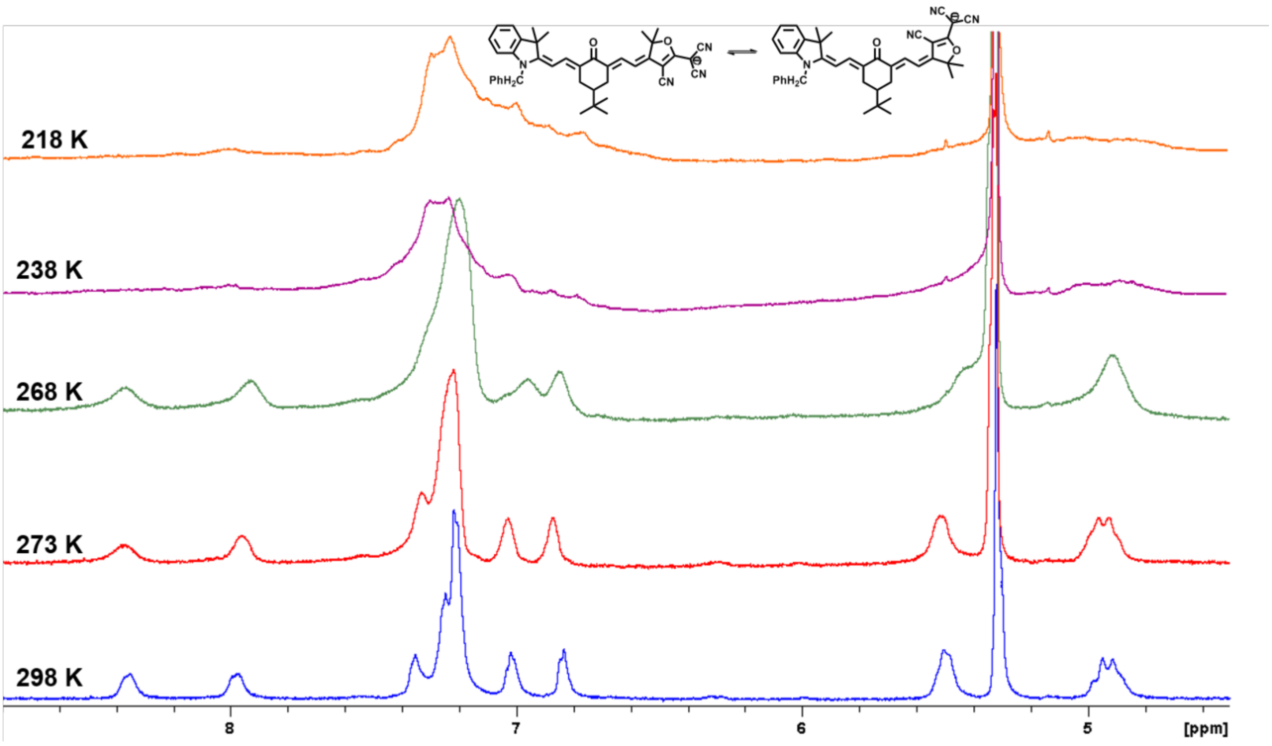
218 K
238 K
268 K
273 K
298 K
PhH2C
CN
CN
NC
NC
CN
[ppm]

Compound D^2A

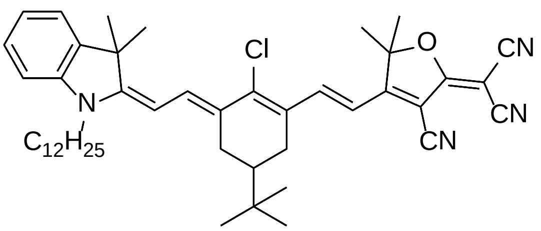

To a solution of 200 mg of precursor **2** (0.46 mmol, 1 equiv.) and 224 mg of $\mathbf{D^2}$ (0.55 mmol, 1.2 equiv.) in 30 mL of absolute ethanol were added 60 µL of distilled pyridine (0.68 mmol, 1.5 equiv.). The reaction was stirred for 16 h at 80 °C. The solution was evaporated and the residue was dissolved in 20 mL of DCM and washed with 20 mL of water. The organic layer was dried over Na_2SO_4 and concentrated. The crude solid was purified by flash chromatography on silica gel, using DCM as eluent (R_f = 0.20) to afford the product as a greenish solid in a 74% yield (244 mg).

^{1}H NMR ($CDCl_3$, 500.10 MHz): δ 8.12 (d, ^3J = 15 Hz, 1H, =C**H**), 7.95 (d, ^3J = 13 Hz, 1H, =C**H**), 7.29-7.26 (m, 2H, C$\mathbf{H}_{Ar}$), 7.06 (t, ^3J = 8 Hz, 1H, C$\mathbf{H}_{Ar}$), 6.85 (d, ^3J = 8 Hz, 1H, C$\mathbf{H}_{Ar}$), 6.19 (d, ^3J = 15 Hz, 1H, =C**H**), 5.70 (d, ^3J = 13 Hz, 1H, =C**H**), 3.79 (m, 2H, N-C$\mathbf{H}_2$), 2.86 (d, ^2J = 14 Hz, 1H, $\mathbf{H}_{eq}$), 2.74 (d, ^2J = 14 Hz, 1H, $\mathbf{H}_{eq}$), 2.13 (dd, ^2J = 13 Hz, ^3J = 13 Hz, 1H, $\mathbf{H}_{ax}$), 2.06 (dd, ^2J = 13 Hz, ^3J = 13 Hz, 1H, $\mathbf{H}_{ax}$), 1.76 (s, 6H, C(C$\mathbf{H}_3$)$_2$), 1.75 (m, 2H, CH_2), 1.66 (s, 6H, C(C$\mathbf{H}_3$)$_2$), 1.63 (m, 2H, C$\mathbf{H}_2$), 1.55 (m, 1H, C**H**), 1.39 (m, 4H, C$\mathbf{H}_2$), 1.29 (m, 4H, C$\mathbf{H}_2$), 1.25 (m, 8H, C$\mathbf{H}_2$), 1.04 (s, 9H, C(C$\mathbf{H}_3$)$_3$), 0.88 (t, ^3J = 7 Hz, 3H, C$\mathbf{H}_3$).

^{13}C NMR ($CDCl_3$, 125.75 MHz): δ 176.4 (C_{quat}), 173.1 (C_{quat}), 165.8 (C_{quat}), 147.4 (C_{quat}), 144.4 (CH), 143.3 (C_{quat}), 140.0 (C_{quat}), 136.8 (CH), 128.4 (CH), 127.7 (C_{quat}), 126.0 (C_{quat}), 122.7 (CH), 122.2 (CH), 113.5 (C_{quat}), 112.6 (C_{quat}), 111.9 (C_{quat}), 110.6 (CH), 108.4 (CH), 99.7 (C_{quat}), 96.5 (CH), 93.3 (C_{quat}), 53.4 (C_{quat}), 47.7 (C_{quat}), 43.4 (N-CH_2), 42.5 (CH), 32.5 (C_{quat}), 32.0 (CH_2), 29.7 (2 CH_2), 29.6 (2 CH_2), 29.5 (2 CH_2), 28.4 (CH_3), 27.7 (CH_2), 27.5 (CH_3), 27.3 (CH_2), 27.2 (CH_2), 27.2 (CH_3), 24.6 (CH_2), 22.8 (CH_2), 14.2 (CH_3).

UV-Vis (CH_2Cl_2): λ_{max} = 832 nm (ε_{max} = 136000 L.mol^{-1}.cm^{-1}).

HRMS (ESI+): $[M+Na]^+$ = 741.4237 (calcd for $C_{46}H_{59}ClN_4NaO^+$: 741.4270).

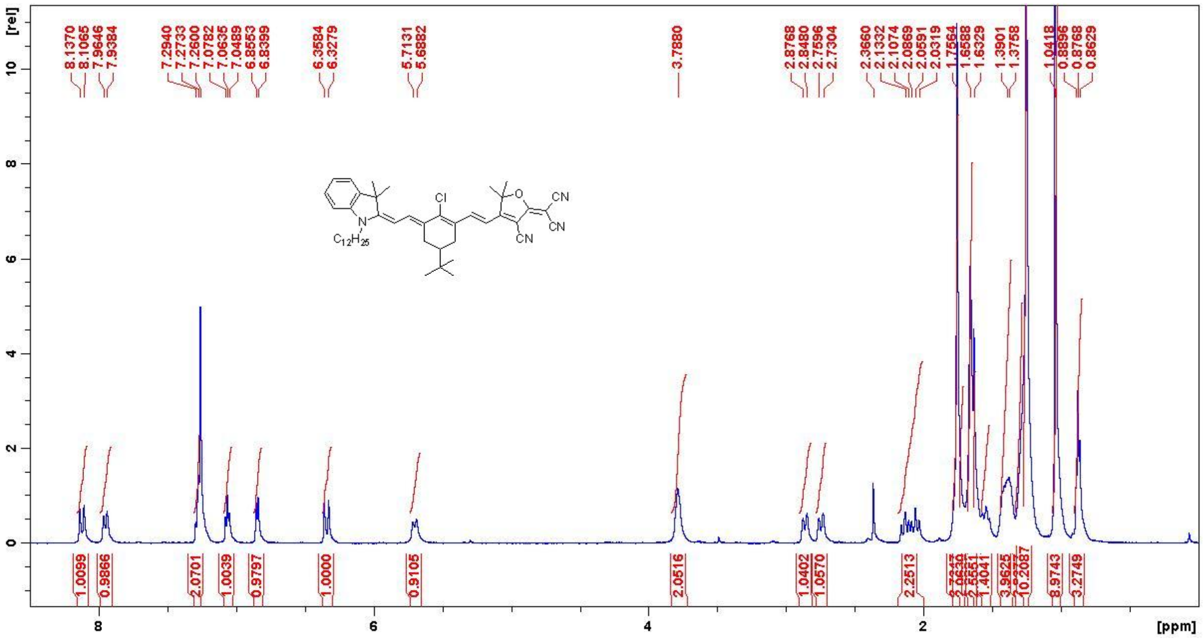
[rel]
8.1370
8.1065
7.9646
7.9384
7.2940
7.2733
7.2600
7.0782
7.0635
7.0489
6.8553
6.8399
6.3584
6.3279
5.7131
5.6882
3.7880
2.8768
2.8480
2.7596
2.7304
2.3660
2.1332
2.1074
2.0869
2.0591
2.0319
1.6588
1.6329
1.3901
1.3758
1.0418
0.8896
0.8768
0.8629
Cl
CN
CN
CN
N
$C_{12}H_{25}$
1.0099
0.9866
2.0701
1.0039
0.9797
1.0000
0.9105
2.0516
1.0402
1.0570
2.2513
1.4041
3.9625
10.2087
8.9743
3.2749
[ppm]

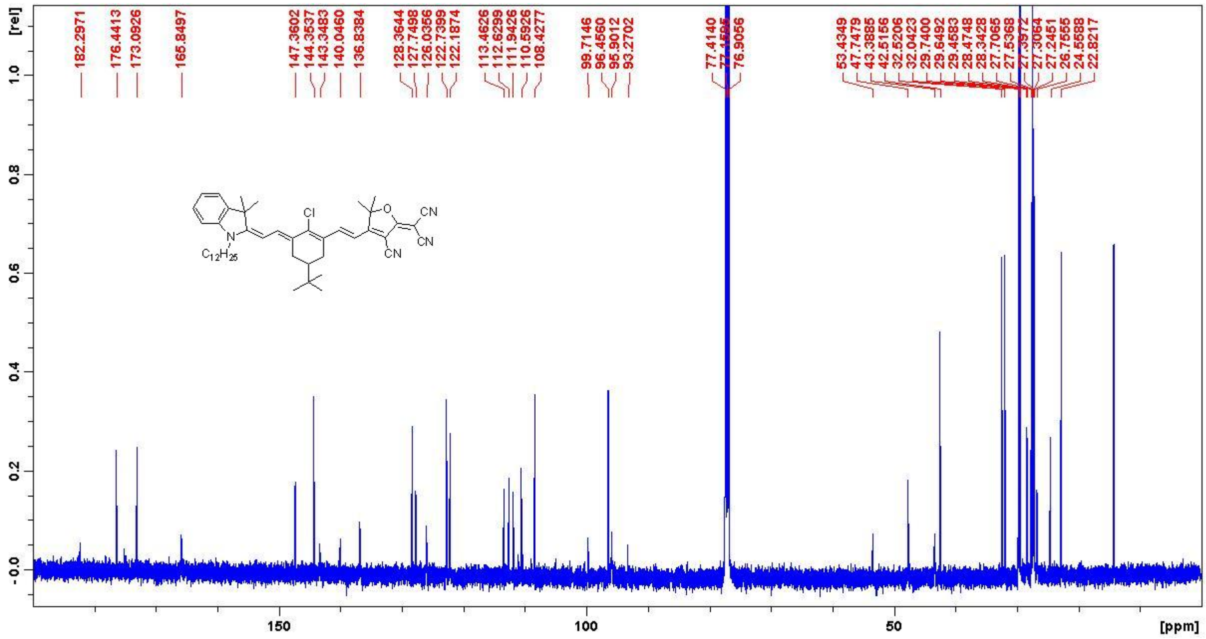
[rel]
182.2971
176.4413
173.0926
165.8497
147.3602
144.3537
143.3483
140.0460
136.8384
128.3644
127.7498
126.0356
122.7399
122.1874
113.4626
112.6299
111.9426
110.5926
108.4277
99.7146
96.4560
95.9012
93.2702
77.4140
76.9056
53.4349
47.7479
43.3885
42.5156
32.5206
32.0423
29.7400
29.6492
29.4583
28.4748
28.3428
27.7065
27.5368
27.3064
27.2451
26.7555
24.5588
22.8217
Cl
CN
CN
CN
N
$C_{12}H_{25}$
[ppm]

Compound D^2A=O

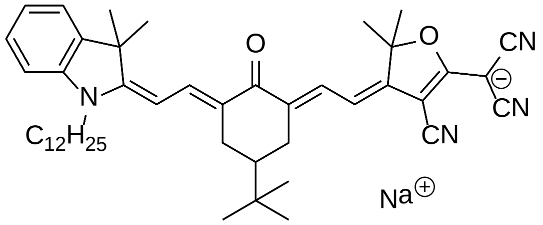

To a solution of 150 mg of **D^2A** (0.21 mmol, 1 equiv.) and 48 mg of *N*-hydroxysuccinimide (0.42 mmol, 2 equiv.) in 3 mL of anhydrous DMF were added 90 µL of distilled DIEA (0.52 mmol, 2.5 equiv.) and the mixture was stirred for 3 h at 40 °C. The solvent was evaporated under reduced pressure and the residue was dissolved in DCM, washed with water and brine. The organic layer was dried over Na_2SO_4 and concentrated. The crude solid was purified by flash chromatography on silica gel with DCM/MeOH (95:5, R_f = 0.26) as eluent to afford product as a deep purple solid in 62% yield (94 mg).

^{1}H NMR (CD_3CN, 500.10 MHz): δ 8.05 (d, ^3J = 13 Hz, 2H, =C**H**), 7.31 (d, ^3J = 7 Hz, 1H, C$\mathbf{H}_{Ar}$), 7.25 (dd, ^3J = 8 Hz, ^3J = 7 Hz, 1H, C$\mathbf{H}_{Ar}$), 7.01 (dd, ^3J = 8 Hz, ^3J = 7 Hz, 1H, C$\mathbf{H}_{Ar}$), 6.95 (d, ^3J = 8 Hz, 1H, C$\mathbf{H}_{Ar}$), 5.72 (d, ^3J = 13 Hz, 2H, =C**H**), 3.84 (t, ^3J = 7 Hz, 2H, N-C$\mathbf{H}_2$), 2.79 (m, 2H, $\mathbf{H}_{eq}$), 2.07 (m, 2H, $\mathbf{H}_{ax}$), 1.71 (m, 2H, C$\mathbf{H}_2$), 1.60 (s, 6H, C(C$\mathbf{H}_3$)$_2$), 1.54 (s, 7H, CH and C(C$\mathbf{H}_3$)$_2$), 1.38 (m, 6H, C$\mathbf{H}_2$), 1.25 (m, 12H, C$\mathbf{H}_2$), 1.03 (s, 9H, C(C$\mathbf{H}_3$)$_3$), 0.87 (t, ^3J = 7 Hz, 3H, C$\mathbf{H}_3$).

^{13}C NMR (CD_3CN, 125.75 MHz): Compound was too insoluble to record a ^{13}C spectrum.

UV-Vis (CH_3OH): λ_{max} = 552 nm (ε_{max} = 67000 L.mol^{-1}.cm^{-1}).

HRMS (ESI-): [M−Na]$^-$ = 699.4621 (calcd for $C_{46}H_{59}N_4O_2^-$: 699.4644).

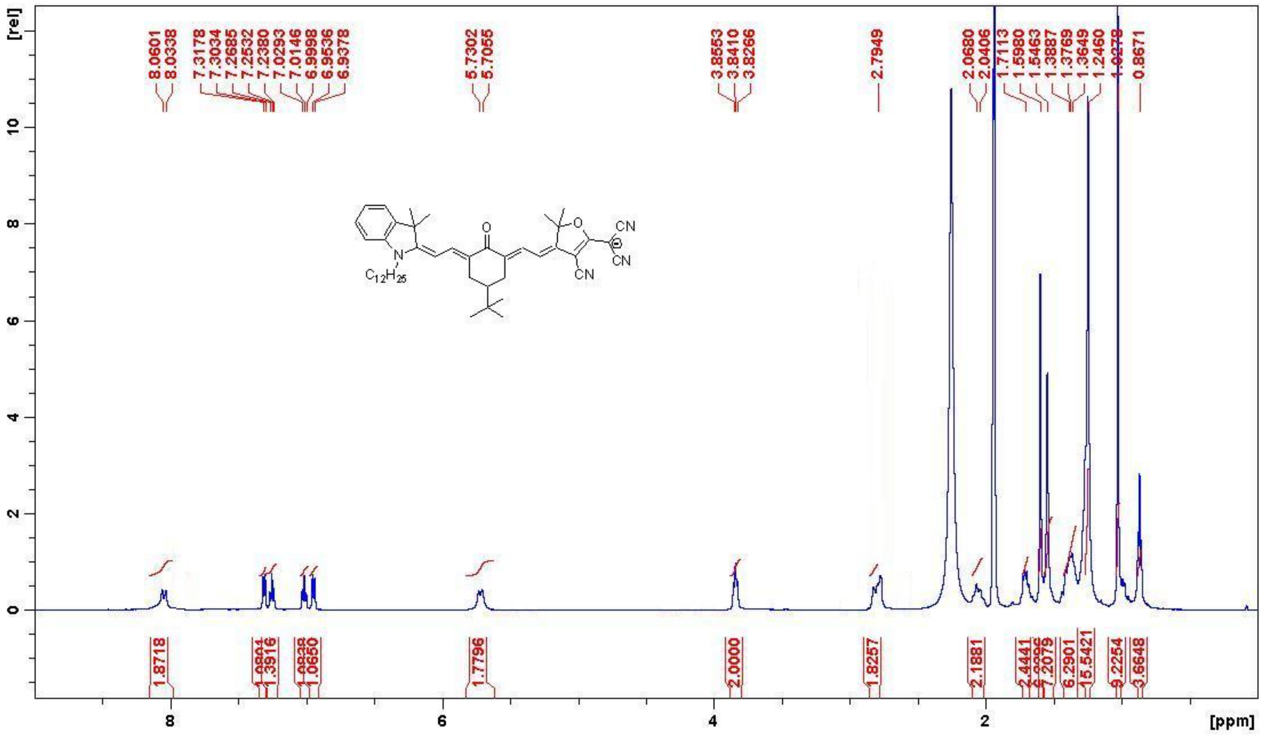

[rel]
[ppm]
8.0601
8.0338
7.3178
7.3034
7.2685
7.2532
7.2380
7.0293
7.0146
6.9998
6.9536
6.9378
5.7302
5.7055
3.8553
3.8410
3.8266
2.7949
2.0680
2.0406
1.7113
1.5980
1.5463
1.3887
1.3769
1.3649
1.2460
0.8671
1.8718
1.3916
1.0650
1.7796
2.0000
1.8257
2.1881
2.4441
7.2079
6.2901
15.5421
3.6648
C12H25
CN

Compound D^5A

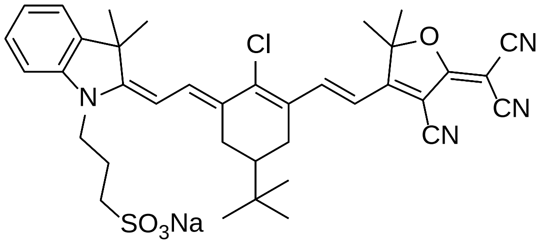

To a solution of 50 mg of precursor **2** (0.11 mmol, 1 equiv.) and 40 mg of $\mathbf{D^5}$ (0.14 mmol, 1.2 equiv.) in 6 mL of absolute ethanol were added 40 µL of distilled pyridine (0.51 mmol, 1.5 equiv.). The reaction was stirred for 17 h at 80 °C. The solution was evaporated and the residue was purified by flash chromatography on silica gel, using $CH_3CN/MeOH/H_2O$ as eluent (90:5:5) to afford the product as a greenish solid in 97% yield (75 mg).

^1H NMR (DMSO-d_6, 500.10 MHz): δ 8.29 (d, ^3J = 14 Hz, 1H, =C**H**), 8.24 (d, ^3J = 14 Hz, 1H, =C**H**), 7.62 (d, ^3J = 7 Hz, 1H, C$\mathbf{H}_{Ar}$), 7.53 (d, ^3J = 8 Hz, 1H, C$\mathbf{H}_{Ar}$), 7.43 (t, ^3J = 8 Hz, 1H, C$\mathbf{H}_{Ar}$), 7.29 (t, ^3J = 7 Hz, 1H, C$\mathbf{H}_{Ar}$), 6.67 (d, ^3J = 14 Hz, 1H, =C**H**), 6.08 (d, ^3J = 14 Hz, 1H, =C**H**), 4.50 (dt, ^2J = 14 Hz, ^3J = 7 Hz, 1H, C$\mathbf{H}_2$), 4.39 (dt, ^2J = 14 Hz, ^3J = 7 Hz, 1H, C$\mathbf{H}_2$), 2.98 (d, ^2J = 15 Hz, 1H, $\mathbf{H}_{eq}$), 2.85 (d, ^2J = 15 Hz, 1H, $\mathbf{H}_{eq}$), 2.53 (t, ^3J = 6 Hz, 2H, C$\mathbf{H}_2$), 2.17 (m, 1H, $\mathbf{H}_{ax}$), 2.04 (m, 1H, $\mathbf{H}_{ax}$), 2.03 (m, 2H, C$\mathbf{H}_2$), 1.66 (s, 6H, C(C$\mathbf{H}_3$)$_2$), 1.59 (s, 6H, C(C$\mathbf{H}_3$)$_2$), 1.41 (m, 1H, C**H**), 1.04 (s, 9H, C(C$\mathbf{H}_3$)$_3$).

^{13}C NMR (DMSO-d_6, 125.75 MHz): δ 176.7 (C_{quat}), 172.9 (C_{quat}), 166.9 (C_{quat}), 147.2 (C_{quat}), 143.5 (CH), 142.0 (C_{quat}), 141.4 (C_{quat}), 139.9 (CH), 128.6 (CH), 128.0 (C_{quat}), 127.5. (C_{quat}), 125.3 (CH), 122.5 (CH), 115.4 (C_{quat}), 114.8 (C_{quat}), 114.6 (C_{quat}), 111.8 (CH), 106.0 (CH), 103.1 (CH), 95.3 (C_{quat}), 49.1 (C_{quat}), 47.6 (CH_2), 44.3 (C_{quat}), 43.0 (CH_2), 42.5 (CH), 32.5 (C_{quat}), 27.6 (CH_2), 27.5 (CH_2), 27.3 (CH_3), 27.3 (CH_3), 26.4 (CH_3), 23.6 (CH_2).

UV-Vis (CH_3CN): λ_{max} = 838 nm (ε_{max} = 126000 L.mol^{-1}.cm^{-1}).

HRMS (ESI-): $[M-Na]^-$ = 671.2491 (calcd for $C_{37}H_{40}ClN_4O_4S^-$: 671.2464).

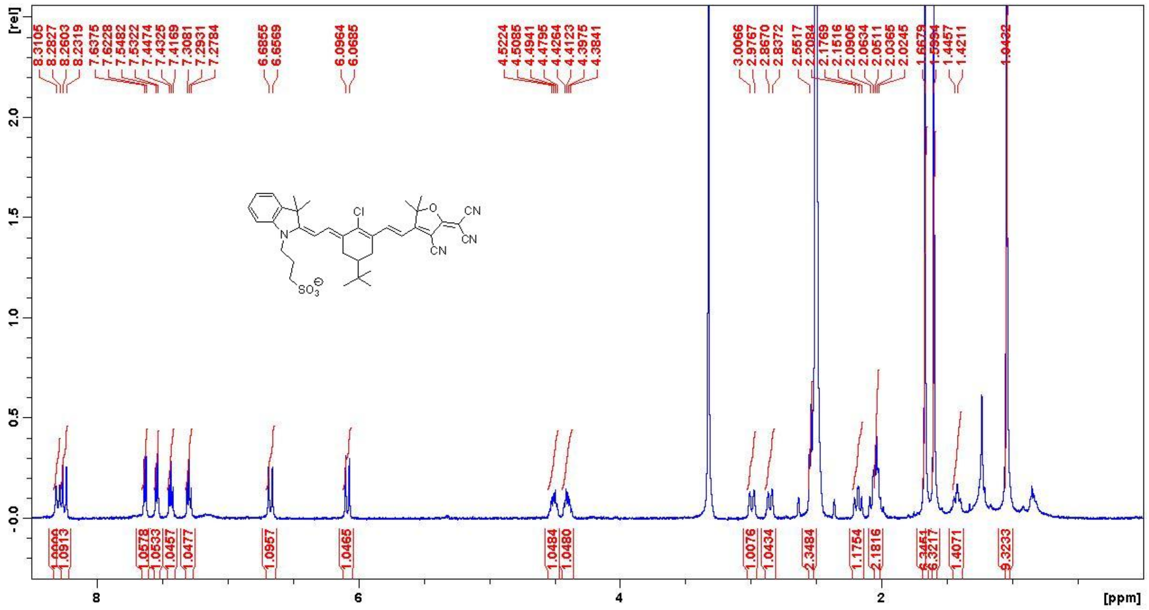

8.3105 8.2827 8.2603 8.2319 7.6375 7.6228 7.5482 7.5322 7.4474 7.4325 7.4169 7.3081 7.2931 7.2784 6.6855 6.6569 6.0964 6.0685 4.5224 4.5085 4.4941 4.4795 4.4264 4.4123 4.3975 4.3841 3.0066 2.9767 2.8670 2.8372 2.5517 2.2084 2.1769 2.1516 2.0905 2.0634 2.0511 2.0365 2.0245 1.6579 1.5994 1.4457 1.4211 1.0432
1.0913 1.0578 1.0533 1.0457 1.0477 1.0957 1.0465 1.0484 1.0480 1.0076 1.0434 2.3484 1.1754 2.1816 6.3217 1.4071 9.3233
[rel]
[ppm]
Cl
N
O
CN
SO3

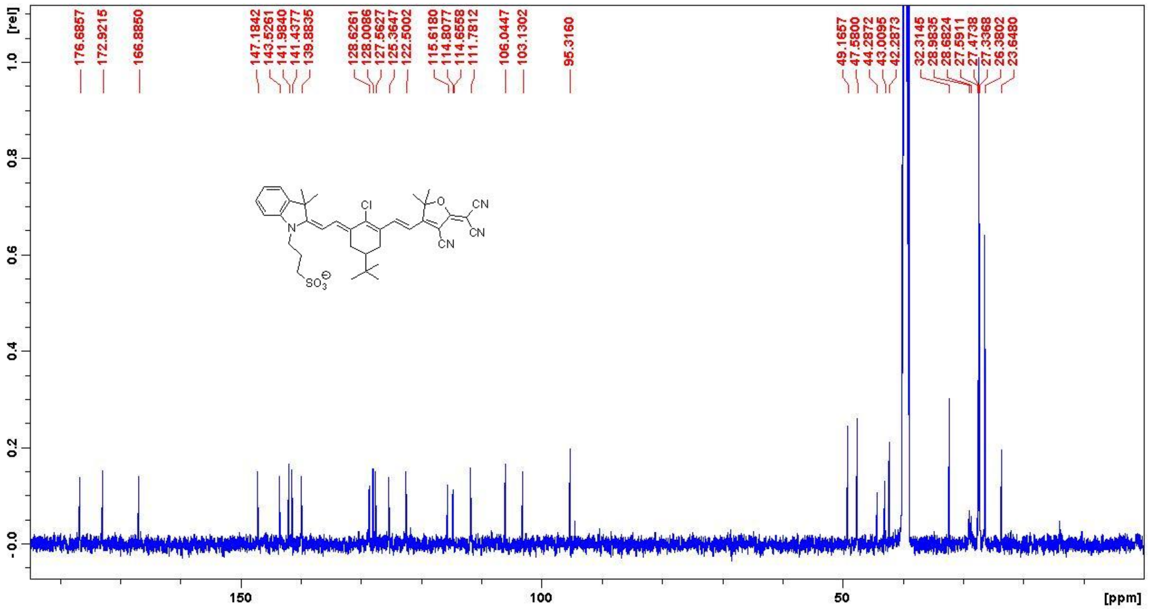

176.6857 172.9215 166.8850 147.1842 143.5261 141.9840 141.4377 139.8835 128.6261 128.0086 127.5627 125.3647 122.5002 115.6180 114.8077 114.6558 111.7812 106.0447 103.1302 95.3160 49.1657 47.5800 44.2872 43.0095 42.2873 32.3145 28.9835 28.6824 27.5911 27.4738 27.3368 26.3802 23.6480
[rel]
[ppm]
Cl
N
O
CN
SO3

Compound D^5A=O

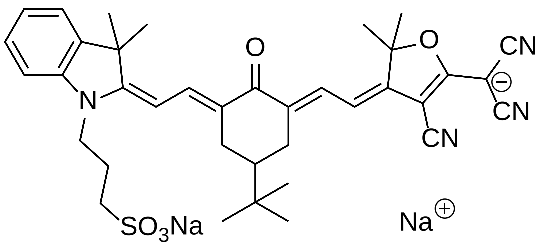

To a solution of 70 mg of **D^5A** (0.10 mmol, 1 equiv.) and 18 mg of *N*-hydroxysuccinimide (0.16 mmol, 1.5 equiv.) in 2 mL of anhydrous DMF were added 30 µL of distilled DIEA (0.21 mmol, 2 equiv.) and the mixture was stirred for 2 h at RT. Then the solvent was removed under reduced pressure and the crude residue was submitted to flash chromatography on silica gel using DCM/MeOH (85:15, R_f = 0.21) as eluent. The product was isolated as deep purple solid in 54% yield (37 mg).

^{1}H NMR (DMSO-d6, 500.10 MHz): δ 8.03 and 7.91 (d, ^3J = 13 Hz, 1H, =C**H**), 7.86 and 7.42 (d, ^3J = 13 Hz, 1H, =C**H**), 7.32 (m, 1H, C**H**$_{Ar}$), 7.18 (m, 1H, C**H**$_{Ar}$), 6.97 (t, ^3J = 7 Hz, 1H, C**H**$_{Ar}$), 6.90 (m, 1H, C**H**$_{Ar}$), 5.62 (m, 1H, =C**H**), 5.58 and 5.43 (m and d, ^3J = 13 Hz, 1H, =C**H**), 3.92 (t, ^3J = 7 Hz, 1H, C**H**$_2$), 2.86-2.72 (m, 2H, **H**$_{ax}$), 2.09-1.90 (m, 4H, C**H**$_2$ and **H**$_{ax}$), 1.57, 1.55 and 1.41 (s, 12H, C(C**H**$_3$)$_2$), 1.31 (m, 1H, C**H**), 1.00 (s, 9H, C(C**H**$_3$)$_3$). HSQC revealed a CH_2 signal under DMSO peak.

^{13}C NMR (DMSO-d6, 125.75 MHz): δ 185.1 (C_{quat}), 184.9 (C_{quat}), 175.1 (C_{quat}), 173.1 (C_{quat}), 162.1 (C_{quat}), 161.7 (C_{quat}), 156.3 (C_{quat}), 154.3 (C_{quat}), 143.9 (C_{quat}), 143.9 (C_{quat}), 138.9 (C_{quat}), 138.8 (C_{quat}), 132.8 (CH), 132.2 (CH), 130.4 (CH), 129.6 (CH), 129.0 (CH), 127.8 (CH), 127.8 (CH), 127.8 (CH), 126.0 (C_{quat}), 125.6 (C_{quat}), 121.7 (CH), 120.5 (CH), 120.4 (CH), 118.2 (C_{quat}), 117.6 (C_{quat}), 115.7 (C_{quat}), 107.5 (CH), 107.4 (CH), 100.4 (CH), 99.6 (CH), 92.3 (CH), 92.3 (C_{quat}), 91.6 (C_{quat}), 91.2 (C_{quat}), 73.3 (C_{quat}), 69.7 (C_{quat}), 56.0 (C_{quat}), 48.6 (C_{quat}), 48.5 (CH), 48.5 (CH), 46.0 (C_{quat}), 45.9 (C_{quat}), 43.5 (CH), 43.4 (CH), 40.8 (broad, CH_2), 35.7 (C_{quat}), 35.6 (C_{quat}), 32.4 (C_{quat}), 32.3 (C_{quat}), 28.3 (CH_3), 28.2 (CH_3), 27.7 (CH_3), 27.7 (CH_3), 27.4 (CH_3), 27.3 (CH_3), 27.1 (CH_3), 26.8 (CH_2), 26.5 (CH_2), 26.5 (CH_2), 26.3 (CH_2), 22.3 (CH_2), 22.3 (CH_2). Two distinct signals were observed for each carbon atom.

UV-Vis (H_2O): λ_{max} = 574 nm (ε_{max} = 35000 L.mol^{-1}.cm^{-1}).

UV-Vis (CH_3OH): λ_{max} = 551 nm (ε_{max} = 67000 L.mol^{-1}.cm^{-1}).

UV-Vis (CH_3OH + CH_3CO_2H): λ_{max} = 757 nm (ε_{max} = 164000 L.mol^{-1}.cm^{-1}).

HRMS (ESI-): $[M+H-2Na]^-$ = 653.2803 (calcd for $C_{37}H_{41}N_4O_5S^-$: 653.2803).

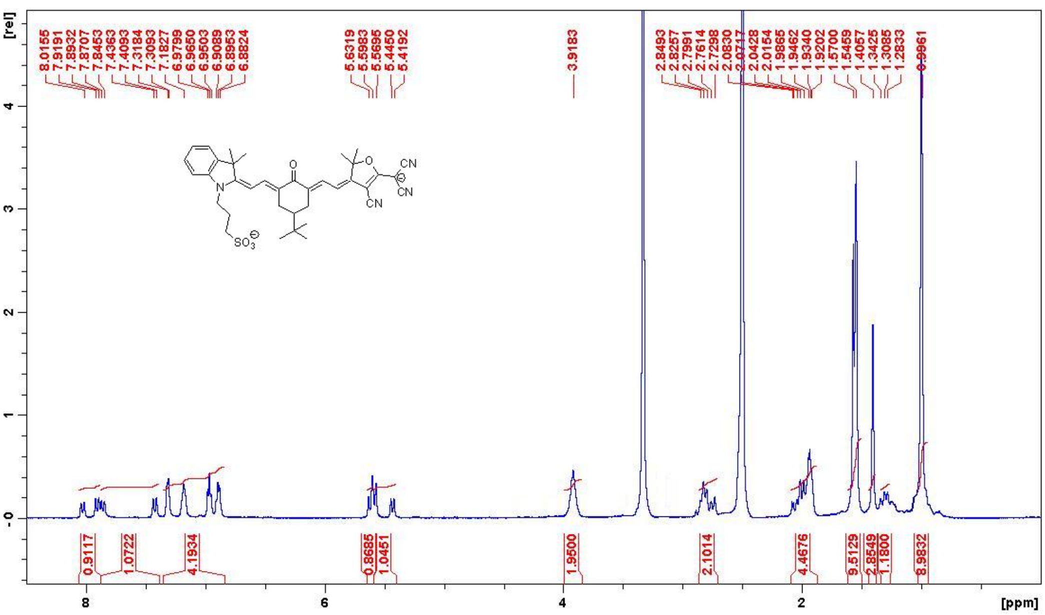

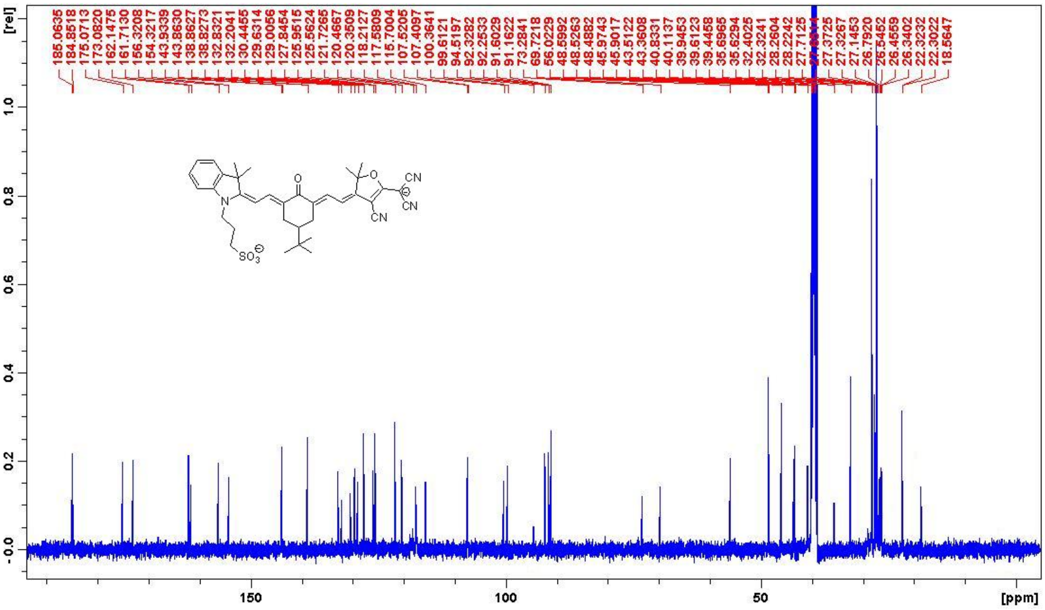
185.0635
184.8518
175.0713
173.0820
162.1475
161.7130
156.3208
154.3217
143.9339
143.8630
138.8627
138.8273
132.8321
132.2041
130.4455
129.6314
129.0056
127.8454
125.9515
125.5624
121.7265
120.4667
120.3509
118.2127
117.5809
115.7004
107.5205
107.4097
100.3641
99.6121
94.5197
92.3282
92.2533
91.6029
91.1622
73.2841
69.7218
56.0229
48.5992
48.5263
48.4882
45.9743
45.9017
43.5122
43.3608
40.8331
40.1137
39.9453
39.6123
39.4458
35.6965
35.6294
32.4025
32.3241
28.2604
28.2242
27.7125
27.3725
27.3267
27.1453
26.7920
26.5452
26.4559
26.3402
22.3232
22.3022
18.5647
[rel]
1.0
0.8
0.6
0.4
0.2
0.0
150
100
50
[ppm]
CN
CN
CN
N
O
O
SO_3^-

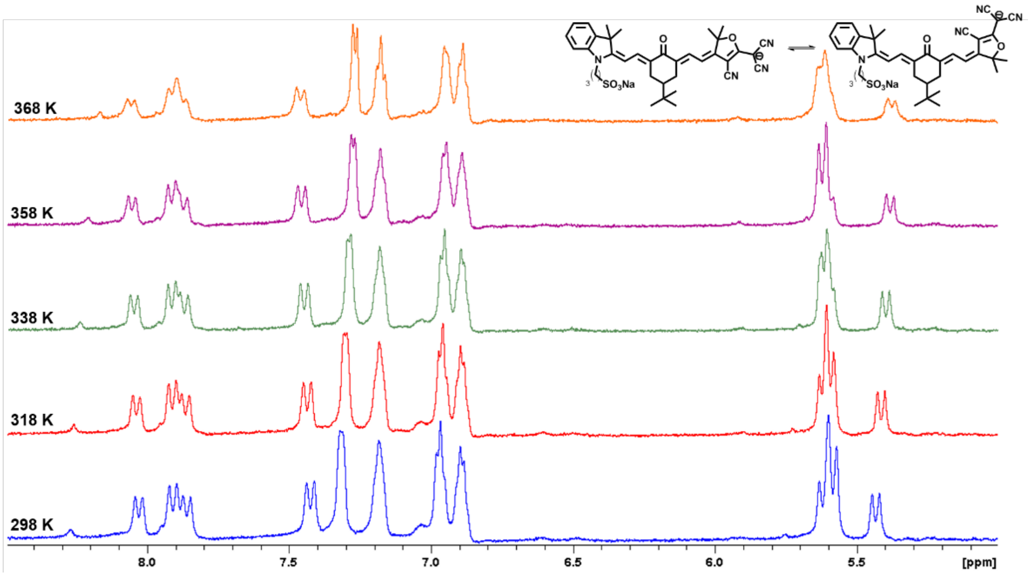
368 K
358 K
338 K
318 K
298 K
8.0
7.5
7.0
6.5
6.0
5.5
[ppm]
N
O
O
CN
CN
CN
NC
NC
SO_3Na
3

Compound AA=O

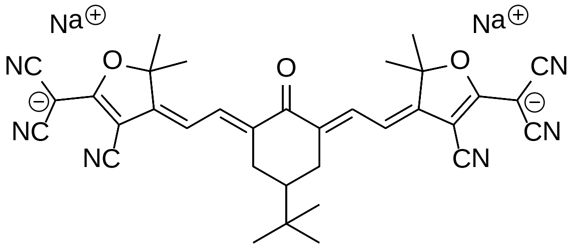

To a solution of 200 mg of **AA** (0.33 mmol, 1 equiv.) and 75 mg of *N*-hydroxysuccinimide (0.65 mmol, 2 equiv.) in 10 mL of anhydrous DMF was added 0.11 mL of distilled DIEA (0.65 mmol, 2 equiv.) and the mixture was stirred for 2 h at RT. The solvent was evaporated under reduced pressure, the residue was dissolved in 10 mL of MeOH, 489 mg of sodium iodide (3.26 mmol, 10 equiv.) were added and the mixture was stirred for 30 min at RT. The solvent was evaporated and the residue was dissolved in 50 mL of DCM and washed with water. The organic layer was dried over Na_2SO_4 and concentrated. After filtration trough a silica plug using DCM/MeOH as an eluent (9:1, R_f = 0.24), the product was isolated as a dark purple solid in a 80% yield (160 mg).

^{1}H NMR ($CD_3OD/CDCl_3$, 1:1, 500.10 MHz): δ 8.35 (d, ^3J = 13 Hz, 2H, =C**H**), 5.43 (d, ^3J = 13 Hz, 2H, =C**H**), 2.79 (d, ^2J = 14 Hz, 2H, $\mathbf{H}_{eq}$), 2.05 (dd, ^2J = 14 Hz, ^3J = 14 Hz, 2H, $\mathbf{H}_{ax}$), 1.48 (s, 12H, C(C**H**$_3$)$_2$), 1.41 (m, 1H, C**H**-C(CH$_3$)$_3$), 1.01 (s, 9H, C(C**H**$_3$)$_3$).

^{13}C NMR ($CD_3OD/CDCl_3$, 1:1, 125.75 MHz): δ 189.0 (C_{quat}), 178.3 (C_{quat}), 159.0 (C_{quat}), 135.3 (CH), 128.9 (C_{quat}), 120.4 (C_{quat}), 118.9 (C_{quat}), 118.6 (C_{quat}), 101.6 (CH), 93.8 (C_{quat}), 44.5 (CH), 38.7 (C_{quat}), 33.0 (C_{quat}), 28.0 (CH_3), 27.8 (CH_3), 27.3 (CH_2).

UV-Vis (CH_3OH): λ_{max} = 573 nm (ε_{max} = 60000 L.mol^{-1}.cm^{-1}).

UV-Vis (CH_3OH + CH_3CO_2H): λ_{max} = 804 nm (ε_{max} = 111000 L.mol^{-1}.cm^{-1}).

HRMS (ESI-): [M−2Na]$^{2-}$/2 = 285.1204 (calcd for $C_{34}H_{30}N_6O_3^{2-}$: 285.1195); [M+H−2Na]$^-$ = 571.2461 (calcd for $C_{34}H_{31}N_6O_3^-$: 571.2463).

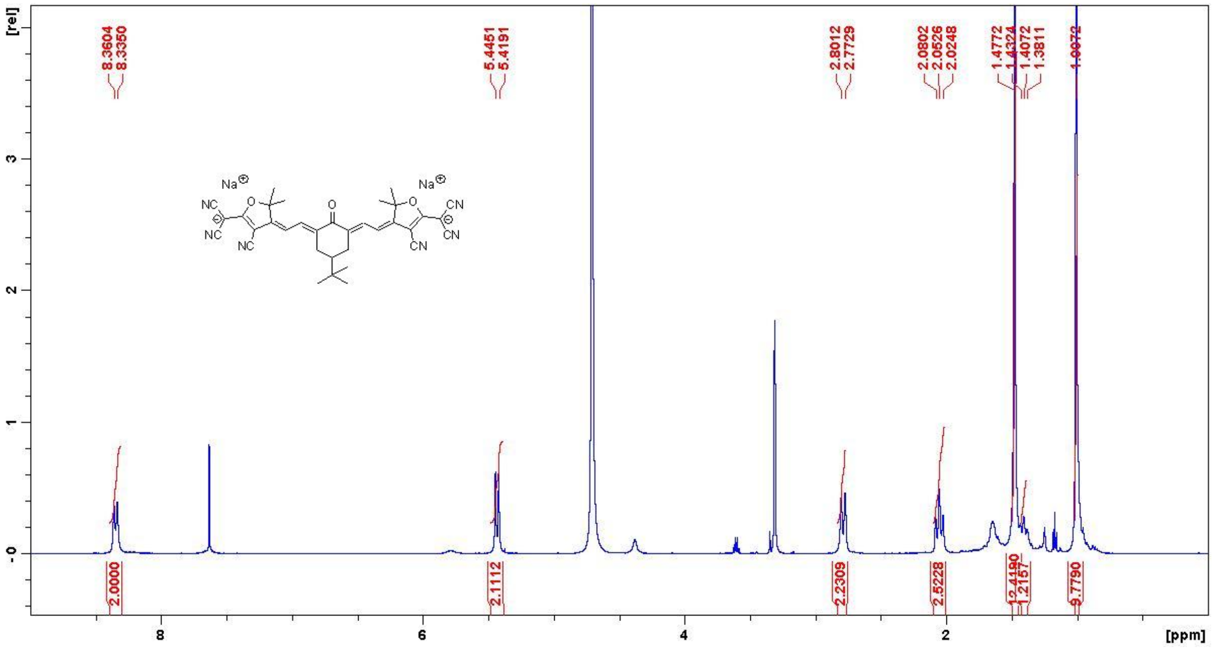

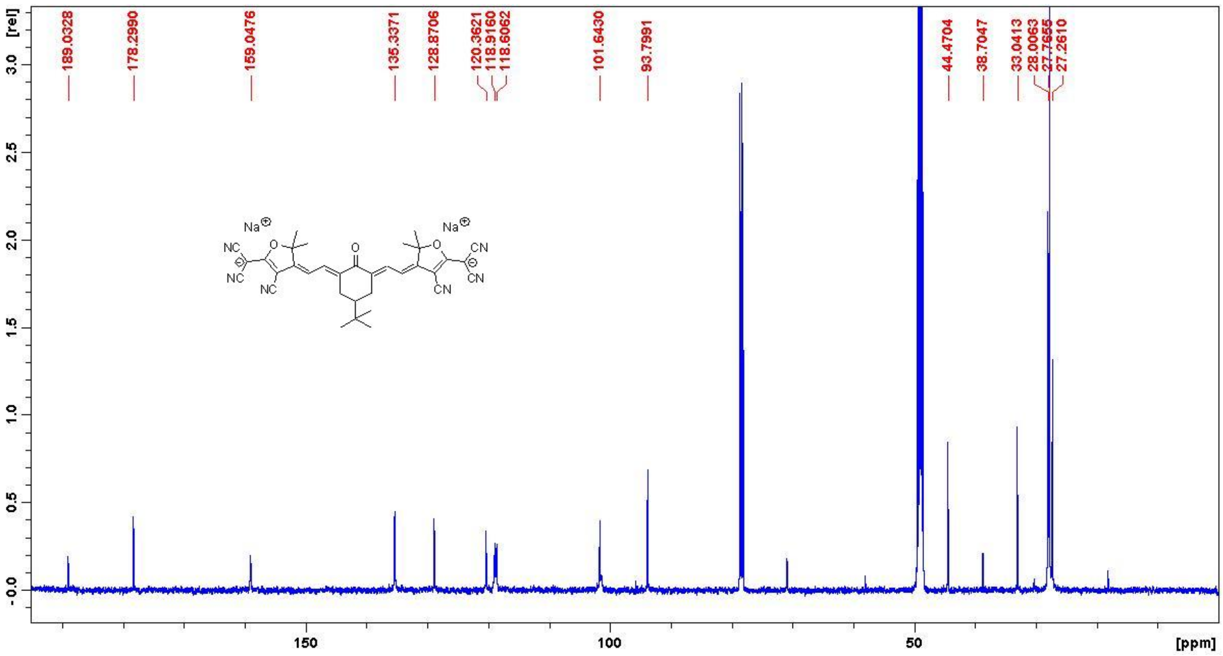

References

(1) Piotto, M.; Bourdonneau, M.; Elbayed, K.; Wieruszeski, J.-M.; Lippens, G., *Magn. Reson. Chem.* **2006,** *44*, 943-947.
(2) (a) Bouit, P.-A.; Wetzel, G.; Berginc, G.; Loiseaux, B.; Toupet, L.; Feneyrou, P.; Bretonnière, Y.; Kamada, K.; Maury, O.; Andraud, C., *Chem. Mater.* **2007,** *19*, 5325-5335.
(b) Tyler, A. R.; Okoh, A. O.; Lawrence, C. L.; Jones, V. C.; Moffatt, C.; Smith, R. B., *Eur. J. Med. Chem.* **2013,** *64*, 222-227.
(3) Flanagan, J. H.; Khan, S. H.; Menchen, S.; Soper, S. A.; Hammer, R. P., *Bioconjugate Chem.* **1997,** *8*, 751-756.
(4) Chen, X.; Peng, X.; Cui, A.; Wang, B.; Wang, L.; Zhang, R., *J. Photochem. Photobiol. A* **2006,** *181*, 79-85.
(5) Liu, D.; Chen, W.; Sun, K.; Deng, K.; Zhang, W.; Wang, Z.; Jiang, X., *Angew. Chem. Int. Ed.* **2011,** *50*, 4103-4107.
(6) Bouit, P.-A.; Di Piazza, E.; Rigaut, S.; Le Guennic, B.; Aronica, C.; Toupet, L.; Andraud, C.; Maury, O., *Org. Lett.* **2008,** *10*, 4159-4162.
(7) Gaussian 09, Revision D.01, M. J. Frisch, G. W. Trucks, H. B. Schlegel, G. E. Scuseria, M. A. Robb, J. R. Cheeseman, G. Scalmani, V. Barone, B. Mennucci, G. A. Petersson, H. Nakatsuji, M. Caricato, X. Li, H. P. Hratchian, A. F. Izmaylov, J. Bloino, G. Zheng, J. L. Sonnenberg, M. Hada, M. Ehara, K. Toyota, R. Fukuda, J. Hasegawa, M. Ishida, T. Nakajima, Y. Honda, O. Kitao, H. Nakai, T. Vreven, J. A. Montgomery, Jr., J. E. Peralta, F. Ogliaro, M. Bearpark, J. J. Heyd, E. Brothers, K. N. Kudin, V. N. Staroverov, R. Kobayashi, J. Normand, K. Raghavachari, A. Rendell, J. C. Burant, S. S. Iyengar, J. Tomasi, M. Cossi, N. Rega, J. M. Millam, M. Klene, J. E. Knox, J. B. Cross, V. Bakken, C. Adamo, J. Jaramillo, R. Gomperts, R. E. Stratmann, O. Yazyev, A. J. Austin, R. Cammi, C. Pomelli, J. W. Ochterski, R. L. Martin, K. Morokuma, V. G. Zakrzewski, G. A. Voth, P. Salvador, J. J. Dannenberg, S. Dapprich, A. D. Daniels, Ö. Farkas, J. B. Foresman, J. V. Ortiz, J. Cioslowski, and D. J. Fox, Gaussian, Inc., Wallingford CT, 2009.
